# Increased brain volume from higher cereal and lower coffee intake: shared genetic determinants and impacts on cognition and metabolism

**DOI:** 10.1093/cercor/bhac005

**Published:** 2022-02-07

**Authors:** Jujiao Kang, Tianye Jia, Zeyu Jiao, Chun Shen, Chao Xie, Wei Cheng, Barbara J Sahakian, David Waxman, Jianfeng Feng

**Affiliations:** Shanghai Center for Mathematical Sciences, Fudan University, Shanghai 200433, China; Institute of Science and Technology for Brain-Inspired Intelligence, Fudan University, Shanghai 200433, China; Key Laboratory of Computational Neuroscience and Brain-Inspired Intelligence (Fudan University), Ministry of Education, Fudan, Shanghai 200433, China; Institute of Science and Technology for Brain-Inspired Intelligence, Fudan University, Shanghai 200433, China; Key Laboratory of Computational Neuroscience and Brain-Inspired Intelligence (Fudan University), Ministry of Education, Fudan, Shanghai 200433, China; Centre for Population Neuroscience and Precision Medicine (PONS), Institute of Psychiatry, Psychology & Neuroscience, SGDP Centre, King’s College London, London SE5 8AF, United Kingdom; Shanghai Center for Mathematical Sciences, Fudan University, Shanghai 200433, China; Institute of Science and Technology for Brain-Inspired Intelligence, Fudan University, Shanghai 200433, China; Key Laboratory of Computational Neuroscience and Brain-Inspired Intelligence (Fudan University), Ministry of Education, Fudan, Shanghai 200433, China; Institute of Science and Technology for Brain-Inspired Intelligence, Fudan University, Shanghai 200433, China; Key Laboratory of Computational Neuroscience and Brain-Inspired Intelligence (Fudan University), Ministry of Education, Fudan, Shanghai 200433, China; Institute of Science and Technology for Brain-Inspired Intelligence, Fudan University, Shanghai 200433, China; Key Laboratory of Computational Neuroscience and Brain-Inspired Intelligence (Fudan University), Ministry of Education, Fudan, Shanghai 200433, China; Institute of Science and Technology for Brain-Inspired Intelligence, Fudan University, Shanghai 200433, China; Key Laboratory of Computational Neuroscience and Brain-Inspired Intelligence (Fudan University), Ministry of Education, Fudan, Shanghai 200433, China; Institute of Science and Technology for Brain-Inspired Intelligence, Fudan University, Shanghai 200433, China; Key Laboratory of Computational Neuroscience and Brain-Inspired Intelligence (Fudan University), Ministry of Education, Fudan, Shanghai 200433, China; Department of Psychiatry, University of Cambridge School of Clinical Medicine, Cambridge CB2 0SZ, United Kingdom; Department of the Behavioural and Clinical Neuroscience Institute, University of Cambridge, Cambridge CB2 3EB, United Kingdom; Institute of Science and Technology for Brain-Inspired Intelligence, Fudan University, Shanghai 200433, China; Key Laboratory of Computational Neuroscience and Brain-Inspired Intelligence (Fudan University), Ministry of Education, Fudan, Shanghai 200433, China; Shanghai Center for Mathematical Sciences, Fudan University, Shanghai 200433, China; Institute of Science and Technology for Brain-Inspired Intelligence, Fudan University, Shanghai 200433, China; Key Laboratory of Computational Neuroscience and Brain-Inspired Intelligence (Fudan University), Ministry of Education, Fudan, Shanghai 200433, China; Department of Computer Science, University of Warwick, Coventry CV4 7AL, United Kingdom

**Keywords:** Mendelian randomization, CPLX3 gene, genetic correlation, pattern correlation, gene expression in the brain

## Abstract

It is unclear how different diets may affect human brain development and if genetic and environmental factors play a part. We investigated diet effects in the UK Biobank data from 18,879 healthy adults and discovered anticorrelated brain-wide gray matter volume (GMV)-association patterns between coffee and cereal intake, coincidence with their anticorrelated genetic constructs. The Mendelian randomization approach further indicated a causal effect of higher coffee intake on reduced total GMV, which is likely through regulating the expression of genes responsible for synaptic development in the brain. The identified genetic factors may further affect people’s lifestyle habits and body/blood fat levels through the mediation of cereal/coffee intake, and the brain-wide expression pattern of gene CPLX3, a dedicated marker of subplate neurons that regulate cortical development and plasticity, may underlie the shared GMV-association patterns among the coffee/cereal intake and cognitive functions. All the main findings were successfully replicated. Our findings thus revealed that high-cereal and low-coffee diets shared similar brain and genetic constructs, leading to long-term beneficial associations regarding cognitive, body mass index (BMI), and other metabolic measures. This study has important implications for public health, especially during the pandemic, given the poorer outcomes of COVID-19 patients with greater BMIs.

## Introduction

Increases in human brain volume, due to growth, begin at an early stage of embryonic development and continue until late adolescence (Rapp and Bachevalier 2013). After this, the brain experiences a persistent but slow decrease in size throughout adulthood (Ziegler et al. 2012). Generally, development is tissue-specific but systematically organized across the brain (Ziegler et al. 2012; Tamnes and Østby 2018) and may be susceptible to both genetic and environmental influences (Ziegler et al. 2012; Fjell et al. 2015; Tamnes and Østby 2018; Satizabal et al. 2019; Zhao et al. 2019), as well as their interactions, e.g. through epigenetic modifications (Jia et al. 2019). Diet is a common environmental factor that can influence the trajectory of brain size. For example, a lack of nutrients over an extended period of time causes both structural and functional damage to the brain (Dewey and Begum 2011), and improved diet quality is associated with larger brain volumes (Croll et al. 2018). Furthermore, evidence suggested that ingested substances (both food and drink) in well-fed and healthy adults may also cause changes in brain size. For example, in a small-scale study, an increase in the size of the hippocampus was inferred to have occurred as an effect of both low and high coffee consumption (Perlaki et al. 2011).

While there are extensive studies of the degree to which different diets affect the body (Truswell 2002; Greely et al. 2008; Poole et al. 2017; van Dam et al. 2020), there is an absence of systematic investigation into how different diets may affect the human brain in both the short and long term. Thus, it is not known if impacts of different diets on brain structures follow similar patterns, or whether different brain regions exhibit differential sensitivity to diet and other environmental factors. In addition, there is a lack of knowledge about whether genetic factors play any role in the sensitivity of the brain to environmental factors. In the present research, consisting of an original study of 18,879 individuals and a replication study of 16,412 adults, we provide a detailed analysis of brain-size changes that occur in healthy adults due to the ingestion of different common foods and drinks. We investigated whether these influences from diets were systematically organized across the brain, whether these dietary influences have underlying genetic factors, and whether these genetic factors have further implications in people’s daily activities, metabolism, and cognitive functions.

## Materials and Methods

### Study participants

Study samples were from the UK Biobank study, a prospective epidemiological study that involves over 500,000 individuals in 22 centers across the United Kingdom (Sudlow et al. 2015). We included 431,039 White British individuals and then excluded 810 individuals who were diagnosed with Alzheimer’s or dementia (defined by codes G30/F00 in the 10th edition of the International Classification of Diseases) and 1 individual missing information of Alzheimer’s or dementia status. Of the 430,228 individuals left over, 336,517 individuals with quality-controlled genetic data were used to perform genome-wide association analyses. Meanwhile, 18,879 individuals with brain magnetic resonance imaging (MRI) data released as the first batch were used as the discovery sample, and the newly released (Smith et al. 2020) 16,412 individuals with brain MRI data were used as an independent replication sample. Detailed descriptions are available in Supplementary Methods.

### Assessment of the intake of cereal and coffee

Dietary information was obtained from the touchscreen questionnaire at the baseline and the MRI scan appointment. Cereal intake was defined as the number of bowls of cereal the participants consumed per week. The types of cereal included bran cereal, biscuit cereal, oat cereal, muesli, and other types (e.g. cornflakes, Frosties). Coffee intake was defined as the number of cups of coffee (including decaffeinated coffee, instant coffee, ground coffee, other types of coffee) the participants drank per day. Detailed information can be found in Supplementary Materials.

### Structural MRI data preprocessing

All structural MRI data were preprocessed in the Statistical Parametric Mapping package (Eickhoff et al. 2005) (SPM12) using the VBM8 toolbox with default settings, including the usage of high-dimensional spatial normalization with an already integrated Dartel template in the Montreal Neurological Institute space. All images were subjected to nonlinear modulations and corrected for each individual’s head size. Images were then smoothed with a 6 mm full-width at half-maximum Gaussian kernel with the resulting voxel size 1.5 mm^3^. The automated anatomical labeling 3 (AAL3) atlas (Rolls et al. 2019), which partitioned the brain into 166 regions of interest, was employed to obtain the total brain gray matter volume (GMV) and region-wise GMVs. The majority of discovery samples were assessed in the Cheadle MRI site (84.49%), with the rest in the Newcastle site (15.51%). In comparison, 37.17% of replication samples were assessed in the Cheadle site, while 37.87% were tested in the Newcastle site, with the remaining 24.96% were in the Reading site.

### Inference of causality based on a modified Mendelian randomization approach

Mendelian randomization has been shown as a powerful tool to establish random experiments based on genetic variants that are valid as instrumental variables that are directly linked to the independent variables only. An instrumental variable could represent different “trials” of a random experiment (of the independent variable) and hence leading to a causal inference on the outcome (i.e. the dependent variable) (Emdin et al. 2017). However, due to the lack of knowledge about which single-nucleotide polymorphisms (SNPs) could serve as instrumental variables that only directly link to the independent variable but not the dependent variable, it is generally very difficult to perform the Mendelian randomization on quantitative phenotypes without the confounding of genetic pleiotropy (the same genetic variant may influence both phenotypes of questions independently) and the risk of reverse causality (the dependent variable may reversely also have causal influence on the independent variable). Therefore, in many occasions, the interpretation of Mendelian randomization results heavily relied on widely accepted hypothetic causal relationship, thus paradoxically, these applications of Mendelian randomization do not serve its original purpose as a tool to infer causality without any pre-knowledge.

Nevertheless, as the influence of genetic variants on the dependent variable (as an indirect effect through the independent variable) should always be lower than that on the directly influenced independent variable, by gradually removing SNPs that cross-associated with both independent and dependent variables based on varied threshold, we could eventually keep only SNPs influencing the independent variables, thus valid as instrumental variables (Burgess et al. 2020). Further, this approach is unbiased in that instrumental variables for both dependent and independent variables could be acquired simultaneously, and therefore, a bi-directional causal inference could be conducted, which directly addresses the issue of reverse causal inference in traditional Mendelian randomization approaches.

Finally, we employed the polygenic risk score (PRS), in combination with the above process to remove pleiotropy, as the instrumental variable to integrate contribution from multiple genetic variants, instead of using a single SNP, to enlarge the difference between “trials” randomized by the instrumental variable.

### Mediation analysis

Mediation effects were examined using Baron and Kenny's (1986) causal steps approach (see Supplementary Methods for details). To further evaluate the *P*-value of the significant mediation identified by the causal steps approach, we performed 1,000 times bootstrap of the individuals to obtain the distribution of the proportion of the mediation, i.e.$\mathrm{PM}=(\tau -{\tau}^{\prime})/\tau$ (τ is the total effect and τ’ is the direct effect), under the alternative hypothesis. Thus, the PM was expected to be positive by definition, and the corresponding *P*-value could be calculated as the doubled chance of observing the PM less than zero during the 1000-bootstrap procedure. As no priory assumption about whether diet or lifestyle/blood and body fat levels should serve as the mediator for their associations with the lead SNPs, we, therefore, identified the most likely mediator with an excess PM, i.e. the model showing higher PM, of which the significance level was again evaluated through a 1000-bootstrap process.

### Pattern similarity analysis

We examined the similarity among the brain-wide GMV-association patterns of cereal/coffee intake and cognitive functions. Specifically, we first performed association analyses between region-wide GMV and each phenotype. Then, we calculated the Pearson correlation coefficient (similarity) between the GMV-association patterns of a pair of phenotypes of interest, of which the significance level was evaluated through 10,000 times permutation that shuffled the individual’s IDs of the GMV data at each iteration.

The similarity between brain-wide GMV-association pattern of a given phenotype and the brain-wide gene expression pattern was also examined through their pattern correlation, of which the null distribution was established through 10,000 times permutation that at each iteration, the pattern correlation was recalculated with the GMV-association patterns been regenerated with shuffled IDs of the GMV data. The corresponding *P*-values were hence calculated as the chance of randomly getting a higher pattern correlation than the observed one in terms of their absolute value based on the established null distribution. The above permutation process was employed to ensure that the potential oversampling of brain regions will not inflate the false positive rate.

### Analyses overview

**Figure 1:**
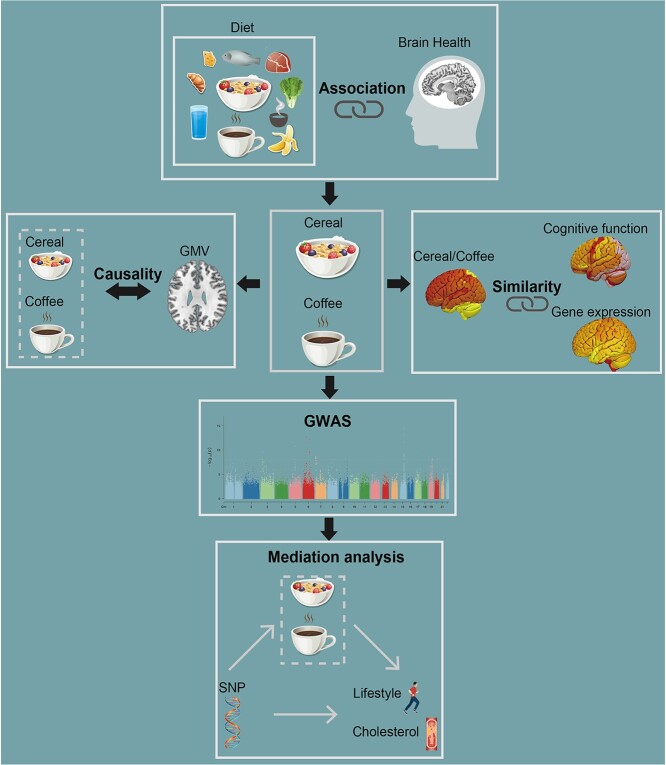
Flow diagram of the analyses pipeline used in the study.

Figure 1 outlines the analytical pipeline of the present study. We first tested the associations of 17 diet phenotypes with the total brain gray matter volume (TGMV). The following analyses then focused on the intake of cereal and coffee, given their significant correlations with the TGMV. Next, we applied a modified Mendelian randomization analysis to investigate the potential causality between the cereal/coffee intake and the TGMV. We further conducted a series of pattern correlation analyses to investigate the similarity among the GMV-association patterns of cereal/coffee intake and cognitive function, as well as with the gene expression pattern in the brain, to better characterize the impact of cereal/coffee intake on the GMV. Genome-wide association analyses were also conducted to ascertain putative genetic variants accounted for the cereal/coffee intake. Finally, mediation analyses were applied to determine the relationship between genetics, cereal/coffee intake, lifestyle, and metabolic traits.

## Results

### Participant characteristics

Of the discovery sample with both dietary information and neuroimaging (*N* = 18,879), participants’ mean age was 55.1 (SD = 7.46) at baseline; the majority of participants were women (52.39%); the average cups of coffee consumed at baseline were 2.07 (SD = 1.92); the average bowls of cereal consumed at baseline were 4.85 (SD = 2.66). Of the replication sample (*N* = 16,412), participants’ mean age was 54.9 (SD = 7.37) at baseline; the majority of participants were women (52.74%); the average cups of coffee consumed were 2.00 (SD = 1.80); the average bowls of cereal consumed were 4.78 (SD = 2.69). Detailed characteristics of the discovery sample and replication sample (for both baseline and follow-up) are provided in Supplementary Tables 1 and 2.

### Association between GMV and diets

We first investigated the relationship between GMV and 17 different diet phenotypes, which were both measured at the second visit (i.e. at follow-up) of participants to a research center (Sudlow et al. 2015). We found that the TGMV is associated with diet. With a statistically significant correlation (*P* < 0.05 Bonferroni corrected), the intake of coffee, water, processed meat, beef, lamb/mutton, and pork was found to be negatively correlated with TGMV, while the intake of cereal and dried fruit was positively correlated with TGMV (Supplementary Table 3). These correlations were largely intact after controlling for average total household income, qualifications, and Townsend deprivation index, which demonstrates that this relationship is not solely driven by socioeconomic factors. We note that predated measurements (i.e. baseline measurements) of cereal and coffee intake were also related to the follow-up values of TGMV, and these remained significant even after controlling for the corresponding follow-up intakes. This indicates a persistent, rather than a short-term connection between diet and GMV. We then validated the above results in the newly released additional 16,412 UK Biobank individuals and confirmed the persistent positive associations between TGMV and cereal intake and negative associations between TGMV and coffee intake (Supplementary Table 4).

**Figure 2:**
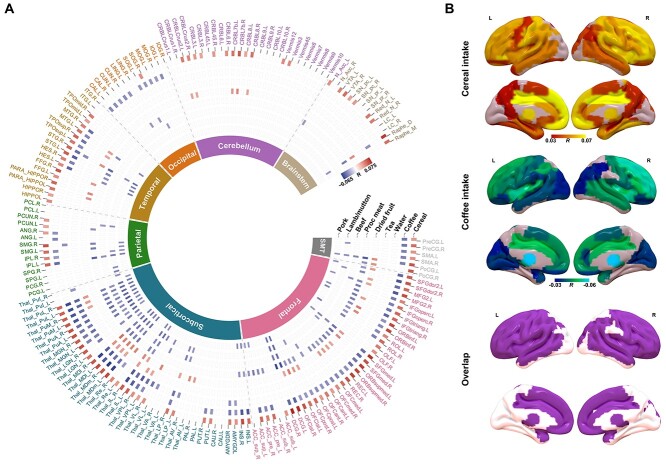
Correlations between GMV and different daily diets. A) Circular heatmap of correlations between GMVs of 166 brain regions from AAL3 (the outer layer) and different diets (along radius). As indicated by the color bar, positive correlations were highlighted in red while negative correlations were highlighted in blue. The inner layer indicates the lobes that brain regions belong to. B) Brain regions with significant correlations between their GMVs and the intake of cereal (upper) and coffee (middle), as well as the overlapped significant regions (bottom). SM, sensorimotor.

We further investigated the correlation between diet phenotypes and the regional GMV. A total of 454 statistically significant correlations (Bonferroni correction: *P* < 0.05/166/17) were found, again mainly between GMV and intake of cereal, coffee, water, dried fruit, processed meat, beef, pork, and lamb/mutton (Fig. 2A). Notably, the GMV-association pattern of cereal intake highly resembles, although in the opposite direction, the GMV-association pattern of coffee intake (pattern correlation across the whole brain: *r* = −0.6177, *P* < 1E−04 based on 10,000 permutations; Fig. 2B), the same negative pattern correlation could also be observed in the replication sample (*r* = −0.45, *P*_one-tailed_ = 0.0116 based on 10,000 permutations).

### Genome-wide association studies for the intake of cereal and coffee

**Figure 3:**
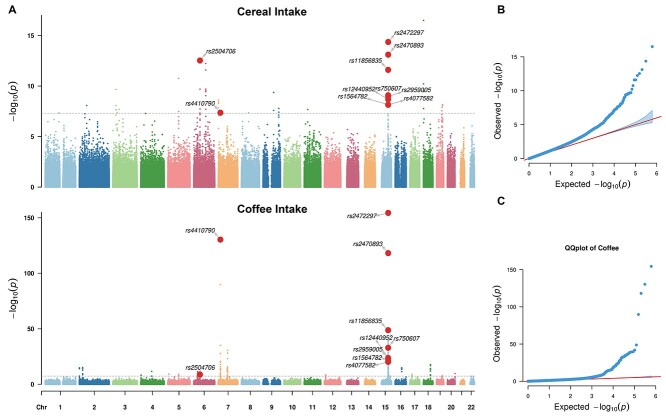
Results of GWAS of the intake of cereal and coffee. A) Manhattan plots of the GWAS results for cereal (upper) and coffee (bottom) intake. The genome-wide significance level was indicated by gray lines (i.e. *P*-value = 5E−08). Variants with significant associations for both cereal and coffee intake were highlighted as red dots. B) QQ-plots for the GWAS of cereal intake. C) QQ-plots for the GWAS of coffee intake. The corresponding genomic inflation factors were 1.256 (cereal intake) and 1.201 (coffee intake). Please be noted that the inflations of both QQ-plots were not due to unknown population stratifications, which was confirmed by the corresponding LD regression with intercepts estimated as 1.013 (cereal intake) and 1.005 (coffee intake).

**Figure 4:**
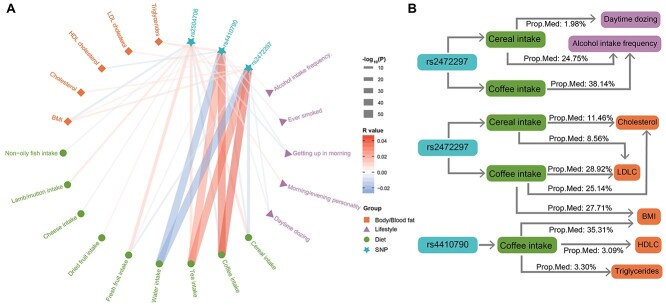
Relationships between the lead SNPs and diets, lifestyle, and body/blood fat. A) Associations between the three lead SNPs (of both cereal and coffee intake) and other diets, lifestyle, and body/blood fat levels. The color of each line represents the correlation coefficient (positive in red and negative in blue), and the thickness of each line represents the −log_10_  *P*-value (capped at 50) of the corresponding correlation. B) Proposed mediation models of genetic variants, body/blood fat levels, lifestyles, and the intake of cereal and coffee. Prop. Med, proportion of mediation; BMI, body mass index; HDLC, high-density lipoprotein cholesterol; LDLC, low-density lipoprotein cholesterol.

In total, 336,517 individuals with quality-controlled genetic data were included in the following analyses. We conducted genome-wide association studies (GWAS) for the intake of both cereal (*n* = 335,696) and coffee (*n* = 335,068) at baseline and identified 21 and 45 independent lead genome-wide significant variants (i.e. the lead SNPs, see Supplementary Methods for details), respectively (Fig. 3). The genomic inflation factors (λ_GC_) were 1.256 (cereal intake) and 1.201 (coffee intake). However, a linkage disequilibrium (LD) score regression (Bulik-Sullivan BK et al. 2015b) analysis indicates that both findings were free from systematically inflated false-positive rates, e.g. due to population stratification, with intercepts of 1.013 (cereal intake) and 1.005 (coffee intake). We observed a significant negative genetic correlation (Bulik-Sullivan B et al. 2015a) between intake of cereal and coffee (*r*_g_ = −0.233, *z*-score = −4.49, *P* = 7.1E−06), i.e. the alleles associated with higher cereal intake were likely to be in association with reduced coffee intake, which is in line with the above GWAS findings, where the three shared lead SNPs, i.e. rs2504706, rs4410790, and rs2472297, were found in associations with both cereal and coffee intake, again in opposite directions (Supplementary Tables 5 and 6). While rs4410790 and rs2472297 have both been previously associated with coffee/caffeine consumption (Cornelis et al. 2011, 2015; Sulem et al. 2011), caffeine metabolism (Cornelis et al. 2016), and alcohol consumption (Liu et al. 2019), this is the first study to identify an association with cereal intake. Additionally, rs4410790 (the C-allele) and rs2472297 (the T-allele) were also strongly associated with higher intake of tea and lower intake of water (Fig. 4A and Supplementary Table 7), although both intakes were not observed with significant long-term impacts on the TGMV. This result is remarkable because there is a median to large anti-correlation between the intake of coffee and tea (*r* = −0.359, β = −0.472, *T*_df = *332,711*_ = −221.65, *P* < 1.0E−256), which is likely due to the seesaw effect given the limited amounts of beverages one may consume each day. Thus, individuals with both SNPs (i.e. C-allele of rs4410790 and T-allele of rs2472297) might generally prefer flavored beverages to the water.

### Causal effects of cereal/coffee intake on TGMV based on Mendelian randomization

Above, we have established and verified the associations of cereal/coffee intake with TGMV. Here, we further implemented a modified Mendelian randomization approach to investigate potential causal relationship by introducing nonpleiotropic PRSs (i.e. the valid-PRS of cereal/coffee intake or TGMV) as instrument variables of randomized experiments, which only affect the explanatory variables, but not the outcome variables if not through the explanatory variables (see Methods for more details; Davies et al. 2018).

**Figure 5:**
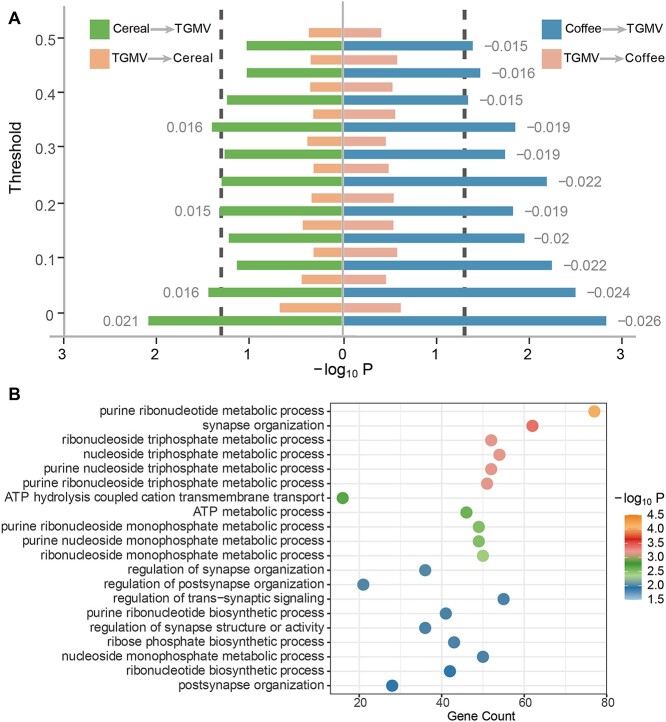
Causality between cereal/coffee intake and TGMV. A) The left (right) part is the causality between the cereal (coffee) intake and TGMV. Each bar represents the −log_10_  *P*-value of a correlation between the PRS and phenotype. The *y*-axis is the threshold of p value used to remove pleiotropy. B) Top 20 GO terms enriched for the transcription-related specific genes.

Using the availability of neuroimaging data as a random stratification of UK Biobank data, we reconducted GWAS of cereal/coffee intake in the large discovery sample without neuroimaging information (*N*= 308,839), as well as conducting a new GWAS of TGMV in the first batch of neuroimaging data (*N* = 14,807). In the independent second batch of neuroimaging data (*N* = 12,783), we then calculated the PRSs of cereal/coffee intake and TGMV (using PRSice v1.25 (Euesden et al. 2015) with the *P*-value threshold of 0.05, Supplementary Table 8 and Fig. 5A), which all showed consistent associations with the corresponding phenotypes (PRS_cereal_ with cereal intake: *R* = 0.081, *P*_one-tailed_ = 2.13E−20; PRS_coffee_ with coffee intake: *R* = 0.075, *P*_one-tailed_ = 1.31E−17; PRS_TGMV_ with TGMV: *R* = 0.097, *P*_one-tailed_ = 4.39E−28). Interestingly, while PRS_cereal_ and PRS_coffee_ were found in significant associations with TGMV (*R* = 0.021, *P*_one-tailed_ = 8.20E−3 and *R* = −0.026, *P*_one-tailed_ = 1.48E−3, respectively), PRS_TGMV_ was not associated with the intake of either cereal (*R* = 0.007, *P*_one-tailed_ = 0.215) or coffee (*R* = −0.006, *P*_one-tailed_ = 0.244), indicating that genetically TGMV might be secondary to the intake of cereal/coffee. Furthermore, we removed potential pleiotropic SNPs from the above PRSs with a stepwise approach (i.e. stepwise removing cross-associated SNPs based on their *P*-values with the confounding phenotypes from 0.05 to 0.50 with a step of 0.05) and found these valid-PRSs maintained strong correlations with the corresponding main phenotypes (valid-PRS_cereal_ with cereal intake: *R* > 0.057, *P*_one-tailed_ < 6.16E−11; valid-PRS_coffee_ with coffee intake: *R* > 0.059, *P*_one-tailed_ < 1.07E−11; valid-PRS_TGMV_ with TGMV: *R* > 0.059, *P*_one-tailed_ < 1.26E−11), hence as qualified instrumental variables of random experiments. However, while valid-PRS_coffee_ maintained consistently significant negative associations with TGMV across all 10 thresholds (*R* < −0.015, *P*_one-tailed_ < 0.046), valid-PRS_cereal_ lost the significance with TGMV in 7 out of 10 thresholds although maintaining consistent positive associations throughout (*R* > 0.012, *P*_one-tailed_ < 0.095). Nevertheless, the associations of valid-PRS_TGMV_ with the intake of cereal (−0.001 < *R* < 0.003, *P*_one-tailed_ > 0.370) and coffee (−0.006 < *R* < −0.002, *P*_one-tailed_ > 0.266) further diminished. Thus, following the argument of Mendelian randomization, we could reach a conclusion that randomly increased coffee intake will cause reduced TGMV, but not the other way around. Further, the results obtained using the inverse-variance weighted method (Supplementary Methods) reach the same conclusion regarding a harmful causal effect of coffee intake on the TGMV (β = −5.420; SE = 1.649; *P* = 1.01E−03) (see Supplementary Results for more details).

### Transcriptome-wide whole-brain pattern analyses of the coffee intake’s impact on the GMV

To explore the potential neurobiological mechanisms underlying the causal effects of coffee intake on the TGMV, we next investigate if coffee intake may regulate gene expression pertaining to synaptic development. Particularly, we evaluated the similarity between the GMV-association pattern of the coffee intake and the spatial gene expression pattern from the Allen Institute for Brain Science (Hawrylycz et al. 2012) and identified 1,737 validated significant genes (*P*_FDR,perm_ < 0.05 in the discovery sample and *P*_perm_ < 0.05 in the replication sample; see Section 2 for details). Out of these 1,737 genes, 15 were overlapped with the 199 candidate genes identified through the coffee intake GWAS (Supplementary Methods) and were mainly enriched in the perception of bitter taste (*P*_FDR_ < 0.001) based on a follow-up Gene Ontology (GO) analysis (Yu et al. 2012), which might highlight a potential complicated gene–environmental interaction that the same gene may influence its own expression through regulating the coffee intake. Further, the remaining nonoverlapped 1,722 genes were highly enriched in biological pathways related to synapse organizations (best *P*_FDR_ = 4.18E−4, Supplementary Table 9), thus highlighting that coffee intake could regulate the expression of these synaptic genes in the brain, which may explain the biological mechanism underlying the causal effect of coffee intake on the TGMV (Fig. 5B).

### Association between genetic variants, diets, and lifestyle

As both cereal and coffee intake, as well as their shared lead SNPs, were associated with different lifestyles, such as the frequency of physical activity (*R* = 0.016, *P* = 2.52E−17 for cereal and *R* = −0.011, *P* = 3.23E−09 for coffee), being a morning/evening person (*R* = −0.040, *P* = 2.57E−104 for cereal and *R* = 0.032, *P* = 3.05E−69 for coffee) and the frequency of alcohol use (*R* = −0.101, *P* < 1.0E−256 for cereal and *R* = 0.050, *P* = 5.77E−184 for coffee) (Fig. 4A and Supplementary Tables 10 and 11), we then investigated possible mediation roles of diet or/and lifestyles on their associations with SNPs. As no prior assumptions about whether diet or lifestyle should serve as the mediator for their associations with the lead SNPs, we evaluated the most likely mediator, based on the corresponding proportion of mediation (PM) that they are responsible for (Supplementary Methods, Supplementary Table 12 and Fig. 4B). We found the following:

(1) Both intake of cereal and coffee were likely to mediate the positive association of the frequency of alcohol intake with the T-allele of rs2472297 (PM = 24.75%, *P*_bootstrap_ = 5.47E−15 and PM = 38.14%, *P*_bootstrap_ = 1.37E−82, respectively); these were superior to alternative mediation models with the frequency of alcohol intake as the mediator (excess PM > 20% with *P*_bootstrap_ < 0.002 for both alternative models).
(2) The association between higher T-alleles of rs2472297 and less daytime sleeping was mediated by cereal intake (PM = 1.98%, *P*_bootstrap_ = 2.82E-6), which was superior to the alternative mediation model with daytime sleeping as the mediator (excess PM = 1.39% with *P*_bootstrap_ = 0.018).
(3) Both difficult in rising and less daytime sleeping were found to mediate the negative association of cereal intake with the C-allele of rs4410790, so did the alternative mediation models with the cereal intake as the mediator. However, neither group of mediation models was superior to the other.
(4) Interestingly, while individuals with rs2504706 (the C-allele) were more likely to be an “evening person” and experience difficulties in rising, both lifestyle traits did not mediate the associations of the SNP with higher cereal intake or lower coffee intake (nor did the alternative mediation models), which was mainly due to nonconcordant correlations, e.g. a positive correlation was observed between ease in rising and higher cereal intake while their associations with SNP rs2504706 would indicate a negative correlation instead.

### Association between genetic variants, diets, and metabolic measures

In addition to lifestyle, both cereal and coffee intake, as well as their shared lead SNPs, were also associated with blood (e.g. with total cholesterol, *R* = −0.066, *P* < 1.0E−256 for cereal and *R* = 0.045, *P* = 1.89E−139 for coffee) and body fat levels (e.g. with the body mass index (BMI), *R* = −0.076, *P* < 1.0E−256 for cereal and *R* = 0.053, *P* = 3.84E−206 for coffee) (Fig. 4A and Supplementary Tables 13 and 14). Therefore, we further explored possible mediator roles of fat levels and the intake of cereal and coffee (Supplementary Methods, Supplementary Table 15 and Fig. 4B). We found the following:

(1) Associations between rs4410790 (C-allele) and an increased BMI, triglycerides, and decreased HDL cholesterol were mediated by increased coffee intake (PM = 35.31%, *P*_bootstrap_ = 1.41E−81; PM = 3.30%, *P*_bootstrap_ = 1.04E−5; and PM = 3.09%, *P*_bootstrap_ = 2.28 E−4, respectively), which were superior to the alternative mediation models with corresponding fat levels as mediators (excess PMs = 34.48%, 3.10%, and 2.95%, respectively; all corresponding *P*_bootstrap_ < 0.002);
(2) Associations between rs2472297 (T-allele) and higher BMI, total cholesterol, and LDL cholesterol were mediated by higher coffee intake (PM = 27.71%, *P*_bootstrap_ = 6.72E−83; PM = 25.14%, *P*_bootstrap_ = 6.82E −68; and PM = 28.92%, *P*_bootstrap_ = 4.65E−75, respectively), as well as by lower cereal intake, to a lesser extent (PM = 11.46% for total cholesterol, *P*_bootstrap_ = 6.51E−14 and PM = 8.56% for LDL cholesterol, *P*_bootstrap_ = 1.80E−13). The above models were superior to alternative mediation models with corresponding fat levels as mediators (for the coffee intake: excess PMs = 26.66%, 24.38%, and 28.13%, respectively, with all corresponding *P*_bootstrap_ < 0.002; for the cereal intake: excess PMs = 7.54% and 5.83%, respectively, with all corresponding *P*_bootstrap_ < 0.05).

Related to the current COVID-19 pandemic, using the UK Biobank data we found that individuals who tested positive of COVID-19 (*n* = 639, inpatients only) had higher BMIs (Cohen’s *d* = 0.27, *t* = 6.72, *P* = 1.86E−11) and lower cereal intake (Cohen’s *d* = −0.09, *t* = −2.36, *P* = 0.019) than the rest population (*n* = 314,982, either tested negative or not tested). This further highlights the importance of our finding for public health that cereal intake is associated with lower BMIs.

### Associations between the GMV-association patterns of cognitive functions and the GMV-association patterns of the intake of cereal and coffee

**Figure 6:**
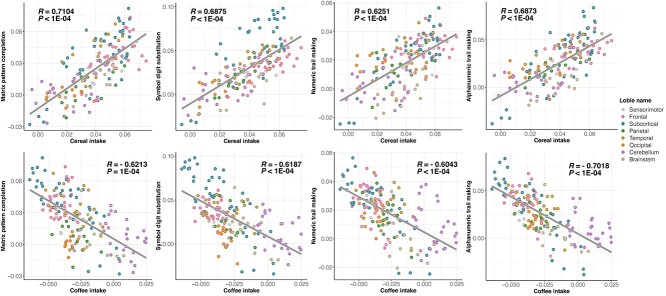
Scatter plots of brain-wide GMV-association patterns of cognitive functions and the intake of cereal (upper) and coffee (bottom). Each dot represents one of 166 AAL3 brain regions, where the colors indicate at which lobes the brain regions were located. Detailed brain-wide GMV-association patterns of cognitive functions and cereal/coffee intake were available in Supplementary Table 21.

To further characterize the negatively correlated brain-wide GMV-association patterns for cereal and coffee intakes, we further investigated if such similarities have any implications for cognitive functions, and we found that brain-wide GMV-association patterns of most cognitive functions were significantly correlated with those of both cereal and coffee intake, although in opposite directions, at both baseline and follow-up (GMV were measured at follow-up only) (Fig. 6 and Supplementary Table 16). In particular, performance in tasks of matrix pattern completion, symbol digit substitution, and numeric and alphabet-numeric trail making showed similar brain-wide GMV-association patterns with both cereal (in positive correlation) and coffee (in negative correlation) intake at both baseline and follow-up (|R|_min_ = 0.5945, all *P*_FDR_ < 0.05), while the fluid intelligence score only showed a similar brain-wide GMV association pattern with the cereal intake (at both baseline and follow-up; *R*_min_ = 0.62, all *P*_FDR_ < 0.05). The same findings could be replicated in the replication sample (|R|_min_ = 0.40, *P*_one-tailed_ < 0.01) (Supplementary Table 17). In line with the above findings, higher risk of Alzheimer’s disease (estimated as the proxy-AD (Jansen et al. 2019), Supplementary Materials), characterized by reduced cognitive functions, was associated with reduced cereal intake (*R* = −0.009, *P* = 3.42E−6), as well as increased coffee intake to a much lesser extent (*R* = 0.004, *P* = 0.024), in contrast to previous findings of either protective (Poole et al. 2017) or nonsignificant (Larsson and Orsini 2018) effect of high coffee intake on Alzheimer’s disease.

### Associations between the GMV-association patterns of the cereal/coffee intake and the gene expression patterns of expression quantitative trait loci (eQTL) genes

Finally, we investigated if the identified putative genetic variants associated with both cereal and coffee intake may also contribute to observed similarities of brain-wide GMV association patterns between diet and cognitive function. We first performed eQTL mapping of the 3 shared lead SNPs using FUMA (Watanabe et al. 2017) software and identified 31 candidate protein-coding genes that also have brain-wise gene expression information from the Allen Institute for Brain Science (AIBS) (Hawrylycz et al. 2012). The brain-wide expression pattern for each candidate gene was then correlated with the brain-wide GMV association patterns for cereal/coffee intakes. While multiple candidate genes had their brain-wide expression pattern in significant correlation with brain-wide GMV associations patterns for the coffee intake (Supplementary Table 18), only gene CPLX3 showed significant “gene expression vs. GMV-association” pattern similarity with both intakes of cereal (*R* = 0.47, *P*_perm_ = 2.9E−3, *P*_FDR-corrected_ = 0.033) and coffee (*R* = −0.44, *P*_perm_ = 7.2E−3, *P*_FDR-corrected_ = 0.046). Both findings could be reproduced in the replication sample (*R* = 0.40, *P*_one-tailed_ = 0.011 for cereal intake; *R* = −0.36, *P*_one-tailed_ = 0.020 for coffee intake; 10,000 permutations; Supplementary Table 20). It is of particular interest that the gene expression of CPLX3 (a known prominent marker specific for subplate neurons in the brain that regulate cortical development and plasticity across the brain; Kanold and Shatz 2006; Kanold 2009; Viswanathan et al. 2017) also showed significant pattern correlations with almost all cognitive functions (i.e. *R* = 0.42 for fluid intelligence, *R* = 0.49 for numerical memory, *R* = 0.44 for prospective memory, *R* = 0.46 for matrix pattern completion, *R* = 0.39 for symbol digit substitution, and *R* = 0.44/*R* = 0.55 for both trail making tasks; all corresponding *P*_FDR-corrected_ < 0.05; Supplementary Table 19). The above findings were again fully reproducible in the replication sample (*R*_min_ = 0.39, *P*_one-tailed_ < 0.011) (Supplementary Table 20).

## Discussion

In the large-scale imaging/genetics analysis presented in this work, we have (i) gained insights into long-term associations between brain-wide GMV and diets, especially the anticorrelated impacts from cereal and coffee intake; (ii) identified shared genetic constructs for both higher cereal and lower coffee intake and confirmed the causal effect of coffee intake on the brain volume, which is likely through regulating the expression of genes responsible for synaptic development; (iii) explored the complex relationship among cereal/coffee intake, their genetics constructs, lifestyle, and body/blood fat level; (iv) revealed shared brain-wide GMV-association patterns between cognitive function and the intake of cereal and coffee and further showed that such similarity might be underlaid by the brain-wide expression patterns of gene CPLX3, a shared genetic determinant identified for the intake of both cereal and coffee. These novel findings hence suggest the existence of a brain-wide systematic organization of GMV that is susceptible to both genetic and environmental influences, as well as their complex interaction, which may have further impacts on people’s lifestyles, cognitive functions, and metabolic measures (e.g. BMI and blood cholesterol level).

Two lead SNPs shared by the intake of coffee and cereal, i.e. rs4410790 and rs2472297, have been previously associated with coffee/caffeine consumption (Cornelis et al. 2011, 2015; Sulem et al. 2011), caffeine metabolism (Cornelis et al. 2016), and alcohol consumption (Liu et al. 2019). However, this is the first study to identify their associations with cereal intake. Moreover, while CPLX3, within the LD complexity around the lead SNP rs2472297, has previously been proposed as a candidate gene for both coffee consumption (Amin et al. 2012) and blood pressure (Evangelou et al. 2018), it is the first time that this gene links to multiple cognitive functions, such as intelligence, and cereal intake. Remarkably, the expression of CPLX3 is a highly specific marker of subplate neurons (Viswanathan et al. 2017) that regulate cortical development and neuronal plasticity across the brain (Kanold and Shatz 2006; Kanold 2009). Specifically, while most subplate neurons were short-lived during the development of the brain, previous studies have shown that the Cplx3-positive subplate neurons could survive into adulthood in mice (Viswanathan et al. 2017). Therefore, our findings were not only congruent with the role of subplate neurons in the cortical development, but with further implications that these CPLX3-positive subplate neurons might mark the dynamic system of GMV in the brain that is susceptible to environmental factors. Such a hypothesis could be supported by previous findings that Cplx3 protein’s regulation of exocytosis in mice retinal neurons could be altered by both light and electrical stimuli (Babai et al. 2016; Mortensen et al. 2016).

Our findings have to be understood in the context of the following limitations. First, the GWAS sample size of the total GMV was relatively small compared to the GWAS sample of cereal/coffee intake. Therefore, replication of these results in additional studies is necessary to consolidate the conclusion further. Second, the PRS is a cumulative measure of genomic burden, and the detailed genetic mechanisms underlying the behavioral consequences need to be explored further. Third, the PRS in the present study was calculated based on SNPs only and did not take into account other genetic variants such as CNVs or rare mutations. Thus, studies integrating effects of multiple types genetics variants should be conducted in the future.

Overall, since high cereal diets, but low coffee diets, have long-term beneficial associations regarding the brain, cognition, BMI, and other metabolic measures, this study has significant implications for public health. Our findings highlight the importance of a “cereal” breakfast across the life span, but perhaps especially for children and adolescents whose brains are still in development and for reducing the risk of Alzheimer’s disease and poor outcomes due to high BMIs in patients with COVID-19 (Dugail et al. 2020; Simonnet et al. 2020).

## Data availability

All UK Biobank data used in this work were obtained under Data Access Application 19542 and are available to eligible researchers through the UK Biobank (www.biobank.ac.uk). Gene expression data from the Allen Institute for Brain Science are freely available at https://human.brain-map.org/static/download.

## Code availability

Custom code that supports the findings of this study is available from the corresponding author upon request.

## Author contributions

Conception or design of the study: TJ, BJS, and JF. Manuscript writing and editing: JK, TJ, and DW wrote the manuscript; BJS and JF edited the first draft. All authors critically reviewed the manuscript. Imaging data preprocessing: JK, ZJ, and WC. Visualization: JK, TJ, and CX. Data analysis: JK conducted all the statistical analyses, under the instruction of TJ. Results interpretation: TJ, BJS, and JF. Supervision of the study: TJ and JF. Funding acquisition: TJ and JF.

## Supplementary Material

supplementary_materials_revised_submit_bhac005

## References

[ref1] Amin N, Byrne E, Johnson J, Chenevix-Trench G, Walter S, Nolte IM, kConFab I, Vink JM, Rawal R, Mangino M, et al. Genome-wide association analysis of coffee drinking suggests association with CYP1A1/CYP1A2 and NRCAM. Mol Psychiatry. 2012:17:1116–1129. 21876539 10.1038/mp.2011.101 PMC3482684

[ref2] Babai N, Sendelbeck A, Regus-Leidig H, Fuchs M, Mertins J, Reim K, Brose N, Feigenspan A, Brandstätter JH. Functional roles of Complexin 3 and Complexin 4 at mouse photoreceptor ribbon synapses. J Neurosci. 2016:36:6651–6667. 27335398 10.1523/JNEUROSCI.4335-15.2016 PMC6601749

[ref3] Baron RM, Kenny DA. The moderator–mediator variable distinction in social psychological research: conceptual, strategic, and statistical considerations. J Pers Soc Psychol. 1986:51:1173. 3806354 10.1037//0022-3514.51.6.1173

[ref4] Bulik-Sullivan B, Finucane HK, Anttila V, Gusev A, Day FR, Loh P-R, Duncan L, Perry JR, Patterson N, Robinson EB. An atlas of genetic correlations across human diseases and traits. Nat Genet. 2015a:47:1236. 26414676 10.1038/ng.3406 PMC4797329

[ref5] Bulik-Sullivan BK, Loh P-R, Finucane HK, Ripke S, Yang J, Patterson N, Daly MJ, Price AL, Neale BM, Consortium SWGotPG. LD score regression distinguishes confounding from polygenicity in genome-wide association studies. Nat Genet. 2015b:47:291. 25642630 10.1038/ng.3211 PMC4495769

[ref6] Burgess S, Foley CN, Allara E, Staley JR, Howson JM. A robust and efficient method for Mendelian randomization with hundreds of genetic variants. Nat Commun. 2020:11:1–11. 31953392 10.1038/s41467-019-14156-4 PMC6969055

[ref7] Cornelis MC, Monda KL, Yu K, Paynter N, Azzato EM, Bennett SN, Berndt SI, Boerwinkle E, Chanock S, Chatterjee N. Genome-wide meta-analysis identifies regions on 7p21 (AHR) and 15q24 (CYP1A2) as determinants of habitual caffeine consumption. PLoS Genet. 2011:7:e1002033. 21490707 10.1371/journal.pgen.1002033 PMC3071630

[ref8] Cornelis MC, Byrne EM, Esko T, Nalls MA, Ganna A, Paynter N, Monda KL, Amin N, Fischer K, Renstrom F. Genome-wide meta-analysis identifies six novel loci associated with habitual coffee consumption. Mol Psychiatry. 2015:20:647. 25288136 10.1038/mp.2014.107 PMC4388784

[ref9] Cornelis MC, Kacprowski T, Menni C, Gustafsson S, Pivin E, Adamski J, Artati A, Eap CB, Ehret G, Friedrich N. Genome-wide association study of caffeine metabolites provides new insights to caffeine metabolism and dietary caffeine-consumption behavior. Hum Mol Genet. 2016:25:5472–5482. 27702941 10.1093/hmg/ddw334

[ref10] Croll PH, Voortman T, Ikram MA, Franco OH, Schoufour JD, Bos D, Vernooij MW. Better diet quality relates to larger brain tissue volumes: the Rotterdam Study. Neurology. 2018:90:e2166–e2173. 29769374 10.1212/WNL.0000000000005691

[ref12] Davies NM, Holmes MV, Smith GD. Reading Mendelian randomisation studies: a guide, glossary, and checklist for clinicians. BMJ. 2018:362. 10.1136/bmj.k601 PMC6041728 30002074

[ref13] Dewey KG, Begum K. Long-term consequences of stunting in early life. Matern Child Nutr. 2011:7:5–18. 21929633 10.1111/j.1740-8709.2011.00349.x PMC6860846

[ref14] Dugail I, Amri E-Z, Vitale N. High prevalence for obesity in severe COVID-19: possible links and perspectives towards patient stratification. Biochimie. 2020:S0300-9084(0320):30155–30153. 10.1016/j.biochi.2020.07.001 PMC7340594 32649962

[ref15] Eickhoff SB, Stephan KE, Mohlberg H, Grefkes C, Fink GR, Amunts K, Zilles K. A new SPM toolbox for combining probabilistic cytoarchitectonic maps and functional imaging data. NeuroImage. 2005:25:1325–1335. 15850749 10.1016/j.neuroimage.2004.12.034

[ref16] Emdin CA, Khera AV, Kathiresan S. Mendelian randomization. JAMA. 2017:318:1925–1926. 29164242 10.1001/jama.2017.17219

[ref17] Euesden J, Lewis CM, O’Reilly PF. PRSice: polygenic risk score software. Bioinformatics. 2015:31:1466–1468. 25550326 10.1093/bioinformatics/btu848 PMC4410663

[ref18] Evangelou E, Warren HR, Mosen-Ansorena D, Mifsud B, Pazoki R, Gao H, Ntritsos G, Dimou N, Cabrera CP, Karaman I, et al. Genetic analysis of over 1 million people identifies 535 new loci associated with blood pressure traits. Nat Genet. 2018:50:1412–1425. 30224653 10.1038/s41588-018-0205-x PMC6284793

[ref19] Fjell AM, Grydeland H, Krogsrud SK, Amlien I, Rohani DA, Ferschmann L, Storsve AB, Tamnes CK, Sala-Llonch R, Due-Tønnessen P. Development and aging of cortical thickness correspond to genetic organization patterns. Proc Natl Acad Sci. 2015:112:15462–15467. 26575625 10.1073/pnas.1508831112 PMC4687601

[ref20] Greely H, Sahakian B, Harris J, Kessler RC, Gazzaniga M, Campbell P, Farah MJ. Towards responsible use of cognitive-enhancing drugs by the healthy. Nature. 2008:456:702–705. 19060880 10.1038/456702a

[ref21] Hawrylycz MJ, Lein ES, Guillozet-Bongaarts AL, Shen EH, Ng L, Miller JA, Van De Lagemaat LN, Smith KA, Ebbert A, Riley ZL. An anatomically comprehensive atlas of the adult human brain transcriptome. Nature. 2012:489:391. 22996553 10.1038/nature11405 PMC4243026

[ref22] Jansen IE, Savage JE, Watanabe K, Bryois J, Williams DM, Steinberg S, Sealock J, Karlsson IK, Hägg S, Athanasiu L. Genome-wide meta-analysis identifies new loci and functional pathways influencing Alzheimer’s disease risk. Nat Genet. 2019:51:404–413. 30617256 10.1038/s41588-018-0311-9 PMC6836675

[ref23] Jia T, Chu C, Liu Y, van Dongen J, Papastergios E, Armstrong NJ, Bastin ME, Carrillo-Roa T, den Braber A, Harris M. Epigenome-wide meta-analysis of blood DNA methylation and its association with subcortical volumes: findings from the ENIGMA Epigenetics Working Group. Mol Psychiatry. 2021:26:3884–3895. 31811260 10.1038/s41380-019-0605-z PMC8550939

[ref24] Kanold PO . Subplate neurons: crucial regulators of cortical development and plasticity. Front Neuroanat. 2009:3:16–16. 19738926 10.3389/neuro.05.016.2009 PMC2737439

[ref25] Kanold PO, Shatz CJ. Subplate neurons regulate maturation of cortical inhibition and outcome of ocular dominance plasticity. Neuron. 2006:51:627–638. 16950160 10.1016/j.neuron.2006.07.008

[ref26] Larsson SC, Orsini N. Coffee consumption and risk of dementia and Alzheimer's disease: a dose-response meta-analysis of prospective studies. Nutrients. 2018:10:1501. 10.3390/nu10101501 PMC6213481 30322179

[ref27] Liu M, Jiang Y, Wedow R, Li Y, Brazel DM, Chen F, Datta G, Davila-Velderrain J, McGuire D, Tian C. Association studies of up to 1.2 million individuals yield new insights into the genetic etiology of tobacco and alcohol use. Nat Genet. 2019:51:237. 30643251 10.1038/s41588-018-0307-5 PMC6358542

[ref28] Mortensen LS, Park SJH, Ke J-B, Cooper BH, Zhang L, Imig C, Löwel S, Reim K, Brose N, Demb JB, et al. Complexin 3 increases the fidelity of signaling in a retinal circuit by regulating exocytosis at ribbon synapses. Cell Rep. 2016:15:2239–2250. 27239031 10.1016/j.celrep.2016.05.012 PMC5134263

[ref29] Perlaki G, Orsi G, Kovacs N, Schwarcz A, Pap Z, Kalmar Z, Plozer E, Csatho A, Gabriel R, Komoly S. Coffee consumption may influence hippocampal volume in young women. Brain Imaging Behav. 2011:5:274–284. 21717131 10.1007/s11682-011-9131-6

[ref30] Poole R, Kennedy OJ, Roderick P, Fallowfield JA, Hayes PC, Parkes J. Coffee consumption and health: umbrella review of meta-analyses of multiple health outcomes. BMJ. 2017:359:j5024. 29167102 10.1136/bmj.j5024 PMC5696634

[ref31] Rapp PR, Bachevalier J. Cognitive development and aging. In: Larry RS, Darwin B, Floyd EB, Sascha DL, Anirvan G, Nicholas CS, editors. Fundamental Neuroscience. 4th ed. Academic Press; 2013:919–945. 10.1016/B978-0-12-385870-2.00043-3.

[ref32] Rolls ET, Huang C-C, Lin C-P, Feng J, Joliot M. Automated anatomical labelling atlas 3. NeuroImage. 2020:206:116189. 10.1016/j.neuroimage.2019.116189 31521825

[ref33] Satizabal CL, Adams HH, Hibar DP, White CC, Knol MJ, Stein JL, Scholz M, Sargurupremraj M, Jahanshad N, Roshchupkin GV. Genetic architecture of subcortical brain structures in 38,851 individuals. Nat Genet. 2019:51:1624–1636. 31636452 10.1038/s41588-019-0511-y PMC7055269

[ref34] Simonnet A, Chetboun M, Poissy J, Raverdy V, Noulette J, Duhamel A, Labreuche J, Mathieu D, Pattou F, Jourdain M, et al. High prevalence of obesity in severe acute respiratory syndrome coronavirus-2 (SARS-CoV-2) requiring invasive mechanical ventilation. Obesity (Silver Spring). 2020:28:1195–1199. 32271993 10.1002/oby.22831 PMC7262326

[ref35] Smith SM, Douaud G, Chen W, Hanayik T, Alfaro-Almagro F, Sharp K, Elliott LT. Enhanced brain imaging genetics in UK Biobank. bioRxiv. 2020:2020.2007.2027.223545. 10.1101/2020.07.27.223545.

[ref36] Sudlow C, Gallacher J, Allen N, Beral V, Burton P, Danesh J, Downey P, Elliott P, Green J, Landray M. UK biobank: an open access resource for identifying the causes of a wide range of complex diseases of middle and old age. PLoS Med. 2015:12:e1001779. 25826379 10.1371/journal.pmed.1001779 PMC4380465

[ref37] Sulem P, Gudbjartsson DF, Geller F, Prokopenko I, Feenstra B, Aben KK, Franke B, den Heijer M, Kovacs P, Stumvoll M. Sequence variants at CYP1A1–CYP1A2 and AHR associate with coffee consumption. Hum Mol Genet. 2011:20:2071–2077. 21357676 10.1093/hmg/ddr086 PMC3080612

[ref38] Tamnes CK, Østby Y. Morphometry and development: changes in brain structure from birth to adult age. In: Spalletta G, Piras F, Gili T, editors. Brain Morphometry Neuromethods. New York, NY: Humana Press; 2018:136. 10.1007/978-1-4939-7647-8_10.

[ref39] Truswell A . Cereal grains and coronary heart disease. Eur J Clin Nutr. 2002:56:1–14. 11840174 10.1038/sj.ejcn.1601283

[ref11] van Dam RM, Hu FB, Willett WC. Coffee, caffeine, and health. N Engl J Med. 2020:383:369–378. 32706535 10.1056/NEJMra1816604

[ref40] Viswanathan S, Sheikh A, Looger LL, Kanold PO. Molecularly defined subplate neurons project both to thalamocortical recipient layers and thalamus. Cereb Cortex (New York, NY: 1991). 2017:27:4759–4768. 10.1093/cercor/bhw271 PMC6059176 27655928

[ref41] Watanabe K, Taskesen E, Van Bochoven A, Posthuma D. Functional mapping and annotation of genetic associations with FUMA. Nat Commun. 2017:8:1826. 29184056 10.1038/s41467-017-01261-5 PMC5705698

[ref42] Yu G, Wang L-G, Han Y, He Q-Y. clusterProfiler: an R package for comparing biological themes among gene clusters. Omics. 2012:16:284–287. 22455463 10.1089/omi.2011.0118 PMC3339379

[ref43] Zhao B, Ibrahim JG, Li Y, Li T, Wang Y, Shan Y, Zhu Z, Zhou F, Zhang J, Huang C. Heritability of regional brain volumes in large-scale neuroimaging and genetic studies. Cereb Cortex. 2019:29:2904–2914. 30010813 10.1093/cercor/bhy157 PMC6611460

[ref44] Ziegler G, Dahnke R, Jäncke L, Yotter RA, May A, Gaser C. Brain structural trajectories over the adult lifespan. Hum Brain Mapp. 2012:33:2377–2389. 21898677 10.1002/hbm.21374 PMC6870331

