# Supplementary material for: Increased brain volume from higher cereal and lower coffee intake: shared genetic determinants and impacts on cognition and metabolism
Source: Cereb Cortex. 2022 Feb 7;32(22):5163–74. doi: 10.1093/cercor/bhac005 (PMC9383440; doi:10.1093/cercor/bhac005)
Supplement: supplementary_materials_revised_submit_bhac005 [file supplementary_materials_revised_submit_bhac005.pdf]

Supplementary Information for

Increased brain volume from higher cereal and lower coffee intake: Shared genetic determinants and impacts on cognition and metabolism

Jujiao Kang MSc [1,2,3†], Tianye Jia PhD [2,3,4†*], Zeyu Jiao MSc [1,2,3], Chun Shen PhD [2,3], Chao Xie MSc [2,3], Wei Cheng PhD [2,3], Barbara J Sahakian DSc [2,3,5,6*], David Waxman PhD [2,3], Jianfeng Feng PhD [1,2,3,7*]

Correspondence to: jianfeng64@gmail.com or tianyejia@fudan.edu.cn or bjs1001@medschl.cam.ac.uk

† These authors contributed equally to this work.

This PDF file includes:

Supplementary Materials
Supplementary Methods
Supplementary Results
Supplementary Table 1 to 21

Supplementary Materials

Study participants

Study samples were from the UK Biobank study, a prospective epidemiological study that involves over 500,000 individuals in 22 centres across the UK (Sudlow C et al. 2015). Between 2006 and 2010, participants were recruited to collect a range of questionnaires about detailed phenotypic information including diet, lifestyle, anthropometric and cognitive function assessments, biological samples, including blood and medical records obtained from the NHS registries. Since 2014, a subsample of the original population has been invited back to collect magnetic resonance imaging of body and brain, and questionnaires about diet, lifestyle, and cognitive function assessments.

In the present study, we used data collected at both the baseline and follow-up (i.e. when MRI scans were acquired). The original sample comprised 488289 individuals (56.54 ±8.09 years; 54.21% women), of which 431039 are white British. Further, 810 individuals with Alzheimer's or dementia diagnoses (codes G30/F00 based on ICD-10) and one additional individual with missing Alzheimer's or dementia information were removed. Of the 430228 individuals left over, 336517 individuals with quality-controlled genetic data were used to perform genome-wide association analyses (GWAS). Meanwhile, 18879 individuals with brain MRI data released as the first batch, and the newly- released (Smith SM et al. 2020) 16412 individuals were used as an independent replication. Supplementary Table 1-2 summarised relevant demographic information. Behavioural and neuroimaging data collection and protocol are publicly available on (Sudlow C *et al.* 2015; Miller KL et al. 2016). All participants provided written informed consent to UK Biobank. The UK Biobank study received ethical approval from the NHS National Research Ethics Service

North West (reference number: 16/NW/0274). Data access permission was granted under UKB application 19542 (PI Jianfeng Feng).

Assessment of dietary measurements

Dietary data were obtained from the touchscreen questionnaire at the baseline and the MRI scan appointment. Participants were asked about their frequency of intake of a range of common food and drink items [UK Biobank Category ID: 100052]. The questionnaires were described as below. Cereal intake was obtained from the touchscreen questionnaire. Participants were asked, "How many bowls of cereal do you eat a week?" Either answer < 0 or > 99 was then rejected. If answer > 14, then the participant was asked to confirm. Participants who indicated that they ate cereal at least one bowl of cereal each week were further asked, "What type of cereal do you mainly eat?" The answers included Bran cereal (e.g., All Bran, Branflakes), Biscuit cereal (e.g., Weetabix), Oat cereal (e.g., Ready Brek, porridge), Muesli, and Other (e.g., Cornflakes, Frosties). For coffee intake, participants were asked, "How many cups of coffee do you drink each day? (Include decaffeinated coffee)". Participants who indicated that they drank at least one glass of coffee each day were further asked, "What type of coffee do you usually drink?" The answers included Decaffeinated coffee (any type), Instant coffee, Ground coffee (including espresso, filter, etc.), Other types of coffee. Participants were asked, "How many glasses of water do you drink a day?" for water intake. For tea intake, participants were asked, "How many cups of tea do you drink each day? (Include black and green tea)". Cooked vegetable intake was obtained from the question, "On average how many heaped tablespoons of cooked vegetables would you eat per day? (Do not include potatoes)". Salad / raw vegetable intake assessed using the following question "On average, how many heaped tablespoons of salad or raw vegetables would you eat per day? (Include lettuce, tomato in sandwiches)". Fresh fruit intake was obtained from the question "About how many

pieces of fresh fruit would you eat per day? (Count one apple, one banana, ten grapes, etc. as one piece)". Dried fruit intake was obtained from the question "About how many pieces of dried fruit would you eat per day? (Count one prune, one dried apricot, ten raisins as one piece)". Bread intake was obtained from the question, "How many slices of bread do you eat each week?". For food and drink items above, the answer "less than one" was set to 0.5. Participants were asked about their intake frequency of oil fish/ non-oily fish, processed meat, poultry, beef lamb/mutton, pork, cheese, which was reported as "never", "less than once a week", "once a week", "2-4 times a week", "5-6 times a week", and "Once or more daily" and coded from 0 to 5.

In this study, we excluded individuals beyond four times the standard deviation from the mean for each kind of diet phenotype.

Assessment of lifestyle phenotypes

We included physical activity, sleep, smoking and alcohol as lifestyle phenotypes. Physical activities were assessed using MET (Metabolic Equivalent Task) scores derived based on International Physical Activity Questionnaire) of total physical activity (including walking, moderate, and vigorous activity) and usual walking pace. The time spent watching television was also included to reflect physical activity. Sleep data included information for sleep duration, morningness or eveningness type, insomnia symptoms, daytime dozing, getting up in morning, and nap during day. Smoking status included smoking history and the number of cigarettes currently smoked daily. Alcohol intake was examined using frequency and amounts of alcohol drinking. The detailed description is as follows.

Assessment of physical activity

We used self-reported physical activity measured using a modified version of the International Physical Activity Questionnaire (IPAQ), which assessed total physical activity (TPA), including walking, moderate, and vigorous activity undertaken over the last seven days. MET (Metabolic Equivalent Task) scores data were derived based on IPAQ (International Physical Activity Questionnaire) guidelines. Detailed information referred to the website (http://biobank.ndph.ox.ac.uk/showcase/label.cgi?id=54). We used total Metabolic Equivalent Task (MET) minutes per week for all activity, including walking, moderate, and vigorous activity. Usual walking pace was characterized as "slow pace", "steady average pace", "brisk pace", and coded to 1 to 3. To assess the time spent watching television, participants were asked, "In a typical day, how many hours do you spend watching TV?"

Assessment of sleep

Sleep duration was recorded as the number of reported hours to the following question "About how many hours sleep do you get in every 24 hours? ". Participants were asked "on an average day, how easy do you find getting up in the morning?" with four responses provided ("not at all easy", "not very easy", "fairly easy", "very easy") and coded to 1 to 4. Participants were asked, "Do you consider yourself to be..." to assess morningness or eveningness (chronotype). Four options were provided under this question: "definitely a ‘morning' person", "more a ‘morning' than ‘evening' person", "more an ‘evening' than ‘morning' person", "definitely an ‘evening' person" and coded 1 to 4. To assess insomnia symptoms, participants were asked, "Do you have trouble falling asleep at night, or do you wake up in the middle of the night?" with three options "never/rarely", "sometimes", and "usually" and codes 1,2,3. Participants were asked, "Do you have a nap during the day?" with the same responses to insomnia symptoms. Finally, daytime dozing/sleeping (narcolepsy) was coded based on answers to the following question: "How likely are you to doze

off or fall asleep during the daytime when you don't mean to", with the following options: "never/rarely", "sometimes", "often", "all of the time", coded to 0 to 3.

Assessment of smoking

Smoking data were obtained from the touchscreen questionnaire at the baseline appointment. Participants were asked about their smoking history [UK Biobank Field ID: 20160]. The number of cigarettes currently smoked daily (current cigarette smokers) was acquired from the question "About how many cigarettes do you smoke on average each day?".

Assessment of alcohol intake

Alcohol intake data were obtained from the touchscreen questionnaire at the baseline appointment. Participants were asked about their alcohol intake frequency [UK Biobank Field ID: 1558] was reported as "never", "special occasions only", "one to three times a month", "once or twice a week", "three or four times a week", and "daily or almost daily" and coded from 0 to 5. Average weekly alcohol consumption of a range of drink types: red wine, champagne /white wine, beer/cider, spirits, and fortified wine were collected from participants who indicated they drink alcohol more often than once or twice a week. The measurement units for each of the five alcoholic drink types: measures for spirits, glasses for wines, and pints for beer/cider, which were estimated to be equivalent to 1, 2, and 2.5 units, respectively. Individuals reporting current intake frequency of "one to three times a month", "special occasions only" or "never" (for whom this phenotype was not collected), were assumed to have a weekly alcohol consumption volume of 0. From these measures, we calculated an average intake of alcoholic units per week, derived by combining the self-reported estimated intakes of the different alcoholic beverage consumptions across the five drink types, as in a previous study(Howe LJ et al. 2019).

Assessment of cognitive function

Cognitive function performances were examined at the baseline and the MRI scan appointment. The cognitive tests included fluid intelligence score, reaction time, numeric memory, pairs matching, prospective memory, matrix pattern completion, symbol digit substitution and trail making. The detailed description is as follows.

Fluid intelligence

Fluid intelligence score was obtained from a simple unweighted sum of the number of correct answers given to the 13 fluid intelligence questions. Participants who did not answer all of the questions within the allotted two min limit are scored as zero for each of the unattempted questions.

Reaction time

First, the participants were presented with 2 cards in the middle of the screen. Second, the participants were asked to press the button box as soon as possible when two cards matched. The variable was the mean duration (milliseconds) to the first press of snap-button summed over rounds in which both cards matched.

Numeric memory

Firstly, the participants were presented with a string of numbers on the screen, which subsequently disappeared. Secondly, the participants were asked to enter the number in reverse order. If the answer was correct, there would be a longer number on the screen to remember. The test would end when two mistakes are made or five successive incorrect responses at string length = 2. The variable was maximum string length recalled correctly (range: 0-12), with higher scores indicating better performance.

Pairs matching

Firstly, participants were shown six card-pairs of symbols randomly scrambled on screen. Secondly, cards will then turn face down on the screen, and the participants were asked to select the pairs by touching the screen. As pairs were found correctly, the cards will disappear. The variable was the number of errors made during pairs matching (range 0-146).

Prospective memory

In this test, the participants were asked to remember to carry out a pre-planned instruction after a filled interval. First, at the beginning of the test, they were shown the following instruction: "At the end of the games, we will show you four colored symbols and ask you to touch the blue square. However, to test your memory, we want you to actually touch the orange circle instead." The variable was whether or not the participants remembered to touch the orange circle on the first attempt.

Matrix pattern completion

First, the participants were shown a series of matrix pattern blocks with missing elements. Second, the participants were asked to choose the element that could best complete the pattern from a series of given choices. The variable was the number of puzzles for which the participant gave the correct solution.

Symbol digit substitution

First, the participants were shown a grid linking symbols to a single integer, and another grid containing only symbols. Second, the participants were required to use the symbol-integer pair in the first grid as a key to indicate the number attached to each symbol in the second grid. The variable is the number of symbols correctly matched to digits by the participants.

Tower rearranging

First, the participants were shown illustrations of three piles (towers) on which three iron rings of different colors were placed. Second, the participants were asked to indicate how many steps it takes to rearrange the hoop to another specific position. The variable was the number of puzzles correctly answered.

Trail making

First, the participants were shown 25 circles distributed on the screen, which contained 1-25 numbers (digital trails) or a mixture of numbers and letters (alphanumeric trails). Second, the participants are required to connect the circle from the smallest number to the largest number in one task (number tracking), or alternate between numbers and letters in ascending alphabetical order in another task (alphanumeric tracking). The variable was the duration (deciseconds) to complete the trail. Please note that we reverse the sign of the variable to ensure that the higher the score, the better the performance.

Assessment of body size and blood cholesterol

Body mass index was calculated from the participant's measured weight (kg)/height (m2). Cholesterol, high-density lipoprotein (HDL) cholesterol, low-density lipoprotein (LDL) cholesterol, and triglycerides were measured in the blood sample collected at recruitment.

Assessment of the Alzheimer's disease risk

We used a proxy phenotype for Alzheimer's disease (AD) case-control status derived from the genetic risk index for AD based on parents' diagnoses as suggested in a previous study(Jansen IE et al. 2019). The proxy phenotype ranged approximately from 0 to 2, with values near zero when both parents were unaffected (lower for older parents and possible values below zero if both parents were over age 100) and values of two when both parents were affected.

COVID-19 test

COVID-19 test results data are linked to UK Biobank by Public Health England (PHE). Data were available for the period 16th March 2020 to 3rd August 2020. Data provided included specimen origin (hospital inpatient indicating severe COVID-19 vs. other settings). Detailed information is available on the website (http://biobank.ndph.ox.ac.uk/ukb/exinfo.cgi?src=COVID19_tests). To focus on the COVID-19, we excluded individuals passed away except for those who had positive test results. There were 13145 unique test results available, of which 1649 (12.54%) were positive; 10098 (76.82%) tests were conducted on inpatients; 1069 (639 had available data on BMI and diet) were inpatients and positive.

Structural MRI

Structural MRIs were collected across three imaging centers that were equipped with identical scanners (Siemens Skyra 3T running VD13A SP4 with a Siemens 32-channel RF receive head coil, Munich, Germany). For each scan, Siemens auto-align software determined the field-of-view, which aligns a scout scan to an atlas. If auto-align failed, the radiographer set the alignment. Structural images were acquired with straight sagittal orientation (i.e., with the field of view aligned to the scanner axes), with a resolution of 1 × 1 × 1 mm and a field of view of 208 × 256 × 256 matrix, over a duration of 5 minutes, and with 1-mm isotropic resolution using a 3-dimensional magnetization-prepared rapid-acquisition gradient echo. The MRI protocols have been described in detail elsewhere(Miller KL *et al.* 2016).

Genetic data

Genotype data are available for all 500,000 participants in the UKB cohort. All blood samples were genotyped using the UK BiLEVE array and the UK Biobank axiom array. The two single

nucleotide polymorphism (SNP) arrays are very similar, with over 95% common marker content. Quality control was performed by Affymetrix, the Wellcome Trust Centre for Human Genetics, and by the present authors; this included removal of participants based on missingness, relatedness, gender mismatch, non-British ancestry and other criteria, details of the array design, genotyping, quality control and imputation are available in a previous publication(Bycroft C et al. 2018).

Supplementary Methods

Structural MRI data preprocessing

All structural MRI data were preprocessed in the Statistical Parametric Mapping package(Eickhoff SB et al. 2005) (SPM12) using the VBM8 toolbox with default settings, including the usage of high-dimensional spatial normalisation with an already integrated Dartel template in Montreal Neurological Institute (MNI) space. All images were subjected to nonlinear modulations and corrected for each individual head size. Images were then smoothed with a 6 mm full-width at half-maximum Gaussian kernel with the resulting voxel size 1.5mm^3. The automated anatomical labelling 3 (AAL3) atlas(Rolls ET et al. 2019), which partitioned the brain into 166 regions of interest, was employed to obtain the total brain grey matter volume and region-wise grey matter volume. The majority of discovery samples were assessed in the Cheadle MRI site (84.49%) and the rest in the Newcastle site (15.51%). In comparison, 37.17% of replication samples were assessed in the Cheadle site, 37.87% were tested in the Newcastle site, and the remaining 24.96% were in the Reading site.

Genetic data quality control

Detailed genotyping and quality control procedures of the UK Biobank can be found in http://biobank.ctsu.ox.ac.uk/. In this study, we performed stringent QC standards by PLINK

1.90(Purcell S et al. 2007). Single-nucleotide polymorphisms (SNPs) with call rates <95%, minor allele frequency <0.1%, deviation from the Hardy–Weinberg equilibrium with p<1E-10 were excluded from the analysis. In addition, we selected subjects that were estimated to have recent British ancestry and have no more than ten putative third-degree relatives in the kinship table using the sample quality control information provided by UKB. For more details, we refer to the official document for genetic data of the UKB (http://www.ukbiobank.ac.uk/scientists-3/genetic-data/). After the quality control procedures, we obtained a total of 616,339 SNPs and 336517 participants.

Preprocessing of the Allen Human Brain Atlas data

We used the transcriptomic data from six neurotypical adult brains in the Allen Human Brain Atlas (Hawrylycz MJ et al. 2012). Standard preprocessing steps included probe-to-gene re-annotation, intensity-based data filtering, probe selection by mean, separating tissue samples into subcortical and cortical regions, and within-donor normalization, as reported previously (Arnatkevičiūtė A et al. 2019; Shen C et al. 2020). In the next step, we separated the samples into the areas based on their MNI coordinates, using the automated anatomical labelling 3 (AAL3) atlas (Rolls ET *et al.* 2019) and excluding the samples located outside of the grey matter defined by this atlas. To control for the inter-individual differences, we conducted two within-donor normalisations. The expression data were first normalised within-sample and across-gene and then normalised across samples. One gene failed the normalisation and therefore was deleted, resulting in 15,408 genes. We used the mean expression of samples located in the brain region and the mean expression in the brain region of all subjects as the gene expression in each brain region defined by AAL3 atlas (Rolls ET *et al.* 2019).

Association analysis

Generalized linear model was used to test the pairwise associations between diet measurements and total/regional grey matter volume (GMV). To ensure that the following variables did not influence the results, they were used in the model as covariates of no interest: age at MRI scan, gender, imaging sites (dummy variable), and total intracranial volume (TIV). The pairwise associations between the SNPs and diets measurements, lifestyle phenotypes, and body/blood fat levels were examined with baseline age, gender and the top 40 genetic principal components as covariates. We also examine the associations between the cereal/coffee intakes and diets measurements, lifestyle phenotypes, and body/blood fat levels, adjusting for baseline age and gender. A t-statistic and the correlation coefficient were obtained for each regression model. Finally, Bonferroni corrections to correct the results for multiple comparisons were performed.

Genome-wide association analysis and annotation of significant variants.

We performed genome-wide association analysis (GWAS) of cereal/coffee intake using PLINK 1.90 (Purcell S *et al.* 2007). The covariates include were age, gender, and the top 40 ancestry principal components. FUMA (Watanabe K et al. 2017) online platform (version 1.3.6, http://fuma.ctglab.nl/) was employed to identify independent lead variants of cereal/coffee intake. FUMA first identifies significant variants with P value less than 5E-8 that were largely independent of each other ($r^2 < 0.6$). Based on the clumping of the independent significant variants ($r^2 < 0.1$), independent lead variants were obtained. Further details can be found in previous study (Watanabe K *et al.* 2017).

The independent significant SNPs were mapped to genes based on positional, eQTL and chromatin interaction mapping using FUMA (Watanabe K *et al.* 2017). Positional mapping map SNPs to genes based on physical distances (within a 10-kb window). The eQTL mapping map SNPs to genes based on eQTL associations that SNP was significant (false discovery rate (FDR)≤ 0.05)

expression level of gene using information on eQTLs of 49 tissue type in GTEX (Consortium G 2015) v8 and BRAINEAC (Ramasamy A et al. 2014). The eQTL mapping was based on cis-eQTLs and could map SNPs to genes up to 1Mb apart. The chromatin interaction mapping map SNPs to genes using Hi-C data of 14 tissue types from GSE87112(Schmitt AD et al. 2016).

Shared lead SNPs of cereal and coffee were mapped to genes based on cis-eQTL (p value≤ 0.05) in 13 brain regions using database GTEX (Consortium G 2015) v8 with FUMA (Watanabe K *et al.* 2017). We used all brain tissues available in GTEx(Consortium G 2015) v8, including regions such as the amygdala, anterior cingulate cortex (BA24), caudate, cerebellar hemisphere, cerebellum, cortex, frontal cortex (BA9), hippocampus, hypothalamus, nucleus accumbens, putamen, spinal cord cervical and substantia nigra. The eQTL mapping assigned SNPs to genes up to 1Mb apart.

Heritability and genetic correlation estimation

The LDSC software (https://github.com/bulik/ldsc) was employed to estimate the heritability of cereal intake and coffee intake as well as their genome-wide genetic correlation (Bulik-Sullivan B et al. 2015). We used the pre-calculated LD scores using 1000 Genomes European data. We used the overlap of summary statistics variants and HapMap variants as recommended (Bulik-Sullivan B *et al.* 2015).

Mendelian Randomisation using the inverse-variance weighted method

Using the availability of neuroimaging data as a random stratification of UK Biobank data, we reconducted GWAS of cereal/coffee intake in the large discovery sample without neuroimaging information (N=308839), as well as conducting a new GWAS of TGMV in full neuroimaging data (N= 27678). Using the TwoSampleMR R package (https://github.com/MRCIEU/TwoSampleMR),

a two-sample Mendelian randomisation was performed to estimate the effect of cereal/coffee intake on the TGMV and vice versa. SNPs with P value < 5E−08 and r2<0.001 in a 1000-kb window size were identified as genetic instruments. Effects of each SNP were combined using the inverse-variance weighted meta-analysis, where the resulting estimate represents the slope of a weighted regression of SNP-outcome effects on SNP-exposure effects, where the intercept is constrained to zero (Davies NM et al. 2018).

Mediation analysis

Mediation effects were examined using Baron and Kenny's (1986) (Baron RM and DA Kenny 1986) causal steps approach. The causal steps approach involved four steps to establishing mediation. Firstly, a significant relation of the independent variable to the dependent variable is required in $Y = k_1 + \tau X + \varepsilon_1$ ($reject\ H_0: \tau = 0$). Secondly, a significant relation of the independent variable to the hypothesised mediating variable is required in $Z = k_2 + \alpha X + \varepsilon_2$ ($reject\ H_0: \alpha = 0$). Thirdly, the mediating variable must be significantly related to the dependent variable when both the independent variable and mediating variable are predictors of the dependent variable in $Y = k_3 + \tau' X + \beta Z + \varepsilon_3$ ($reject\ H_0: \beta = 0$). Fourthly, the coefficient relating the independent variable to the dependent variable must be larger (in absolute value) than the coefficient relating the independent variable to the dependent variable in the regression model with both the independent variable and the mediating variable predicting the dependent variable ($i.e. |\tau| > |\tau'|$). To further evaluate the p-value of the significant mediation identified by the above process, we performed 1000 times bootstrap of the individuals to obtain the distribution of the proportion of the mediation, i.e., $PM = (\tau - \tau')/\tau$, under the alternative hypothesis. Thus, the PM was expected to be positive by definition, and the corresponding p-value could be calculated as the doubled chance of observing the PM less than zero during the 1000 bootstrap procedure. As

no priory assumption about whether diet or lifestyle/ blood and body fat levels should serve as the mediator for their associations with the lead SNPs, we, therefore, identified the most likely mediator with an excess PM, i.e., the model showing higher PM, of which the significance level was again evaluated through a 1000-bootstrap process.

Functional enrichment analysis

Functional properties of these genes were characterized by gene ontology (GO) terms including molecular function (MF), cellular component (CC), and biological process (BP) using "clusterProfiler" (Yu G et al. 2012) R package. Adjusted p values were acquired using Benjamini-Hochberg method.

Supplementary Results

Mendelian Randomisation using the inverse-variance weighted method

To further validate the results of the modified Mendelian randomisation method, we combined Mendelian randomisation estimates from each genetic instrument using the inverse-variance weighted (IVW) meta-analysis. The new results came to the same conclusion regarding a harmful causal effect of coffee intake on the TGMV (β=-5.420; se=1.649; P=1.01E-03). Further, no causal relationship was observed for TGMV on cereal intake (β =0.004; se=0.003 P =2.23E-01) or coffee intake (β =-0.0003; se=0.001 P = 8.13E-01) through IVW. However, the IVW approach now additionally suggests a protective relationship between cereal intake and TGMV (β=6.258; se=1.912; P=1.06E-03), while our PRS approach suggested an inconclusive relationship. We note that it is generally challenging for the IVW approach to distinguish the real causal influence from genetic pleiotropy and avoid the risk of reverse causality (Davies NM *et al.* 2018), which is mainly due to the lack of stringent selection criteria for instrumental variables. The modified Mendelian

randomisation employed in our study, however, obtained valid instrumental variables by gradually removing SNPs cross-associating with both independent and dependent variables at various thresholds (up to P<0.5), thus properly addressing both pleiotropy and reverse causal inference issues.

References

Arnatkevičiūtė A, Fulcher BD, Fornito A. 2019. A practical guide to linking brain-wide gene expression and neuroimaging data. Neuroimage. 189:353-367.

Baron RM, Kenny DA. 1986. The moderator–mediator variable distinction in social psychological research: Conceptual, strategic, and statistical considerations. Journal of personality and social psychology. 51:1173.

Bulik-Sullivan B, Finucane HK, Anttila V, Gusev A, Day FR, Loh P-R, Duncan L, Perry JR, Patterson N, Robinson EB. 2015. An atlas of genetic correlations across human diseases and traits. Nature genetics. 47:1236.

Bycroft C, Freeman C, Petkova D, Band G, Elliott LT, Sharp K, Motyer A, Vukcevic D, Delaneau O, O'Connell J. 2018. The UK Biobank resource with deep phenotyping and genomic data. Nature. 562:203.

Consortium G. 2015. The Genotype-Tissue Expression (GTEx) pilot analysis: multitissue gene regulation in humans. Science. 348:648-660.

Davies NM, Holmes MV, Smith GD. 2018. Reading Mendelian randomisation studies: a guide, glossary, and checklist for clinicians. Bmj. 362.

Eickhoff SB, Stephan KE, Mohlberg H, Grefkes C, Fink GR, Amunts K, Zilles K. 2005. A new SPM toolbox for combining probabilistic cytoarchitectonic maps and functional imaging data. Neuroimage. 25:1325-1335.

Hawrylycz MJ, Lein ES, Guillozet-Bongaarts AL, Shen EH, Ng L, Miller JA, van de Lagemaat LN, Smith KA, Ebbert A, Riley ZL, Abajian C, Beckmann CF, Bernard A, Bertagnolli D, Boe AF, Cartagena PM, Chakravarty MM, Chapin M, Chong J, Dalley RA, Daly BD, Dang C, Datta S, Dee N, Dolbeare TA, Faber V, Feng D, Fowler DR, Goldy J, Gregor BW, Haradon Z, Haynor DR, Hohmann JG, Horvath S, Howard RE, Jeromin A, Jochim JM, Kinnunen M, Lau C, Lazarz ET, Lee C, Lemon TA, Li L, Li Y, Morris JA, Overly CC, Parker PD, Parry SE, Reding M, Royall JJ, Schulkin J, Sequeira PA, Slaughterbeck CR, Smith SC, Sodt AJ, Sunkin SM, Swanson BE, Vawter MP, Williams D, Wohnoutka P, Zielke HR, Geschwind DH, Hof PR, Smith SM, Koch C, Grant SGN, Jones AR. 2012. An anatomically comprehensive atlas of the adult human brain transcriptome. Nature. 489:391-399.

Howe LJ, Lawson DJ, Davies NM, Pourcain BS, Lewis SJ, Smith GD, Hemani G. 2019. Genetic evidence for assortative mating on alcohol consumption in the UK Biobank. Nature communications. 10:1-10.

Jansen IE, Savage JE, Watanabe K, Bryois J, Williams DM, Steinberg S, Sealock J, Karlsson IK, Hägg S, Athanasiu L. 2019. Genome-wide meta-analysis identifies new loci and functional pathways influencing Alzheimer's disease risk. Nature genetics. 51:404-413.

Miller KL, Alfaro-Almagro F, Bangerter NK, Thomas DL, Yacoub E, Xu J, Bartsch AJ, Jbabdi S, Sotiropoulos SN, Andersson JL. 2016. Multimodal population brain imaging in the UK Biobank prospective epidemiological study. Nature neuroscience. 19:1523.

Purcell S, Neale B, Todd-Brown K, Thomas L, Ferreira MA, Bender D, Maller J, Sklar P, De Bakker PI, Daly MJ. 2007. PLINK: a tool set for whole-genome association and population-based linkage analyses. The American journal of human genetics. 81:559-575.

Ramasamy A, Trabzuni D, Guelfi S, Varghese V, Smith C, Walker R, De T, Coin L, De Silva R, Cookson MR. 2014. Genetic variability in the regulation of gene expression in ten regions of the human brain. Nature neuroscience. 17:1418-1428.

Rolls ET, Huang C-C, Lin C-P, Feng J, Joliot M. 2019. Automated anatomical labelling atlas 3. NeuroImage.116189.

Schmitt AD, Hu M, Jung I, Xu Z, Qiu Y, Tan CL, Li Y, Lin S, Lin Y, Barr CL. 2016. A compendium of chromatin contact maps reveals spatially active regions in the human genome. Cell reports. 17:2042-2059.

Shen C, Luo Q, Chamberlain SR, Morgan S, Romero-Garcia R, Du J, Zhao X, Touchette É, Montplaisir J, Vitaro F. 2020. What is the link between attention-deficit/hyperactivity disorder and sleep disturbance? A multimodal examination of longitudinal relationships and brain structure using large-scale population-based cohorts. Biol Psychiatry. 88:459-469.

Smith SM, Douaud G, Chen W, Hanayik T, Alfaro-Almagro F, Sharp K, Elliott LT. 2020. Enhanced Brain Imaging Genetics in UK Biobank. bioRxiv.2020.2007.2027.223545.

Sudlow C, Gallacher J, Allen N, Beral V, Burton P, Danesh J, Downey P, Elliott P, Green J, Landray M. 2015. UK biobank: an open access resource for identifying the causes of a wide range of complex diseases of middle and old age. PLoS medicine. 12:e1001779.

Watanabe K, Taskesen E, Van Bochoven A, Posthuma D. 2017. Functional mapping and annotation of genetic associations with FUMA. Nature communications. 8:1826.

Yu G, Wang L-G, Han Y, He Q-Y. 2012. clusterProfiler: an R package for comparing biological themes among gene clusters. Omics: a journal of integrative biology. 16:284-287.

Index of Supplementary Tables 1–21 (ordered by appearance in the main text)

Table	Contents
Supplementary Table 1	Baseline characteristics of study samples in the UK Biobank cohort.
Supplementary Table 2	Follow-up characteristics of study samples in the UK Biobank cohort.
Supplementary Table 3	Associations between total brain grey matter volume and diet phenotypes.
Supplementary Table 4	Confirmation of the association between TGMV and cereal/coffee intake.
Supplementary Table 5	Genome-wide independent significant variants of the cereal intake.
Supplementary Table 6	Genome-wide independent significant variants of the coffee intake.
Supplementary Table 7	Associations between the share lead variants and diet measurements.
Supplementary Table 8	Results of the modified mendelian randomization analysis.
Supplementary Table 9	GO terms for genes.
Supplementary Table 10	Associations between the share lead variants and lifestyle phenotypes.
Supplementary Table 11	Associations between the cereal/coffee intake and lifestyle phenotypes.
Supplementary Table 12	Significant mediation models of SNPs, lifestyle phenotypes, and the intake of cereal and coffee.
Supplementary Table 13	Associations between the shared lead variants and body/blood fat levels.
Supplementary Table 14	Associations between the cereal/coffee intake and body/blood fat levels.
Supplementary Table 15	Significant mediation models of SNPs, body/blood fat levels and the intake of cereal and coffee.
Supplementary Table 16	Associations between the GMV-association patterns of cognitive functions and cereal/coffee intake.
Supplementary Table 17	Validation of the associations between the GMV-association patterns of cognitive functions and cereal/coffee intake.
Supplementary Table 18	Associations between the GMV-association patterns of the cereal/coffee intake and the spatial expression of genes.
Supplementary Table 19	Association between the GMV-association patterns of the cognitive functions and the expression pattern of the CPLX3 gene.
Supplementary Table 20	Validation of associations between the gene-expression pattern of CPLX3 gene and the GMV-association patterns of phenotypes.
Supplementary Table 21	The GMV-association patterns of the follow-up cereal/coffee intake and cognitive functions in the discovery sample.

Supplementary Table 1. Baseline characteristics of study samples in the UK Biobank cohort.

	Discovery sample			Replication sample			T test		
	N=18879			N=16412					
N. of females (%)	9890(52.39%)			8655(52.74%)					
	N	mean	std	N	mean	std	Cohen's D	T	P
Age at baseline (years)	18879	55.10	7.46	16412	54.90	7.37	0.026	2.45	1.44E-02
Diet									
Cereal intake	18858	4.85	2.66	16395	4.78	2.69	0.027	2.52	1.19E-02
Coffee intake	18840	2.07	1.92	16314	2.00	1.80	0.038	3.53	4.14E-04
Water intake	18756	2.42	1.98	16317	2.56	2.00	-0.069	-6.45	1.17E-10
Tea intake	18781	3.40	2.54	16338	3.30	2.47	0.042	3.93	8.48E-05
Cooked vegetable intake	18667	2.55	1.33	16238	2.56	1.33	-0.011	-0.98	3.27E-01
Salad / raw vegetable intake	18707	2.06	1.69	16184	2.02	1.53	0.028	2.58	9.82E-03
Fresh fruit intake	18767	2.17	1.34	16323	2.20	1.34	-0.021	-1.94	5.27E-02
Dried fruit intake	18651	0.80	1.20	16191	0.80	1.20	-0.001	-0.11	9.10E-01
Bread intake	18605	12.25	8.05	16249	11.92	7.82	0.041	3.85	1.19E-04
Oily fish intake	18848	1.64	0.89	16389	1.64	0.89	-0.001	-0.07	9.41E-01
Non-oily fish intake	18843	1.78	0.77	16375	1.77	0.76	0.014	1.27	2.05E-01
Processed meat intake	18871	1.86	1.05	16402	1.84	1.05	0.017	1.57	1.18E-01
Poultry intake	18869	2.29	0.89	16404	2.29	0.90	0.002	0.17	8.65E-01
Beef intake	18862	1.43	0.82	16398	1.45	0.82	-0.018	-1.67	9.45E-02
Lamb/mutton intake	18834	1.08	0.67	16383	1.09	0.68	-0.011	-1.02	3.08E-01
Pork intake	18833	1.12	0.68	16377	1.11	0.67	0.016	1.48	1.40E-01
Cheese intake	18596	2.62	1.04	16126	2.68	1.05	-0.054	-4.99	6.20E-07
Lifestyle									
Time spent watching television (TV)	18825	2.46	1.44	16373	2.35	1.40	0.073	6.80	1.09E-11
Usual walking pace	18846	2.47	0.56	16392	2.49	0.55	-0.033	-3.07	2.13E-03
Summed MET minutes per week for all activity	16170	2475.16	2454.39	14163	2445.76	2383.88	0.012	1.05	2.92E-01
MET minutes per week for moderate activity	16170	836.99	1124.67	14163	820.33	1107.09	0.015	1.30	1.95E-01
MET minutes per week for vigorous activity	16170	672.17	1068.91	14163	668.95	1036.28	0.003	0.27	7.91E-01
MET minutes per week for walking	16170	966.00	1033.92	14163	956.47	1009.89	0.009	0.81	4.19E-01
Sleep duration	18833	7.17	0.96	16388	7.18	0.95	-0.012	-1.13	2.57E-01
Getting up in morning	18765	3.10	0.77	16360	3.10	0.75	0.006	0.56	5.72E-01
Morning/evening person (chronotype)	16803	2.23	0.92	14631	2.23	0.93	-0.006	-0.52	6.05E-01
Nap during day	18879	1.43	0.57	16408	1.41	0.56	0.029	2.75	5.93E-03
Sleeplessness / insomnia	18871	1.99	0.72	16403	1.96	0.73	0.046	4.30	1.71E-05
Daytime dozing / sleeping (narcolepsy)	18859	0.22	0.46	16393	0.22	0.46	0.002	0.16	8.69E-01
Ever smoked	18840	0.57	0.49	16390	0.56	0.50	0.018	1.73	8.44E-02
Number of cigarettes currently smoked daily (current cigarette smokers)	687	14.64	8.83	542	13.93	7.93	0.084	1.47	1.42E-01
Alcohol intake frequency.	18877	3.34	1.36	16409	3.37	1.37	-0.021	-1.99	4.70E-02
Average weekly alcohol intake	18768	17.18	18.05	16310	17.37	17.87	-0.010	-0.97	3.31E-01
Body/blood fat									
Body mass index (BMI)	18879	26.65	4.27	16412	26.49	4.16	0.037	3.45	5.52E-04
Cholesterol	17857	5.73	1.10	15703	5.75	1.07	-0.018	-1.64	1.01E-01
HDL cholesterol	16269	1.47	0.37	14330	1.49	0.38	-0.040	-3.50	4.59E-04
LDL direct	17820	3.58	0.84	15671	3.59	0.82	-0.015	-1.36	1.73E-01
Triglycerides	17843	1.66	0.98	15691	1.63	0.94	0.037	3.41	6.49E-04
Cognitive function									
Fluid intelligence score	6745	6.75	1.98	4999	6.87	2.03	-0.060	-3.23	1.24E-03
Maximum digits remembered correctly	1868	7.00	1.21	1737	6.98	1.25	0.014	0.43	6.67E-01
Prospective memory result	6791	0.88	0.33	5020	0.88	0.32	-0.014	-0.77	4.44E-01
Mean time to correctly identify matches	18849	538.20	100.14	16383	533.91	97.81	0.043	4.05	5.06E-05
Number of incorrect matches in round	18778	0.36	0.86	16367	0.37	0.90	-0.012	-1.10	2.73E-01

Notes: The table reports the baseline characteristics of the discovery and replication sample respectively. N refers to the number of non-missing samples of a specific phenotype. Mean and std represent the mean and standard deviation of the phenotype. Cohen's D, T and P refers to the effect size, T statistic and two-side p value respectively, calculated from the t-test between two groups.

Supplementary Table 2. Follow-up characteristics of study samples in the UK Biobank cohort.

	Discovery sample			Replication sample			T test		
N. of females (%)	N=18879<br>9890(52.39%)			N=16412<br>8655(52.74%)					
	N	mean	std	N	mean	std	Cohen's D	T	P
Age at MRI scan (years)	18879	63.24	7.45	16412	64.98	7.40	-0.234	-21.94	6.12E-106
Diet									
Cereal intake	18727	4.58	2.78	16311	4.42	2.84	0.058	5.45	5.11E-08
Coffee intake	18657	2.03	1.75	16251	2.00	1.67	0.018	1.64	1.01E-01
Water intake	18643	2.38	1.89	16227	2.61	1.93	-0.120	-11.16	7.29E-29
Tea intake	18678	3.36	2.47	16220	3.15	2.33	0.086	8.03	1.02E-15
Cooked vegetable intake	18587	2.72	1.49	16077	2.72	1.42	0.003	0.31	7.57E-01
Salad / raw vegetable intake	18555	2.12	1.71	16171	2.23	1.73	-0.063	-5.87	4.30E-09
Fresh fruit intake	18647	2.19	1.39	16243	2.25	1.40	-0.041	-3.78	1.59E-04
Dried fruit intake	18524	0.79	1.20	16082	0.81	1.21	-0.018	-1.63	1.04E-01
Bread intake	18613	10.26	7.10	16187	9.67	6.68	0.084	7.85	4.30E-15
Oily fish intake	18731	1.77	0.91	16304	1.77	0.93	-0.004	-0.38	7.04E-01
Non-oily fish intake	18706	1.78	0.76	16288	1.78	0.77	0.002	0.21	8.34E-01
Processed meat intake	18739	1.83	1.04	16314	1.77	1.06	0.053	4.92	8.59E-07
Poultry intake	18744	2.34	0.92	16317	2.29	0.94	0.052	4.87	1.13E-06
Beef intake	18725	1.38	0.80	16299	1.35	0.80	0.046	4.33	1.51E-05
Lamb/mutton intake	18715	1.00	0.63	16283	0.95	0.64	0.065	6.04	1.59E-09
Pork intake	18703	1.09	0.68	16276	1.03	0.67	0.081	7.59	3.17E-14
Cheese intake	18416	2.66	1.07	15980	2.78	1.08	-0.109	-10.12	4.89E-24
Lifestyle									
Time spent watching television (TV)	18713	2.83	1.53	16284	2.78	1.53	0.031	2.87	4.05E-03
Usual walking pace	18723	2.42	0.57	16304	2.41	0.58	0.030	2.83	4.61E-03
Sleep duration	18710	7.16	1.04	16280	7.15	1.02	0.005	0.43	6.70E-01
Getting up in morning	18745	3.22	0.73	16313	3.26	0.73	-0.053	-4.92	8.76E-07
Morning/evening person (chronotype)	17072	2.16	0.94	14882	2.15	0.94	0.010	0.88	3.77E-01
Nap during day	18750	1.48	0.60	16321	1.49	0.60	-0.025	-2.37	1.78E-02
Sleeplessness / insomnia	18743	2.09	0.72	16308	2.12	0.73	-0.035	-3.31	9.49E-04
Daytime dozing / sleeping (narcolepsy)	18730	0.25	0.48	16307	0.25	0.49	-0.015	-1.41	1.59E-01
Ever smoked	18696	0.54	0.50	16270	0.53	0.50	0.022	2.05	4.00E-02
Number of cigarettes currently smoked daily (current cigarette smokers)	377	12.62	7.48	255	13.18	8.57	-0.071	-0.88	3.81E-01
Alcohol intake frequency.	18750	3.13	1.40	16313	3.14	1.42	-0.004	-0.38	7.06E-01
Average weekly alcohol intake	18649	14.65	16.69	16219	14.36	16.36	0.018	1.64	1.01E-01
Cognitive function									
Fluid intelligence score	17045	6.80	2.06	15536	6.57	2.03	0.116	10.43	2.00E-25
Maximum digits remembered correctly	8184	6.80	1.24	15355	6.78	1.26	0.019	1.38	1.68E-01
Prospective memory result	17459	0.87	0.33	15696	0.82	0.38	0.140	12.71	6.51E-37
Mean time to correctly identify matches	17411	587.69	106.70	15595	598.72	109.33	-0.102	-9.27	1.96E-20
Number of incorrect matches in round	17476	0.33	0.83	15698	0.35	0.86	-0.024	-2.19	2.84E-02
Number of puzzles correctly solved	7564	8.07	2.10	15378	8.00	2.12	0.032	2.25	2.42E-02
Number of symbol digit matches made correctly	7584	19.35	5.21	15385	18.83	5.24	0.099	7.07	1.64E-12
Number of puzzles correct	7495	10.09	3.22	15264	9.93	3.21	0.051	3.59	3.34E-04
Duration to complete numeric path	7647	217.10	83.23	15520	224.39	86.74	-0.085	-6.10	1.10E-09
Duration to complete alphanumeric path	7647	531.45	255.91	15520	555.02	273.04	-0.088	-6.31	2.91E-10

Notes: The table reports the follow-up characteristics of the discovery and replication sample respectively. N refers to the number of non-missing samples of a specific phenotype. Mean and std represent the mean and standard deviation of the phenotype. Cohen's D, T and P refers to the effect size, T statistic and two-side p value respectively, calculated from the t-test between two groups.

Supplementary Table 3. Associations between total brain grey matter volume and diet phenotypes.

Panel A. Correlations between TGMV and follow-up diet measurements.

Diet	beta	95%CI_L	95%CI_H	se	t	r	p	df.e
Cereal intake	0.976	0.816	1.137	0.082	11.916	0.087	1.27E-32	18721
Coffee intake	-1.148	-1.403	-0.894	0.130	-8.849	-0.065	9.65E-19	18651
Tea intake	0.218	0.038	0.399	0.092	2.376	0.017	1.75E-02	18672
Water intake	-0.542	-0.781	-0.304	0.122	-4.453	-0.033	8.52E-06	18637
Cooked vegetable intake	-0.301	-0.601	-0.001	0.153	-1.965	-0.014	4.94E-02	18581
Salad / raw vegetable intake	-0.213	-0.475	0.049	0.134	-1.597	-0.012	1.10E-01	18549
Fresh fruit intake	0.472	0.150	0.793	0.164	2.872	0.021	4.09E-03	18641
Dried fruit intake	1.182	0.807	1.558	0.192	6.170	0.045	6.97E-10	18518
Bread intake	-0.015	-0.080	0.051	0.034	-0.440	-0.003	6.60E-01	18607
Oily fish intake	-0.012	-0.508	0.483	0.253	-0.048	0.000	9.62E-01	18725
Non-oily fish intake	0.118	-0.464	0.699	0.297	0.397	0.003	6.91E-01	18700
Processed meat intake	-1.737	-2.179	-1.296	0.225	-7.712	-0.056	1.30E-14	18733
Poultry intake	-0.685	-1.168	-0.202	0.246	-2.782	-0.020	5.41E-03	18738
Beef intake	-1.416	-1.974	-0.859	0.285	-4.977	-0.036	6.51E-07	18719
Lamb/mutton intake	-2.093	-2.802	-1.383	0.362	-5.783	-0.042	7.45E-09	18709
Pork intake	-1.996	-2.652	-1.339	0.335	-5.960	-0.044	2.57E-09	18697
Cheese intake	-0.166	-0.587	0.254	0.215	-0.775	-0.006	4.39E-01	18410

Panel B. Correlations between TGMV and baseline diet measurements.

Diet	beta	95%CI_L	95%CI_H	se	t	r	p	df.e
Cereal intake	0.913	0.746	1.080	0.085	10.728	0.078	8.97E-27	18852
Coffee intake	-1.041	-1.273	-0.810	0.118	-8.834	-0.064	1.09E-18	18834
Tea intake	0.053	-0.121	0.228	0.089	0.598	0.004	5.50E-01	18775
Water intake	-0.395	-0.623	-0.167	0.116	-3.400	-0.025	6.74E-04	18750
Cooked vegetable intake	-0.519	-0.855	-0.183	0.171	-3.028	-0.022	2.46E-03	18661
Salad / raw vegetable intake	-0.231	-0.496	0.034	0.135	-1.709	-0.012	8.75E-02	18701
Fresh fruit intake	0.142	-0.192	0.477	0.171	0.835	0.006	4.04E-01	18761
Dried fruit intake	0.935	0.559	1.311	0.192	4.872	0.036	1.11E-06	18645
Bread intake	0.029	-0.029	0.087	0.030	0.979	0.007	3.28E-01	18599
Oily fish intake	-0.526	-1.031	-0.020	0.258	-2.038	-0.015	4.16E-02	18842
Non-oily fish intake	-0.380	-0.958	0.199	0.295	-1.287	-0.009	1.98E-01	18837
Processed meat intake	-1.079	-1.520	-0.637	0.225	-4.787	-0.035	1.71E-06	18865
Poultry intake	-0.501	-0.996	-0.005	0.253	-1.980	-0.014	4.77E-02	18863
Beef intake	-1.376	-1.918	-0.833	0.277	-4.966	-0.036	6.88E-07	18856
Lamb/mutton intake	-1.855	-2.525	-1.185	0.342	-5.428	-0.040	5.78E-08	18828
Pork intake	-1.583	-2.241	-0.926	0.335	-4.722	-0.034	2.35E-06	18827
Cheese intake	0.001	-0.428	0.429	0.219	0.003	0.000	9.98E-01	18590

Panel C. Correlations between TGMV and baseline dietary measurements, adjusting for the follow-up diets.

Diet	beta	95%CI_L	95%CI_H	se	t	r	p	df.e
Cereal intake	0.513	0.312	0.714	0.102	5.005	0.037	5.63E-07	18704
Coffee intake	-0.551	-0.881	-0.220	0.169	-3.268	-0.024	1.08E-03	18620
Tea intake	-0.189	-0.443	0.065	0.129	-1.461	-0.011	1.44E-01	18590
Water intake	-0.124	-0.418	0.170	0.150	-0.828	-0.006	4.08E-01	18531
Cooked vegetable intake	-0.415	-0.796	-0.033	0.195	-2.129	-0.016	3.33E-02	18391
Salad / raw vegetable intake	-0.183	-0.482	0.116	0.153	-1.198	-0.009	2.31E-01	18404
Fresh fruit intake	-0.094	-0.494	0.306	0.204	-0.461	-0.003	6.45E-01	18540
Dried fruit intake	0.551	0.136	0.967	0.212	2.602	0.019	9.28E-03	18314

Bread intake	0.057	-0.012	0.126	0.035	1.611	0.012	1.07E-01	18371
Oily fish intake	-0.803	-1.431	-0.175	0.320	-2.505	-0.018	1.23E-02	18696
Non-oily fish intake	-0.538	-1.203	0.126	0.339	-1.588	-0.012	1.12E-01	18667
Processed meat intake	-0.200	-0.729	0.330	0.270	-0.738	-0.005	4.60E-01	18724
Poultry intake	-0.133	-0.796	0.529	0.338	-0.395	-0.003	6.93E-01	18727
Beef intake	-0.948	-1.617	-0.279	0.341	-2.777	-0.020	5.49E-03	18702
Lamb/mutton intake	-1.153	-1.978	-0.329	0.421	-2.741	-0.020	6.12E-03	18664
Pork intake	-0.791	-1.571	-0.010	0.398	-1.986	-0.015	4.70E-02	18656
Cheese intake	0.216	-0.310	0.742	0.268	0.804	0.006	4.21E-01	18245

Panel D. Correlations between TGMV and follow-up dietary measurements, adjusting for socioeconomic factors.

Diet	beta	95%CI_L	95%CI_H	se	t	r	p	df.e
Cereal intake	1.001	0.832	1.170	0.086	11.597	0.089	5.57E-31	16857
Coffee intake	-1.136	-1.403	-0.869	0.136	-8.330	-0.064	8.69E-17	16798
Tea intake	0.202	0.011	0.393	0.097	2.078	0.016	3.77E-02	16815
Water intake	-0.452	-0.704	-0.201	0.129	-3.520	-0.027	4.32E-04	16794
Cooked vegetable intake	-0.282	-0.599	0.034	0.161	-1.748	-0.014	8.05E-02	16746
Salad / raw vegetable intake	-0.220	-0.496	0.057	0.141	-1.559	-0.012	1.19E-01	16725
Fresh fruit intake	0.453	0.113	0.793	0.173	2.610	0.020	9.06E-03	16793
Dried fruit intake	1.173	0.774	1.573	0.204	5.756	0.045	8.76E-09	16688
Bread intake	-0.004	-0.074	0.065	0.035	-0.127	-0.001	8.99E-01	16766
Oily fish intake	-0.154	-0.677	0.370	0.267	-0.576	-0.004	5.64E-01	16863
Non-oily fish intake	0.146	-0.468	0.761	0.314	0.467	0.004	6.41E-01	16844
Processed meat intake	-1.828	-2.293	-1.363	0.237	-7.702	-0.059	1.42E-14	16869
Poultry intake	-0.737	-1.249	-0.225	0.261	-2.822	-0.022	4.78E-03	16872
Beef intake	-1.566	-2.161	-0.972	0.303	-5.166	-0.040	2.42E-07	16859
Lamb/mutton intake	-2.310	-3.065	-1.556	0.385	-6.001	-0.046	2.00E-09	16850
Pork intake	-2.177	-2.871	-1.482	0.354	-6.140	-0.047	8.42E-10	16839
Cheese intake	-0.379	-0.828	0.071	0.229	-1.652	-0.013	9.86E-02	16591

Notes: The table reports the statistics of the association between total brain grey matter volume (TGMV) dietary phenotypes in the discovery sample. The covariates included are age at follow-up, gender, evaluation sites of MRI scan (dummy variables), and total intracranial volume. Panel D. reports the correlations between TGMV and follow-up dietary measurements, adjusting for socioeconomic factors including average total household income, qualifications, and townsend deprivation index. Beta refers to the coefficient of the regression model. t, p, se, 95%CI_L and 95%CI_H repsepctively represent the t statistic, two-side p-value, standard error, 95% coffidence interval for coefficients. df.e refers to the degree freedom of the residuals of the regression model. r refers to the correlation coefficient.

Supplementary Table 4. Confirmation of the association between TGMV and cereal/coffee intake.

Panel A. Summary information of multiple measures of TGMV obtained from UK Biobank.	
Field ID	Variable
25005	Volume of grey matter (normalised for head size)
25006	Volume of grey matter
26518	Volume of TotalGray (whole brain)

Panel B. Correlations between multiple measurements of TGMV and baseline diet measurements.								
Field ID	beta	95%CI_L	95%CI_H	se	t	r	p	df.e
Cereal intake								
25005	760.627	572.932	948.321	95.758	7.943	0.058	2.08E-15	18722
25006	1143.483	909.710	1377.257	119.267	9.588	0.070	1.01E-21	18722
26518	641.751	490.591	792.912	77.119	8.322	0.061	9.28E-17	18481
Coffee intake								
25005	-1424.739	-1721.605	-1127.872	151.455	-9.407	-0.069	5.68E-21	18652
25006	-771.503	-1142.405	-400.601	189.227	-4.077	-0.030	4.58E-05	18652
26518	-371.902	-611.399	-132.404	122.187	-3.044	-0.022	2.34E-03	18411

Panel C. Correlations between multiple measurements of TGMV and follow-up diet measurements.								
Field ID	beta	95%CI_L	95%CI_H	se	t	r	p	df.e
Cereal intake								
25005	717.845	522.853	912.837	99.481	7.216	0.052	5.56E-13	18853
25006	1230.264	987.825	1472.703	123.688	9.947	0.072	2.98E-23	18853
26518	492.145	335.206	649.083	80.067	6.147	0.045	8.07E-10	18611
Coffee intake								
25005	-1244.918	-1514.548	-975.288	137.560	-9.050	-0.066	1.57E-19	18835
25006	-787.633	-1124.012	-451.254	171.614	-4.590	-0.033	4.47E-06	18835
26518	-326.831	-544.356	-109.306	110.977	-2.945	-0.022	3.23E-03	18593

Panel D. Correlations between TGMV and the intake of cereal and coffee in the replication sample.								
Diet	beta	95%CI_L	95%CI_H	se	t	r	p	df.e
Associations between TGMV and the intake of cereal and coffee at baseline.								
Cereal intake	0.092	0.075	0.109	0.009	10.666	0.083	1.80E-26	16304
Coffee intake	-0.094	-0.122	-0.065	0.015	-6.418	-0.050	1.42E-10	16244
Associations between TGMV and the intake of cereal and coffee at follow-up.								
Cereal intake	0.071	0.053	0.088	0.009	7.843	0.061	4.68E-15	16388
Coffee intake	-0.100	-0.127	-0.073	0.014	-7.385	-0.058	1.60E-13	16307
Associations between TGMV and the intake of cereal and coffee at baseline.								
Cereal intake	0.026	0.005	0.047	0.011	2.438	0.019	1.48E-02	16287
Coffee intake	-0.077	-0.113	-0.042	0.018	-4.237	-0.033	2.28E-05	16159

Notes: The table reports the confirmation of the association between TGMV and cereal/coffee intake. Panel A summaries information of multiple measurements of TGMV directly obtained from UK Biobank. Panel B to C report the statistics of the correlation between multiple measurements of TGMV and the intake of cereal and coffee in the discovery sample. Panel D reports the correlation between TGMV and cereal/coffee intake. The covariates included in the models are age at follow-up, gender, evaluation sites of MRI scan (dummy variables), and total intracranial volume. Beta refers to the coefficient of the regression model. t, p, se, 95%CI_L and 95%CI_H repsepctively represent the t statistic, two-side p-value, standard error, 95% coffidence interval for coefficients. df.e refers to the degree freedom of the residuals of the regression model. r refers to the correlation coefficient.

Supplementary Table 5. Genome-wide independent significant variants of the cereal intake.

CHR	SNP	BP	A1	A2	N	BETA	STAT	P	Is lead SNP
1	rs17030613	113190807	C	A	335090	0.04525	5.465	4.63E-08	yes
2	rs10190898	60171757	G	A	334076	0.04518	5.758	8.54E-09	yes
3	rs7618975	25086328	C	A	334112	0.03832	5.593	2.24E-08	no
3	rs4858694	25097736	A	G	334974	0.04652	6.351	2.14E-10	yes
3	rs7643390	25174059	T	C	334844	0.03853	5.757	8.56E-09	no
3	rs720822	25232155	A	G	334631	0.03745	5.602	2.12E-08	no
3	rs1483848	25316283	A	G	334342	-0.03749	-5.59	2.28E-08	no
3	rs1562502	35735596	T	C	334961	0.0374	5.483	4.20E-08	yes
3	rs2236950	50420554	A	C	335339	0.04909	5.607	2.06E-08	yes
5	rs358288	87495504	G	A	335175	-0.06738	-6.725	1.76E-11	yes
5	rs11748798	87650742	T	G	334620	-0.04261	-5.523	3.34E-08	no
6	rs9381903	50773228	A	G	334734	-0.03654	-5.46	4.76E-08	yes
6	rs13202780	51180392	C	T	334584	-0.04685	-6.363	1.99E-10	yes
6	rs2504706	51258579	C	T	334485	0.05759	7.298	2.94E-13	yes
6	rs1342613	51302471	T	G	334625	-0.04519	-5.622	1.89E-08	no
6	rs9401196	98274551	G	A	333167	-0.04266	-6.312	2.76E-10	no
6	rs7770226	98320402	T	C	334723	-0.04334	-6.362	1.99E-10	no
6	rs9401305	98331937	C	A	334610	0.04835	7.204	5.87E-13	yes
6	rs9482094	98364895	A	G	334516	0.03891	5.612	2.00E-08	no
6	rs4524616	98445008	C	A	334599	0.04724	7	2.57E-12	no
6	rs4557524	98466195	T	G	334289	-0.04112	-6.128	8.93E-10	no
6	rs4601136	98488771	G	A	334855	-0.04232	-6.27	3.61E-10	no
6	rs62422661	98545611	T	G	334865	0.04294	6.272	3.58E-10	no
7	rs10278591	1921362	T	C	334728	0.05088	5.84	5.23E-09	no
7	rs7791299	1935598	C	T	330905	0.04897	5.458	4.83E-08	no
7	rs56259105	1978002	C	T	334205	0.0514	5.966	2.43E-09	yes
7	rs34809719	2028968	T	G	334937	0.05096	5.896	3.73E-09	no
7	rs56226325	2078981	T	C	335026	0.0526	5.668	1.44E-08	no
7	rs4410790	17284577	C	T	334375	-0.03806	-5.474	4.40E-08	yes
8	rs2670028	57423673	G	A	335051	-0.04246	-5.474	4.42E-08	yes
9	rs7038814	86733355	G	A	335050	0.03847	5.567	2.60E-08	no
9	rs2799849	86752641	C	T	334839	0.04467	6.245	4.24E-10	yes
9	rs1549314	127910307	G	T	335235	0.03781	5.644	1.66E-08	yes
9	rs501963	127962916	A	G	335170	0.03682	5.495	3.90E-08	no
11	rs566534	28730053	C	T	334372	0.04044	5.601	2.14E-08	yes
15	rs12440952	74615292	G	A	334998	0.04206	5.991	2.09E-09	no
15	rs2959005	74618128	C	T	335121	0.04335	6.098	1.08E-09	no
15	rs1564782	74622678	G	A	335313	0.04226	5.791	6.99E-09	no
15	rs4077582	74665622	C	T	334477	0.04236	5.785	7.28E-09	no
15	rs11856835	74716174	G	A	333176	0.04702	7.004	2.49E-12	no
15	rs750607	74734819	C	T	331243	0.04175	6.141	8.19E-10	no
15	rs2470893	75019449	T	C	333685	-0.05338	-7.474	7.78E-14	no
15	rs2472297	75027880	T	C	334995	-0.05918	-7.844	4.38E-15	yes
18	rs12709578	1834649	T	G	334811	-0.04464	-6.543	6.05E-11	no
18	rs72867256	1849194	A	G	335530	-0.08093	-5.64	1.71E-08	no
18	rs555692	1883692	T	C	334338	-0.04216	-6.274	3.53E-10	no
18	rs2289322	1884329	T	G	334933	-0.07967	-8.439	3.20E-17	yes
18	rs9951259	1898350	A	G	334936	-0.04384	-6.31	2.79E-10	no
19	rs78816499	42596056	C	T	335429	0.07298	5.548	2.90E-08	yes
19	rs769449	45410002	A	G	334498	0.05718	5.685	1.31E-08	yes
19	rs2287921	49228272	T	C	335253	-0.03858	-5.751	8.89E-09	no
19	rs2287922	49232226	G	A	334277	-0.03893	-5.791	7.00E-09	yes

Notes: The table reports the genome-wide independent significant variants of cereal intake. The results were from the genome-wide association analysis of the cereal intake at baseline, which was perfomed by Plink [1] v1.9 software using a linear regression model. The covariates included are baseline age, gender, and the top 40 genetic components obtained from UK Biobank. BETA refers to the coefficient of the regression model. STAT and P repseptively represent the t statistic and the two-side p-value for coefficients. N refers to the refers to the effect sample size for each variant. CHR, SNP, BP, A1 and A2 represent the chromosome, name, base-pair distance, effect allele and non-effec allele for the variant, respectively. The independent significant variant and the lead SNP were defined using FUMA [2] software with default parameters.

References

[1] S. Purcell et al., PLINK: a tool set for whole-genome association and population-based linkage analyses. The American journal of human genetics 81, 559-575 (2007).

[2] K. Watanabe, E. Taskesen, A. Van Bochoven, D. Posthuma, Functional mapping and annotation of genetic associations with FUMA. *Nature communications* **8**, 1826 (2017).

Supplementary Table 6. Genome-wide independent significant variants of the coffee intake.

CHR	SNP	BP	A1	A2	N	BETA	STAT	P	Is lead SNP
2	rs62106258	417167	C	T	334871	-0.06039	-5.48	4.25E-08	yes
2	rs2947411	614168	A	G	334825	-0.04882	-7.831	4.86E-15	no
2	rs2867125	622827	T	C	334788	-0.04838	-7.729	1.09E-14	no
2	rs11127485	632028	C	T	333775	-0.04937	-7.87	3.57E-15	no
2	rs6548238	634905	T	C	334844	-0.04995	-7.961	1.72E-15	yes
2	rs13393304	637830	A	G	334396	-0.0495	-7.862	3.81E-15	no
2	rs7561317	644953	A	G	334808	-0.04936	-7.884	3.17E-15	no
2	rs1260326	27730940	T	C	333668	-0.03923	-8.083	6.34E-16	yes
2	rs780094	27741237	T	C	334770	-0.03783	-7.765	8.18E-15	no
2	rs780093	27742603	T	C	334480	-0.03773	-7.734	1.04E-14	no
2	rs780092	27743154	G	A	334828	0.03828	5.935	2.94E-09	no
2	rs1260333	27748624	A	G	334537	-0.03502	-7.337	2.19E-13	no
2	rs1919127	27801493	C	T	334293	-0.03374	-6.195	5.82E-10	no
2	rs1919128	27801759	G	A	334714	-0.0336	-6.178	6.49E-10	no
2	rs13022873	27815510	C	A	333508	-0.03247	-5.933	2.97E-09	no
2	rs12987055	27834152	C	T	334340	-0.03425	-6.279	3.40E-10	no
2	rs3749147	27851918	A	G	334033	-0.03355	-6.116	9.60E-10	no
4	rs58115360	2865107	T	C	334607	-0.03048	-5.832	5.47E-09	yes
4	rs6824567	2883172	T	C	334500	-0.02993	-5.687	1.30E-08	no
4	rs2231164	89015857	C	T	334759	-0.03846	-5.472	4.46E-08	no
4	rs2231142	89052323	T	G	334794	-0.05224	-7.004	2.49E-12	yes
5	rs12519880	7391434	A	C	334047	-0.03241	-6.158	7.39E-10	yes
5	rs1422192	87959023	A	G	333513	0.03959	6.059	1.38E-09	yes
5	rs618741	88064012	C	T	334555	0.02698	5.627	1.84E-08	no
5	rs304132	88215594	A	G	334297	0.02629	5.487	4.09E-08	yes
6	rs2517618	30158127	A	G	334574	0.03493	5.479	4.28E-08	no
6	rs3132649	30321057	A	G	334850	0.03683	5.517	3.44E-08	no
6	rs3094061	30321189	C	A	334728	0.03725	5.58	2.41E-08	no
6	rs3130374	30321336	T	C	334492	0.03688	5.526	3.28E-08	no
6	rs3130375	30321732	A	C	334557	0.03704	5.542	3.00E-08	no
6	rs3131038	30734080	A	G	334432	0.03041	5.459	4.81E-08	no
6	rs1264350	30796545	C	T	334508	0.03526	5.528	3.24E-08	no
6	rs3131787	30899524	C	T	334619	0.03198	5.558	2.73E-08	no
6	rs3095151	30900150	T	G	334436	0.03188	5.537	3.08E-08	no
6	rs3130781	30914552	C	T	334723	0.03179	5.534	3.13E-08	no
6	rs2247056	31265490	T	C	329376	0.02861	5.456	4.87E-08	no
6	rs3094005	31465047	T	G	334717	0.03767	5.546	2.92E-08	no
6	rs3132450	31596138	G	A	334394	0.03919	5.647	1.63E-08	no
6	rs3117582	31620520	G	T	334928	0.03948	5.688	1.29E-08	no
6	rs3132449	31626013	T	C	334913	0.03935	5.673	1.41E-08	no
6	rs805293	31688518	A	T	334725	-0.03005	-6.317	2.66E-10	no
6	rs3131383	31704294	T	G	334781	0.03933	5.666	1.46E-08	no
6	rs3101018	31705864	T	C	334725	0.03863	5.56	2.71E-08	no
6	rs3130484	31715882	C	T	334531	0.03932	5.659	1.52E-08	no
6	rs3131379	31721033	A	G	334825	0.03925	5.654	1.57E-08	no
6	rs3117574	31725230	A	G	334841	0.04029	5.801	6.59E-09	no
6	rs3131378	31725285	G	A	334993	0.03927	5.659	1.52E-08	no
6	rs3117575	31726253	C	T	334785	0.03926	5.659	1.52E-08	no
6	rs3117577	31727474	G	A	334706	0.0389	5.604	2.09E-08	no
6	rs3115671	31734345	T	G	334835	0.03918	5.641	1.70E-08	no
6	rs3130490	31739120	T	G	334428	0.03985	5.714	1.10E-08	no
6	rs3130679	31807540	G	A	334074	0.04115	5.919	3.25E-09	no

6	rs521977	31836827	T	G	334634	0.03529	6.978	2.99E-12	no
6	rs660594	31837250	G	A	334649	0.03064	6.462	1.03E-10	no
6	rs660550	31837277	C	A	334773	0.03109	6.561	5.35E-11	no
6	rs577272	31837963	G	A	334268	0.03092	6.524	6.86E-11	no
6	rs644827	31838441	T	C	334745	0.03087	6.515	7.29E-11	no
6	rs644774	31838490	C	T	334692	0.03087	6.515	7.29E-11	no
6	rs2242665	31839309	C	T	334641	0.03036	6.404	1.51E-10	no
6	rs3132442	31839494	C	T	334559	0.03035	6.409	1.47E-10	no
6	rs3130482	31839782	C	A	334577	0.0301	6.358	2.05E-10	no
6	rs605203	31847012	C	A	333769	0.03414	6.919	4.56E-12	no
6	rs652888	31851234	G	A	334334	0.03107	5.556	2.76E-08	no
6	rs486416	31856070	G	A	333954	0.03315	6.74	1.59E-11	no
6	rs535586	31860337	T	C	334532	0.03377	6.872	6.34E-12	no
6	rs659445	31864304	G	A	334444	0.03408	6.937	4.01E-12	no
6	rs558702	31870326	A	G	334803	0.03924	5.633	1.78E-08	no
6	rs497309	31892484	C	A	334859	0.03941	5.655	1.56E-08	no
6	rs537160	31916400	A	G	333501	0.0305	6.165	7.04E-10	no
6	rs1270942	31918860	G	A	334507	0.03839	5.512	3.55E-08	no
6	rs630379	31922254	A	C	334128	0.03109	6.127	8.96E-10	no
6	rs440454	31927342	A	G	333113	0.03034	5.991	2.09E-09	no
6	rs419788	31928799	T	C	334363	0.03071	6.078	1.22E-09	no
6	rs437179	31929014	A	C	333753	0.02981	5.892	3.81E-09	no
6	rs410851	31936668	T	C	334732	0.03175	6.279	3.41E-10	no
6	rs389884	31940897	G	A	333863	0.03956	5.665	1.48E-08	no
6	rs389883	31947460	G	T	334545	0.03094	6.123	9.21E-10	no
6	rs1150753	32059867	G	A	334784	0.0398	5.702	1.18E-08	no
6	rs1150752	32064726	C	T	334792	0.03917	5.609	2.04E-08	no
6	rs1269852	32080191	C	G	334624	0.04111	5.87	4.36E-09	no
6	rs3130288	32096001	A	C	334496	0.04071	5.808	6.32E-09	no
6	rs3130349	32147696	A	G	334807	0.03251	5.495	3.90E-08	no
6	rs1062070	32148031	G	A	334144	0.03274	5.501	3.77E-08	no
6	rs3134605	32159956	C	T	334787	0.03295	5.759	8.45E-09	no
6	Affx-28468489	32165444	G	A	332971	0.03604	5.642	1.68E-08	no
6	rs3132947	32176782	T	G	334211	0.03253	5.66	1.51E-08	no
6	rs3096690	32194630	C	G	334354	0.03875	5.579	2.42E-08	no
6	rs6936204	32217092	T	C	334472	0.02707	5.527	3.27E-08	no
6	rs3130320	32223258	T	C	334395	0.0271	5.556	2.77E-08	no
6	rs7756177	32289358	A	G	334437	0.03392	5.774	7.77E-09	no
6	rs3129949	32298814	A	C	334232	0.03078	5.55	2.86E-08	no
6	rs1003878	32299822	A	G	334584	0.03105	5.605	2.08E-08	no
6	rs1265757	32302382	T	C	334838	0.03853	5.497	3.87E-08	no
6	rs2395149	32325562	A	G	334798	0.03821	5.456	4.87E-08	no
6	rs3129927	32333827	C	A	334725	0.03825	5.464	4.66E-08	no
6	rs2858324	32660375	A	G	332865	0.02839	5.859	4.66E-09	no
6	rs2647012	32664458	T	C	334466	0.02876	5.963	2.48E-09	no
6	rs2647003	32664880	T	G	332652	0.02934	6.07	1.28E-09	no
6	rs2647046	32668336	A	C	334679	0.02812	5.828	5.60E-09	no
6	rs2856717	32670308	A	G	334223	0.02792	5.787	7.15E-09	no
6	rs2858305	32670464	G	T	332198	0.02661	5.473	4.42E-08	no
6	rs9275572	32678999	A	G	334631	0.02617	5.466	4.61E-08	no
6	rs9275596	32681631	C	T	329449	0.02714	5.535	3.11E-08	no
6	rs2504706	51258579	C	T	333860	-0.03371	-6.027	1.67E-09	yes
7	rs7805128	17106017	G	T	333705	-0.02893	-6.062	1.35E-09	yes
7	rs6973364	17160612	T	C	333541	0.02894	5.902	3.59E-09	no

7	rs10950647	17196529	G	A	334015	-0.04465	-9.378	6.79E-21	yes
7	rs112476491	17204040	A	G	334464	-0.07753	-5.812	6.17E-09	no
7	rs4721583	17215812	G	A	334196	0.0335	5.934	2.95E-09	no
7	rs13328338	17221091	G	A	333272	0.02655	5.455	4.90E-08	no
7	rs4476901	17243954	G	A	334362	0.05193	7.54	4.70E-14	yes
7	rs74919529	17269707	T	C	327881	-0.07873	-6.436	1.23E-10	yes
7	Affx-30120430	17273374	CT	C	334700	-0.05107	-9.319	1.18E-20	no
7	rs12539940	17275597	C	T	334196	0.0532	5.903	3.56E-09	no
7	rs78707595	17279393	T	C	334876	0.111	7.263	3.80E-13	no
7	rs4410790	17284577	C	T	333749	0.12	24.37	4.71E-131	yes
7	rs1117203	17303549	A	G	334210	-0.05868	-11.03	2.74E-28	no
7	rs12670403	17309279	C	A	334606	-0.09552	-20.18	1.72E-90	no
7	rs111577430	17319257	G	A	332704	-0.1144	-6.143	8.12E-10	no
7	rs10272066	17374902	C	T	333280	-0.07016	-12.48	1.01E-35	yes
7	rs2066853	17379110	A	G	334344	-0.08551	-11.1	1.23E-28	no
7	rs113070557	17382217	G	T	334679	-0.1239	-7.813	5.60E-15	yes
7	rs62446370	17404160	A	G	334181	0.06413	5.972	2.35E-09	yes
7	rs2198497	17414133	G	A	325214	-0.05438	-8.801	1.36E-18	no
7	rs11764350	17422113	T	A	331955	-0.03137	-5.977	2.28E-09	no
7	rs80265229	17465828	T	C	334506	0.05936	7.855	4.02E-15	yes
7	rs2034684	17475607	C	T	333131	0.04152	7.626	2.44E-14	no
7	rs74789644	17488791	G	C	333474	-0.07512	-7.709	1.27E-14	yes
7	rs606056	17501082	C	A	333974	-0.03453	-7.242	4.44E-13	no
7	rs4721617	17507948	G	A	334420	0.04506	8.25	1.58E-16	no
7	rs6963451	17517644	G	T	334350	-0.06862	-9.524	1.68E-21	no
7	rs11761325	17552884	A	G	332012	-0.03797	-6.615	3.71E-11	yes
7	rs4717763	72825125	A	G	334128	0.02666	5.535	3.11E-08	no
7	rs71556711	72854549	T	C	334600	0.04771	5.788	7.13E-09	no
7	rs2240466	72856269	A	G	334858	0.05365	7.475	7.75E-14	no
7	rs1178979	72856430	C	T	334695	0.04142	6.97	3.18E-12	no
7	rs714052	72864869	G	A	333967	0.05312	7.411	1.26E-13	no
7	rs12056034	72878645	G	A	332370	0.05353	7.467	8.25E-14	no
7	rs17145713	72904810	T	C	334453	0.04116	6.922	4.47E-12	no
7	rs13233571	72971231	T	C	334401	0.05561	7.744	9.63E-15	no
7	rs17145738	72982874	T	C	334610	0.05516	7.665	1.79E-14	no
7	rs2286276	72987354	T	C	334711	0.03813	7.281	3.32E-13	no
7	rs1051921	73007943	A	G	334795	0.04321	7.29	3.12E-13	no
7	rs35332062	73012042	A	G	334765	0.05382	7.609	2.77E-14	no
7	rs35368205	73017657	T	C	334026	0.04288	7.25	4.17E-13	no
7	rs3812316	73020337	G	C	334937	0.05593	7.924	2.31E-15	yes
7	rs17145750	73026378	T	C	334165	0.04697	7.305	2.78E-13	no
7	rs34958196	73043101	A	G	334255	0.05462	7.751	9.13E-15	no
7	rs41299460	75600951	A	G	333784	0.03619	7.414	1.23E-13	no
7	rs1057868	75615006	T	C	334836	0.06098	11.65	2.36E-31	yes
7	rs17685	75616105	A	G	334742	0.0589	11.16	6.37E-29	no
7	rs8565	75630274	T	C	334610	0.05392	10.08	7.05E-24	no
7	Affx-30886472	75659815	T	C	334235	0.04213	8.163	3.29E-16	no
7	rs61303167	75671175	T	G	334555	0.04507	7.09	1.35E-12	no
7	rs79859948	75679099	G	A	334402	0.04716	5.991	2.08E-09	no
7	rs74999892	75756464	C	T	333917	0.04668	5.929	3.06E-09	no
7	rs56343450	75796235	T	C	334424	0.04232	8.209	2.24E-16	no
7	rs78663390	75845837	A	C	333340	0.04387	6.671	2.55E-11	no
12	rs2599415	11217237	G	A	331414	0.03613	5.635	1.75E-08	no
12	rs61928609	11316437	A	C	334259	0.03771	5.893	3.81E-09	yes

12	rs1669413	11338781	C	A	334631	0.03721	5.832	5.48E-09	no
12	rs1669424	11346562	T	C	334480	0.03636	5.696	1.23E-08	no
15	rs12914489	74187937	A	G	334708	0.06171	8.106	5.25E-16	yes
15	rs5742914	74286929	T	C	334713	0.06184	8.463	2.61E-17	no
15	Affx-11962945	74467796	T	C	334262	0.05894	10.3	6.99E-25	no
15	rs1052622	74467856	G	A	334191	0.04713	9.292	1.52E-20	yes
15	rs34868798	74469716	C	T	334744	0.05902	10.34	4.93E-25	no
15	rs351224	74487036	A	T	333823	-0.03089	-6.476	9.42E-11	no
15	rs11857410	74488424	A	G	334671	0.06163	10.76	5.37E-27	no
15	rs74922218	74516615	A	C	331201	0.09201	5.543	2.98E-08	no
15	rs11635191	74550050	A	C	334055	-0.03848	-6.468	9.95E-11	no
15	rs12916871	74552742	C	A	334352	0.04682	9.796	1.18E-22	no
15	rs28395230	74557835	C	T	327973	-0.04784	-8.068	7.17E-16	yes
15	rs1038436	74560566	C	T	333734	-0.03851	-7.445	9.69E-14	no
15	rs16967313	74561301	A	G	333238	-0.04426	-6.706	2.00E-11	no
15	rs901130	74573907	G	C	334384	-0.0339	-6.429	1.28E-10	no
15	rs4887131	74598757	A	C	334065	-0.04562	-9.224	2.88E-20	no
15	rs1009176	74603484	T	C	334865	-0.05065	-7.546	4.50E-14	no
15	rs12440952	74615292	G	A	334370	-0.05075	-10.2	1.99E-24	no
15	rs2959005	74618128	C	T	334493	-0.05076	-10.07	7.26E-24	no
15	rs1564782	74622678	G	A	334687	-0.04932	-9.534	1.52E-21	no
15	rs4077582	74665622	C	T	333853	-0.04874	-9.388	6.12E-21	no
15	rs28362906	74711790	A	G	334407	0.1034	10.49	1.02E-25	no
15	rs11856835	74716174	G	A	332558	-0.07035	-14.79	1.85E-49	no
15	rs8036030	74716609	A	G	334554	-0.06242	-12.78	2.11E-37	no
15	rs750607	74734819	C	T	330624	-0.05822	-12.08	1.32E-33	no
15	rs35032603	74753425	C	T	334391	0.1195	9.549	1.32E-21	yes
15	rs80025017	74845566	A	C	333788	-0.05471	-6.15	7.77E-10	no
15	rs62005806	74883323	A	G	334239	0.1111	12.28	1.25E-34	no
15	rs4128436	74935894	T	C	334374	-0.05794	-6.667	2.62E-11	yes
15	rs145923939	74961238	C	T	334541	0.1073	5.874	4.27E-09	yes
15	rs2606345	75017176	C	A	333843	-0.05203	-10.28	8.67E-25	no
15	rs2470893	75019449	T	C	333057	0.1174	23.19	7.37E-119	no
15	rs2472297	75027880	T	C	334365	0.1419	26.55	4.20E-155	yes
15	rs2472299	75033400	A	G	334699	-0.06438	-12.1	1.13E-33	no
15	rs762551	75041917	C	A	334695	-0.06331	-11.89	1.33E-32	no
15	rs45468096	75043539	T	C	334638	-0.4812	-9.012	2.04E-19	yes
15	rs28399419	75043669	T	C	334414	-0.157	-6.45	1.12E-10	yes
15	rs2472304	75044238	G	A	334693	-0.06658	-13.22	6.90E-40	no
15	rs2470890	75047426	C	T	334575	-0.06623	-13.15	1.78E-39	no
15	rs936226	75069282	C	T	333752	-0.06121	-11.31	1.15E-29	no
15	rs1378942	75077367	C	A	334640	-0.06221	-12.26	1.56E-34	no
15	rs34862454	75101530	C	T	333910	-0.06001	-11.87	1.79E-32	no
15	rs6495122	75125645	A	C	334609	-0.0615	-12.74	3.73E-37	no
15	rs55973697	75129584	C	A	334682	0.1299	7.295	3.00E-13	yes
15	rs12898397	75130093	T	C	334075	-0.05828	-11.85	2.26E-32	no
15	rs4886615	75131661	A	G	334646	-0.05062	-9.538	1.47E-21	no
15	rs6938	75136261	C	G	332531	-0.05281	-10.16	2.91E-24	yes
15	rs2305668	75142761	G	T	334547	-0.04528	-6.004	1.93E-09	no
15	rs11630918	75155896	C	T	334268	-0.05996	-12.53	5.51E-36	no
15	rs12911254	75166335	G	A	334247	-0.05757	-12.04	2.11E-33	no
15	rs1130741	75189930	A	G	334515	-0.05355	-11.17	5.71E-29	no
15	rs6495127	75194490	C	T	334241	-0.04989	-9.522	1.71E-21	no
15	rs12907898	75207872	C	T	332523	-0.05264	-10.94	7.82E-28	no

15	rs12912839	75221245	C	T	331594	-0.05774	-12.04	2.17E-33	no
15	rs12912343	75251041	C	G	334644	-0.0632	-13.32	1.76E-40	no
15	rs6495133	75255458	G	C	334278	-0.04834	-7.635	2.26E-14	yes
15	rs62029217	75261899	C	T	334406	-0.06298	-13.27	3.56E-40	no
15	rs12050759	75265191	A	G	334337	-0.0411	-8.011	1.14E-15	no
15	rs11636892	75266049	G	C	333799	-0.06189	-13.04	7.30E-39	no
15	rs34359839	75274669	G	A	334369	-0.049	-7.005	2.48E-12	no
15	rs3812945	75289722	C	T	334093	0.06489	13.69	1.26E-42	no
15	rs8030089	75290377	T	C	334530	-0.03527	-5.815	6.07E-09	no
15	rs2289582	75310394	C	T	334353	-0.03371	-5.543	2.98E-08	no
15	rs8042558	75320433	T	G	333921	-0.04071	-7.276	3.44E-13	no
15	rs12916473	75321999	A	G	334474	0.1124	8.899	5.67E-19	no
15	rs34835684	75331805	G	T	334240	-0.03366	-5.564	2.64E-08	no
15	rs8043181	75380424	C	T	334740	-0.03479	-5.946	2.75E-09	no
15	rs12148488	75382542	T	G	330604	-0.0604	-12.62	1.77E-36	no
15	rs8034281	75384227	T	C	334596	-0.04834	-9.869	5.73E-23	no
15	rs11638746	75403108	C	A	334380	-0.04516	-8.635	5.92E-18	no
15	rs1602360	75420761	G	A	320676	-0.04432	-8.21	2.22E-16	no
15	rs34511319	75429717	A	C	334246	-0.04708	-8.739	2.36E-18	no
15	rs8025412	75444033	C	T	334634	-0.04602	-9.127	7.08E-20	no
15	rs58201389	75446835	G	A	334383	-0.04643	-9.201	3.56E-20	no
15	rs8040372	75450557	A	G	334113	-0.04843	-9.847	7.11E-23	no
15	rs7170378	75457490	T	C	333733	-0.04431	-9.065	1.25E-19	no
15	rs11072530	75458850	G	A	334606	-0.04472	-9.091	9.85E-20	no
15	rs2201002	75463150	G	A	334109	-0.04453	-9.127	7.05E-20	yes
15	rs1873379	75498744	C	G	330117	0.05366	10.2	1.99E-24	no
15	rs1984586	75607479	A	G	333858	0.04335	8.16	3.37E-16	no
15	rs55653892	75610684	C	G	334603	0.0418	7.816	5.45E-15	no
15	rs111858375	75614821	A	C	334649	0.04288	8.089	6.02E-16	no
15	rs115991344	75614973	T	C	334625	0.04218	7.96	1.73E-15	no
15	rs77532275	75690292	C	A	334767	0.04236	7.932	2.16E-15	no
15	rs75782768	75692186	T	C	334498	0.04237	7.923	2.33E-15	no
15	rs112994348	75698201	T	C	332588	0.06587	6.421	1.36E-10	no
15	rs34608804	75718459	C	T	333648	0.06845	5.749	8.99E-09	no
15	rs78428611	75738292	A	C	334565	0.04254	7.956	1.79E-15	no
15	rs117174465	75753810	G	A	334669	0.1026	5.941	2.83E-09	no
15	rs76550488	75757050	C	G	334164	0.04422	8.27	1.35E-16	no
15	rs80163632	75763377	G	A	334120	0.04364	8.164	3.26E-16	no
15	rs78521534	75769347	T	C	322729	0.05104	7.273	3.51E-13	yes
15	rs79471369	75771103	C	T	334515	0.04391	8.22	2.04E-16	no
15	rs76063558	75775476	A	G	334488	0.04374	8.181	2.82E-16	no
15	rs77716675	75781554	G	A	334320	0.04352	8.135	4.14E-16	no
15	rs76621678	75844953	T	C	334424	0.03877	6.164	7.09E-10	no
15	rs118028692	75847217	A	C	334272	0.08481	6.081	1.20E-09	no
15	rs74845979	75912699	T	C	334730	0.04398	8.098	5.59E-16	no
15	rs78911138	76275641	T	G	333859	0.06926	6.872	6.35E-12	yes
15	rs868299	77873549	A	G	333986	-0.02731	-5.534	3.14E-08	yes
15	rs11330240	91428521	C	CT	334276	-0.02757	-5.459	4.80E-08	no
15	rs7497304	91429176	T	G	334505	-0.02761	-5.467	4.57E-08	yes
16	rs9940128	53800754	A	G	334223	0.03791	7.899	2.83E-15	no
16	rs1421085	53800954	C	T	334254	0.03859	7.987	1.39E-15	no
16	rs1121980	53809247	A	G	334456	0.03797	7.916	2.45E-15	no
16	rs17817449	53813367	G	T	334387	0.03819	7.876	3.39E-15	no
16	rs8050136	53816275	A	C	334609	0.03826	7.887	3.10E-15	no

16	rs3751812	53818460	T	G	334637	0.03817	7.873	3.49E-15	no
16	rs9939609	53820527	A	T	334878	0.03806	7.856	3.99E-15	no
16	rs7202116	53821615	G	A	334500	0.03805	7.847	4.28E-15	no
16	rs9941349	53825488	T	C	333785	0.03866	8.004	1.21E-15	yes
16	rs9930506	53830465	G	A	331183	0.03627	7.558	4.12E-14	no
16	rs9922619	53831771	T	G	333782	0.03659	7.64	2.18E-14	no
16	rs12149832	53842908	A	G	334593	0.03723	7.739	1.01E-14	no
16	rs11642841	53845487	A	C	334752	0.03354	6.938	3.97E-12	no
17	Affx-13612776	17579861	G	C	334321	-0.02699	-5.701	1.20E-08	no
17	rs11649804	17696755	A	C	334655	-0.03184	-6.105	1.03E-09	yes
17	rs12936927	17726965	C	T	334125	-0.02897	-5.802	6.56E-09	no
17	rs3744115	17749822	G	A	334674	-0.02754	-5.534	3.13E-08	no
17	rs11078405	17824978	T	G	334446	-0.02901	-5.914	3.35E-09	no
18	rs4121765	57730096	G	A	334400	0.02963	5.747	9.07E-09	no
18	rs36030660	57735945	C	T	334167	0.03778	7.066	1.60E-12	no
18	rs7240566	57744189	G	A	334589	0.04109	7.498	6.49E-14	no
18	rs35981845	57758860	G	A	334533	0.02991	5.961	2.50E-09	no
18	rs633265	57831468	T	G	334409	0.02662	5.579	2.42E-08	no
18	rs571312	57839769	A	C	334744	0.04742	8.489	2.09E-17	no
18	rs589850	57841891	A	G	334385	0.02726	5.718	1.08E-08	no
18	rs1350341	57842533	A	G	334624	0.02708	5.683	1.33E-08	no
18	rs17782313	57851097	C	T	334156	0.04803	8.576	9.86E-18	no
18	rs10871777	57851763	G	A	334610	0.04878	8.774	1.74E-18	yes
18	rs489693	57882787	A	C	334587	0.03563	7.052	1.77E-12	no
18	rs12970134	57884750	A	G	334334	0.0418	7.815	5.50E-15	no
19	rs3810291	47569003	G	A	334674	-0.0284	-5.611	2.01E-08	yes
20	rs6062359	62900380	G	T	333934	0.03034	6.344	2.24E-10	yes

Notes: The table reports the genome-wide independent significant variants of the coffee intake. The results were from the genome-wide association analysis of the coffee intake at baseline, which was perfomed by Plink [1] v1.9 software using a linear regression model. The covariates included are baseline age, gender, and the top 40 genetic components obtained from UK Biobank. BETA refers to the coefficient of the regression model. STAT and P repsepctively represent the t statistic and the two-side p-value for coefficients. N refers to the refers to the effect sample size for each variant. CHR, SNP, BP, A1 and A2 represent the chromosome, name, base-pair distance, effect allele and non-effec allele for the variant, respectively. The independent significant variant and the lead SNP were defined using FUMA [2] software with default parameters.

References

[1] S. Purcell et al., PLINK: a tool set for whole-genome association and population-based linkage analyses. The American journal of human genetics 81, 559-575 (2007).

[2] K. Watanabe, E. Taskesen, A. Van Bochoven, D. Posthuma, Functional mapping and annotation of genetic associations with FUMA. *Nature communications* **8**, 1826 (2017).

Supplementary Table 7. Associations between the share lead variants and diet measurements.

Panel A. Association between the C allele of rs2504706 and diet measurements.								
Variable	beta	95%CI_L	95%CI_H	se	t	r	p	df.e
Water intake	0.012	0.001	0.024	0.006	2.13	0.004	3.28E-02	331990
Tea intake	0.057	0.043	0.071	0.007	7.73	0.013	1.05E-14	332621
Cooked vegetable intake	-0.001	-0.008	0.007	0.004	-0.16	0.000	8.70E-01	329048
Salad / raw vegetable intake	0.011	0.001	0.020	0.005	2.25	0.004	2.42E-02	330099
Fresh fruit intake	0.034	0.026	0.042	0.004	8.53	0.015	1.53E-17	333101
Dried fruit intake	0.012	0.005	0.018	0.003	3.44	0.006	5.86E-04	329367
Bread intake	-0.042	-0.085	0.002	0.022	-1.89	-0.003	5.92E-02	329979
Oily fish intake	0.002	-0.003	0.007	0.003	0.82	0.001	4.10E-01	333791
Non-oily fish intake	-0.008	-0.012	-0.003	0.002	-3.49	-0.006	4.82E-04	333746
Processed meat intake	0.004	-0.002	0.010	0.003	1.41	0.002	1.60E-01	334794
Poultry intake	0.001	-0.004	0.006	0.002	0.54	0.001	5.89E-01	334720
Beef intake	0.005	0.001	0.010	0.002	2.25	0.004	2.41E-02	334023
Lamb/mutton intake	0.011	0.007	0.015	0.002	5.64	0.010	1.67E-08	333335
Pork intake	0.005	0.001	0.009	0.002	2.51	0.004	1.21E-02	333277
Cheese intake	-0.011	-0.017	-0.005	0.003	-3.47	-0.006	5.20E-04	327491

Panel B. Association between the C allele of rs4410790 and diet measurements.								
Variable	beta	95%CI_L	95%CI_H	se	t	r	p	df.e
Water intake	-0.075	-0.085	-0.065	0.005	-14.64	-0.025	1.65E-48	331879
Tea intake	0.111	0.098	0.123	0.006	17.04	0.030	4.58E-65	332509
Cooked vegetable intake	0.003	-0.004	0.010	0.003	0.85	0.001	3.94E-01	328938
Salad / raw vegetable intake	-0.009	-0.017	0.000	0.004	-2.01	-0.004	4.42E-02	329995
Fresh fruit intake	-0.012	-0.019	-0.005	0.004	-3.45	-0.006	5.59E-04	332986
Dried fruit intake	-0.003	-0.009	0.003	0.003	-0.94	-0.002	3.45E-01	329257
Bread intake	0.041	0.003	0.080	0.020	2.12	0.004	3.43E-02	329867
Oily fish intake	-0.001	-0.005	0.004	0.002	-0.25	0.000	8.04E-01	333675
Non-oily fish intake	-0.002	-0.006	0.001	0.002	-1.22	-0.002	2.22E-01	333631
Processed meat intake	0.007	0.002	0.012	0.003	2.73	0.005	6.36E-03	334680
Poultry intake	0.003	-0.002	0.007	0.002	1.15	0.002	2.51E-01	334607
Beef intake	0.002	-0.002	0.007	0.002	1.19	0.002	2.35E-01	333914
Lamb/mutton intake	0.001	-0.003	0.004	0.002	0.36	0.001	7.16E-01	333226
Pork intake	0.002	-0.002	0.005	0.002	1.02	0.002	3.08E-01	333170
Cheese intake	-0.003	-0.008	0.002	0.003	-1.11	-0.002	2.67E-01	327380

Panel C. Association between the T allele of rs2472297 and diet measurements.								
Variable	beta	95%CI_L	95%CI_H	se	t	r	p	df.e
Water intake	-0.086	-0.097	-0.076	0.006	-15.62	-0.027	5.53E-55	332497
Tea intake	0.148	0.134	0.162	0.007	21.03	0.036	3.82E-98	333124
Cooked vegetable intake	-0.005	-0.012	0.003	0.004	-1.27	-0.002	2.06E-01	329549
Salad / raw vegetable intake	-0.005	-0.014	0.004	0.005	-1.12	-0.002	2.62E-01	330603
Fresh fruit intake	-0.020	-0.027	-0.013	0.004	-5.24	-0.009	1.56E-07	333615
Dried fruit intake	-0.003	-0.009	0.003	0.003	-0.91	-0.002	3.63E-01	329872
Bread intake	0.051	0.009	0.092	0.021	2.39	0.004	1.70E-02	330482
Oily fish intake	0.002	-0.003	0.007	0.002	0.85	0.001	3.95E-01	334293
Non-oily fish intake	-0.003	-0.007	0.001	0.002	-1.56	-0.003	1.19E-01	334248
Processed meat intake	0.006	0.001	0.011	0.003	2.19	0.004	2.89E-02	335299
Poultry intake	-0.001	-0.006	0.003	0.002	-0.54	-0.001	5.87E-01	335227
Beef intake	0.003	-0.002	0.007	0.002	1.19	0.002	2.33E-01	334529
Lamb/mutton intake	0.001	-0.002	0.005	0.002	0.77	0.001	4.40E-01	333842
Pork intake	0.000	-0.004	0.004	0.002	0.04	0.000	9.69E-01	333787
Cheese intake	0.000	-0.006	0.005	0.003	-0.16	0.000	8.69E-01	327987

Notes: The table reports the associations between the shared lead variants and diet measurements. The shared lead variants are the overlap of the lead variants of the cereal and coffee intake which are displayed in the supplementary table 5 and 6. The covariates included in the linear regression model are baseline age, gender, and the top 40 genetic components obtained from UK Biobank. Beta refers to the coefficient of the regression model. t, p, se, 95%CI_L and 95%CI_H repsepctively represent the t statistic, two-side p-value, standard error, 95% coffidence interval for coefficients. df.e refers to the degree freedom of the residuals of the regression model. r refers to the correlation coefficient.

Supplementary Table 8. Results of the modified mendelian randomization analysis.

Panel A. Association between PRS for TGMV and TGMV.								
PRS	beta	95%CI_l	95%CI_h	se	t	r	p_one-tailed	df.e
TGMV PRS	1.90E-04	1.56E-04	2.24E-04	1.74E-05	10.951	0.097	4.39E-28	12736
TGMV/Cereal0.05 PRS	1.80E-04	1.45E-04	2.14E-04	1.77E-05	10.117	0.089	2.87E-24	12736
TGMV/Cereal0.10 PRS	1.73E-04	1.37E-04	2.08E-04	1.82E-05	9.488	0.084	1.39E-21	12736
TGMV/Cereal0.15 PRS	1.68E-04	1.31E-04	2.04E-04	1.86E-05	9.003	0.080	1.25E-19	12736
TGMV/Cereal0.20 PRS	1.67E-04	1.30E-04	2.05E-04	1.91E-05	8.744	0.077	1.27E-18	12736
TGMV/Cereal0.25 PRS	1.66E-04	1.28E-04	2.05E-04	1.98E-05	8.424	0.074	2.01E-17	12736
TGMV/Cereal0.30 PRS	1.69E-04	1.29E-04	2.09E-04	2.04E-05	8.260	0.073	8.01E-17	12736
TGMV/Cereal0.35 PRS	1.63E-04	1.21E-04	2.04E-04	2.11E-05	7.725	0.068	6.00E-15	12736
TGMV/Cereal0.40 PRS	1.74E-04	1.32E-04	2.17E-04	2.18E-05	7.984	0.071	7.66E-16	12736
TGMV/Cereal0.45 PRS	1.74E-04	1.30E-04	2.19E-04	2.26E-05	7.714	0.068	6.53E-15	12736
TGMV/Cereal0.50 PRS	1.83E-04	1.37E-04	2.29E-04	2.36E-05	7.763	0.069	4.46E-15	12736
TGMV/Coffee0.05 PRS	1.78E-04	1.43E-04	2.13E-04	1.78E-05	10.036	0.089	6.46E-24	12736
TGMV/Coffee0.10 PRS	1.78E-04	1.42E-04	2.14E-04	1.81E-05	9.820	0.087	5.58E-23	12736
TGMV/Coffee0.15 PRS	1.70E-04	1.34E-04	2.07E-04	1.86E-05	9.155	0.081	3.11E-20	12736
TGMV/Coffee0.20 PRS	1.78E-04	1.40E-04	2.15E-04	1.90E-05	9.332	0.082	6.06E-21	12736
TGMV/Coffee0.25 PRS	1.78E-04	1.40E-04	2.17E-04	1.96E-05	9.085	0.080	5.96E-20	12736
TGMV/Coffee0.30 PRS	1.76E-04	1.36E-04	2.16E-04	2.02E-05	8.708	0.077	1.74E-18	12736
TGMV/Coffee0.35 PRS	1.70E-04	1.29E-04	2.11E-04	2.09E-05	8.139	0.072	2.18E-16	12736
TGMV/Coffee0.40 PRS	1.67E-04	1.25E-04	2.10E-04	2.17E-05	7.721	0.068	6.21E-15	12736
TGMV/Coffee0.45 PRS	1.57E-04	1.13E-04	2.01E-04	2.26E-05	6.950	0.061	1.92E-12	12736
TGMV/Coffee0.50 PRS	1.57E-04	1.11E-04	2.03E-04	2.35E-05	6.678	0.059	1.26E-11	12736

Panel B. Association between PRS for cereal intake and cereal intake.								
PRS	beta	95%CI_l	95%CI_h	se	t	r	p_one-tailed	df.e
Cereal PRS	3.37E-05	2.65E-05	4.09E-05	3.67E-06	9.197	0.081	2.13E-20	12739
Cereal/TGMV0.05 PRS	3.29E-05	2.56E-05	4.01E-05	3.72E-06	8.832	0.078	5.80E-19	12739
Cereal/TGMV0.10 PRS	3.33E-05	2.59E-05	4.08E-05	3.80E-06	8.773	0.077	9.77E-19	12739
Cereal/TGMV0.15 PRS	3.44E-05	2.68E-05	4.20E-05	3.88E-06	8.884	0.078	3.66E-19	12739
Cereal/TGMV0.20 PRS	3.44E-05	2.67E-05	4.22E-05	3.96E-06	8.688	0.077	2.08E-18	12739
Cereal/TGMV0.25 PRS	3.42E-05	2.62E-05	4.21E-05	4.06E-06	8.414	0.074	2.20E-17	12739
Cereal/TGMV0.30 PRS	3.48E-05	2.66E-05	4.30E-05	4.18E-06	8.322	0.074	4.75E-17	12739
Cereal/TGMV0.35 PRS	3.34E-05	2.50E-05	4.19E-05	4.31E-06	7.755	0.069	4.75E-15	12739
Cereal/TGMV0.40 PRS	3.15E-05	2.28E-05	4.02E-05	4.45E-06	7.082	0.063	7.45E-13	12739
Cereal/TGMV0.45 PRS	3.04E-05	2.13E-05	3.95E-05	4.64E-06	6.541	0.058	3.17E-11	12739
Cereal/TGMV0.50 PRS	3.14E-05	2.18E-05	4.10E-05	4.88E-06	6.441	0.057	6.16E-11	12739

Panel C. Association between PRS for coffee intake and coffee intake.								
PRS	beta	95%CI_l	95%CI_h	se	t	r	p_one-tailed	df.e
Coffee PRS	3.52E-05	2.70E-05	4.33E-05	4.15E-06	8.475	0.075	1.31E-17	12739
Coffee/TGMV0.05 PRS	3.21E-05	2.40E-05	4.01E-05	4.11E-06	7.801	0.069	3.30E-15	12739
Coffee/TGMV0.10 PRS	3.21E-05	2.39E-05	4.02E-05	4.16E-06	7.713	0.068	6.59E-15	12739
Coffee/TGMV0.15 PRS	3.04E-05	2.20E-05	3.87E-05	4.25E-06	7.142	0.063	4.85E-13	12739
Coffee/TGMV0.20 PRS	3.26E-05	2.41E-05	4.11E-05	4.35E-06	7.488	0.066	3.72E-14	12739
Coffee/TGMV0.25 PRS	3.44E-05	2.57E-05	4.32E-05	4.46E-06	7.723	0.068	6.12E-15	12739

Coffee/TGMV0.30 PRS	3.44E-05	2.54E-05	4.33E-05	4.58E-06	7.508	0.066	3.20E-14	12739
Coffee/TGMV0.35 PRS	3.43E-05	2.51E-05	4.35E-05	4.70E-06	7.296	0.065	1.57E-13	12739
Coffee/TGMV0.40 PRS	3.38E-05	2.42E-05	4.33E-05	4.87E-06	6.938	0.061	2.08E-12	12739
Coffee/TGMV0.45 PRS	3.39E-05	2.40E-05	4.38E-05	5.06E-06	6.702	0.059	1.07E-11	12739
Coffee/TGMV0.50 PRS	3.62E-05	2.59E-05	4.65E-05	5.23E-06	6.916	0.061	2.44E-12	12739

Panel D. Association between PRS for the intake of cereal/coffee and TGMV.

PRS	beta	95%CI_l	95%CI_h	se	t	r	p_one-tailed	df.e
Cereal PRS	2.67E-09	4.90E-10	4.85E-09	1.11E-09	2.400	0.021	8.20E-03	12736
Cereal/TGMV0.05 PRS	2.03E-09	-1.88E-10	4.24E-09	1.13E-09	1.794	0.016	3.64E-02	12736
Cereal/TGMV0.10 PRS	1.67E-09	-5.92E-10	3.93E-09	1.15E-09	1.446	0.013	7.40E-02	12736
Cereal/TGMV0.15 PRS	1.82E-09	-4.82E-10	4.13E-09	1.18E-09	1.551	0.014	6.05E-02	12736
Cereal/TGMV0.20 PRS	1.99E-09	-3.63E-10	4.35E-09	1.20E-09	1.658	0.015	4.86E-02	12736
Cereal/TGMV0.25 PRS	2.02E-09	-3.96E-10	4.44E-09	1.23E-09	1.639	0.015	5.06E-02	12736
Cereal/TGMV0.30 PRS	2.03E-09	-4.55E-10	4.52E-09	1.27E-09	1.602	0.014	5.46E-02	12736
Cereal/TGMV0.35 PRS	2.29E-09	-2.72E-10	4.85E-09	1.31E-09	1.752	0.016	3.99E-02	12736
Cereal/TGMV0.40 PRS	2.12E-09	-5.23E-10	4.77E-09	1.35E-09	1.573	0.014	5.79E-02	12736
Cereal/TGMV0.45 PRS	1.85E-09	-9.12E-10	4.60E-09	1.41E-09	1.312	0.012	9.47E-02	12736
Cereal/TGMV0.50 PRS	1.94E-09	-9.54E-10	4.84E-09	1.48E-09	1.315	0.012	9.43E-02	12736
Coffee PRS	-2.50E-09	-4.15E-09	-8.52E-10	8.42E-10	-2.973	-0.026	1.48E-03	12736
Coffee/TGMV0.05 PRS	-2.27E-09	-3.91E-09	-6.41E-10	8.33E-10	-2.730	-0.024	3.17E-03	12736
Coffee/TGMV0.10 PRS	-2.13E-09	-3.78E-09	-4.80E-10	8.43E-10	-2.529	-0.022	5.72E-03	12736
Coffee/TGMV0.15 PRS	-1.97E-09	-3.66E-09	-2.77E-10	8.62E-10	-2.281	-0.020	1.13E-02	12736
Coffee/TGMV0.20 PRS	-1.92E-09	-3.65E-09	-1.85E-10	8.83E-10	-2.170	-0.019	1.50E-02	12736
Coffee/TGMV0.25 PRS	-2.25E-09	-4.02E-09	-4.74E-10	9.04E-10	-2.485	-0.022	6.49E-03	12736
Coffee/TGMV0.30 PRS	-1.94E-09	-3.76E-09	-1.21E-10	9.28E-10	-2.091	-0.019	1.83E-02	12736
Coffee/TGMV0.35 PRS	-2.09E-09	-3.96E-09	-2.21E-10	9.54E-10	-2.192	-0.019	1.42E-02	12736
Coffee/TGMV0.40 PRS	-1.66E-09	-3.60E-09	2.71E-10	9.86E-10	-1.686	-0.015	4.59E-02	12736
Coffee/TGMV0.45 PRS	-1.87E-09	-3.88E-09	1.37E-10	1.03E-09	-1.827	-0.016	3.39E-02	12736
Coffee/TGMV0.50 PRS	-1.85E-09	-3.93E-09	2.32E-10	1.06E-09	-1.742	-0.015	4.08E-02	12736

Panel E. Association between PRS for TGMV and cereal intake.

PRS	beta	95%CI_l	95%CI_h	se	t	r	p_one-tailed	df.e
TGMV PRS	4.56E-02	-6.75E-02	1.59E-01	5.77E-02	0.790	0.007	2.15E-01	12739
TGMV/Cereal0.05 PRS	1.95E-02	-9.59E-02	1.35E-01	5.89E-02	0.331	0.003	3.70E-01	12739
TGMV/Cereal0.10 PRS	3.24E-04	-1.18E-01	1.19E-01	6.03E-02	0.005	0.000	4.98E-01	12739
TGMV/Cereal0.15 PRS	1.88E-02	-1.02E-01	1.40E-01	6.18E-02	0.304	0.003	3.81E-01	12739
TGMV/Cereal0.20 PRS	4.10E-03	-1.20E-01	1.28E-01	6.34E-02	0.065	0.001	4.74E-01	12739
TGMV/Cereal0.25 PRS	2.32E-04	-1.28E-01	1.29E-01	6.55E-02	0.004	0.000	4.99E-01	12739
TGMV/Cereal0.30 PRS	1.24E-02	-1.20E-01	1.45E-01	6.77E-02	0.183	0.002	4.27E-01	12739
TGMV/Cereal0.35 PRS	9.69E-04	-1.36E-01	1.38E-01	6.97E-02	0.014	0.000	4.94E-01	12739
TGMV/Cereal0.40 PRS	-7.60E-03	-1.49E-01	1.34E-01	7.24E-02	-0.105	-0.001	4.58E-01	12739
TGMV/Cereal0.45 PRS	-6.75E-03	-1.54E-01	1.40E-01	7.49E-02	-0.090	-0.001	4.64E-01	12739
TGMV/Cereal0.50 PRS	-1.12E-02	-1.64E-01	1.42E-01	7.80E-02	-0.143	-0.001	4.43E-01	12739

Panel F. Association between PRS for TGMV and coffee intake.

PRS	beta	95%CI_l	95%CI_h	se	t	r	p_one-tailed	df.e
TGMV PRS	-5.98E-02	-2.29E-01	1.09E-01	8.63E-02	-0.693	-0.006	2.44E-01	12739
TGMV/Coffee0.05 PRS	-3.35E-02	-2.06E-01	1.39E-01	8.82E-02	-0.380	-0.003	3.52E-01	12739
TGMV/Coffee0.10 PRS	-5.62E-02	-2.33E-01	1.20E-01	9.00E-02	-0.625	-0.006	2.66E-01	12739
TGMV/Coffee0.15 PRS	-5.01E-02	-2.31E-01	1.31E-01	9.22E-02	-0.544	-0.005	2.93E-01	12739
TGMV/Coffee0.20 PRS	-5.19E-02	-2.37E-01	1.33E-01	9.45E-02	-0.549	-0.005	2.92E-01	12739
TGMV/Coffee0.25 PRS	-4.28E-02	-2.33E-01	1.48E-01	9.72E-02	-0.440	-0.004	3.30E-01	12739
TGMV/Coffee0.30 PRS	-3.75E-02	-2.34E-01	1.59E-01	1.00E-01	-0.374	-0.003	3.54E-01	12739
TGMV/Coffee0.35 PRS	-6.02E-02	-2.63E-01	1.43E-01	1.03E-01	-0.582	-0.005	2.80E-01	12739
TGMV/Coffee0.40 PRS	-5.62E-02	-2.67E-01	1.54E-01	1.07E-01	-0.524	-0.005	3.00E-01	12739
TGMV/Coffee0.45 PRS	-6.96E-02	-2.89E-01	1.50E-01	1.12E-01	-0.623	-0.006	2.67E-01	12739
TGMV/Coffee0.50 PRS	-3.06E-02	-2.59E-01	1.98E-01	1.16E-01	-0.263	-0.002	3.96E-01	12739

Notes: The table reports the results of the modified mendelian randomization analysis. Panel A to F display the associations between specific polygenic risk scores (PRS) and phenotypes. Cereal PRS, Coffee PRS and TGMV PRS respectively represent the polygenic risk scores for cereal, coffee and total brain grey matter volume, which were generated by PRSice [1] v1.25 software using genetic variants in association with each phenotype with P value <0.05. The A/B c PRS notation represent the polygenic risk scores for A using genetic variants which were in association with A with P value <0.05 but not associated with B with P value >c. For example, TGMV/Cereal0.5 PRS refers to the polygenic risk score for total brain grey matter volume using genetic variants which were in association with TGMV with P value <0.05 but not associated with cereal intake (P value>0.5). The covariates inclued in the linear regression model are age at follow-up, gender,and the top 40 genetic components. The imaging sites (dummy variables) and total intracranial volume are also adjusted when TGMV is included in the model. Beta refers to the coefficient of the regression model. t, p, se, 95%CI_L and 95%CI_H repsepctively represent the t statistic, two-side p-value, standard error, 95% coffidence interval for coefficients. df.e refers to the degree freedom of the residuals of the regression model. r refers to the correlation coefficient.

References

[1] J. Euesden, C. M. Lewis, P. F. O'Reilly, PRSice: polygenic risk score software. Bioinformatics 31, 1466-1468 (2015).

Supplementary Table 9: GO terms for genes.

A. GO terms enriched in genes that overlap in transcription-related genes and coffee candidate genes.							
ONTOLOGY	ID	Description	GeneRatio	BgRatio	pvalue	p.adjust	qvalue
BP	GO:0001580	detection of chemical stimulus involved in sensory perception of bitter taste	3/13	29/17913	1.08E-06	1.22E-04	8.73E-05
BP	GO:0050913	sensory perception of bitter taste	3/13	34/17913	1.76E-06	1.22E-04	8.73E-05
BP	GO:0050912	detection of chemical stimulus involved in sensory perception of taste	3/13	35/17913	1.93E-06	1.22E-04	8.73E-05
BP	GO:0050909	sensory perception of taste	3/13	57/17913	8.54E-06	4.06E-04	2.90E-04
MF	GO:0033038	bitter taste receptor activity	3/12	18/16969	2.19E-07	5.70E-06	3.46E-06
MF	GO:0008527	taste receptor activity	3/12	24/16969	5.42E-07	7.05E-06	4.28E-06

B. GO terms for the transcription-related specific genes.							
ONTOLOGY	ID	Description	GeneRatio	BgRatio	pvalue	p.adjust	qvalue
BP	GO:0009150	purine ribonucleotide metabolic process	77/1423	500/17913	1.27E-08	6.93E-05	6.49E-05
BP	GO:0050808	synapse organization	62/1423	394/17913	1.54E-07	4.18E-04	3.92E-04
BP	GO:0009199	ribonucleoside triphosphate metabolic process	52/1423	319/17913	5.08E-07	6.63E-04	6.20E-04
BP	GO:0009141	nucleoside triphosphate metabolic process	54/1423	337/17913	5.39E-07	6.63E-04	6.20E-04
BP	GO:0009144	purine nucleoside triphosphate metabolic process	52/1423	321/17913	6.19E-07	6.63E-04	6.20E-04
BP	GO:0009205	purine ribonucleoside triphosphate metabolic process	51/1423	314/17913	7.30E-07	6.63E-04	6.20E-04
BP	GO:0099132	ATP hydrolysis coupled cation transmembrane transport	16/1423	54/17913	2.68E-06	2.09E-03	1.95E-03
BP	GO:0046034	ATP metabolic process	46/1423	287/17913	3.64E-06	2.48E-03	2.32E-03
BP	GO:0009167	purine ribonucleoside monophosphate metabolic process	49/1423	317/17913	5.04E-06	3.00E-03	2.81E-03
BP	GO:0009126	purine nucleoside monophosphate metabolic process	49/1423	318/17913	5.51E-06	3.00E-03	2.81E-03
BP	GO:0009161	ribonucleoside monophosphate metabolic process	50/1423	331/17913	7.81E-06	3.87E-03	3.62E-03
BP	GO:0050807	regulation of synapse organization	36/1423	218/17913	2.08E-05	9.45E-03	8.85E-03
BP	GO:0099175	regulation of postsynapse organization	21/1423	98/17913	2.30E-05	9.62E-03	9.00E-03
BP	GO:0099177	regulation of trans-synaptic signaling	55/1423	394/17913	2.90E-05	9.99E-03	9.35E-03

BP	GO:0009152	purine ribonucleotide biosynthetic process	41/1423	266/17913	3.10E-05	9.99E-03	9.35E-03
BP	GO:0050803	regulation of synapse structure or activity	36/1423	222/17913	3.11E-05	9.99E-03	9.35E-03
BP	GO:0046390	ribose phosphate biosynthetic process	43/1423	284/17913	3.12E-05	9.99E-03	9.35E-03
BP	GO:0009123	nucleoside monophosphate metabolic process	50/1423	349/17913	3.33E-05	1.01E-02	9.43E-03
BP	GO:0009260	ribonucleotide biosynthetic process	42/1423	279/17913	4.40E-05	1.26E-02	1.18E-02
BP	GO:0099173	postsynapse organization	28/1423	158/17913	4.66E-05	1.27E-02	1.19E-02
BP	GO:0050804	modulation of chemical synaptic transmission	54/1423	393/17913	5.27E-05	1.34E-02	1.25E-02
BP	GO:0045333	cellular respiration	30/1423	176/17913	5.41E-05	1.34E-02	1.25E-02
BP	GO:0051963	regulation of synapse assembly	21/1423	105/17913	6.75E-05	1.60E-02	1.49E-02
BP	GO:0010506	regulation of autophagy	45/1423	314/17913	8.02E-05	1.82E-02	1.70E-02
BP	GO:0007416	synapse assembly	29/1423	172/17913	8.74E-05	1.85E-02	1.73E-02
BP	GO:0006090	pyruvate metabolic process	26/1423	147/17913	8.85E-05	1.85E-02	1.73E-02
BP	GO:0060271	cilium assembly	47/1423	335/17913	9.50E-05	1.92E-02	1.79E-02
BP	GO:0006164	purine nucleotide biosynthetic process	41/1423	280/17913	1.01E-04	1.97E-02	1.84E-02
BP	GO:0072522	purine-containing compound biosynthetic process	42/1423	291/17913	1.16E-04	2.19E-02	2.05E-02
BP	GO:0070646	protein modification by small protein removal	40/1423	273/17913	1.21E-04	2.19E-02	2.05E-02
BP	GO:0048489	synaptic vesicle transport	23/1423	126/17913	1.36E-04	2.24E-02	2.10E-02
BP	GO:0097480	establishment of synaptic vesicle localization	23/1423	126/17913	1.36E-04	2.24E-02	2.10E-02
BP	GO:0007005	mitochondrion organization	60/1423	466/17913	1.36E-04	2.24E-02	2.10E-02
BP	GO:0099150	regulation of postsynaptic specialization assembly	6/1423	12/17913	1.51E-04	2.30E-02	2.15E-02
BP	GO:0150052	regulation of postsynapse assembly	6/1423	12/17913	1.51E-04	2.30E-02	2.15E-02
BP	GO:0010975	regulation of neuron projection development	59/1423	458/17913	1.52E-04	2.30E-02	2.15E-02
BP	GO:0044782	cilium organization	47/1423	346/17913	2.06E-04	3.03E-02	2.83E-02
BP	GO:0099003	vesicle-mediated transport in synapse	28/1423	174/17913	2.58E-04	3.46E-02	3.24E-02
BP	GO:0070296	sarcoplasmic reticulum calcium ion transport	11/1423	41/17913	2.60E-04	3.46E-02	3.24E-02
BP	GO:0031123	RNA 3'-end processing	20/1423	107/17913	2.61E-04	3.46E-02	3.24E-02
BP	GO:0051656	establishment of organelle localization	57/1423	448/17913	2.69E-04	3.46E-02	3.24E-02
BP	GO:0016241	regulation of macroautophagy	27/1423	166/17913	2.76E-04	3.46E-02	3.24E-02
BP	GO:0014808	release of sequestered calcium ion into cytosol by sarcoplasmic reticulum	10/1423	35/17913	2.82E-04	3.46E-02	3.24E-02
BP	GO:0070932	histone H3 deacetylation	7/1423	18/17913	2.86E-04	3.46E-02	3.24E-02
BP	GO:0098698	postsynaptic specialization assembly	7/1423	18/17913	2.86E-04	3.46E-02	3.24E-02

BP	GO:0071426	ribonucleoprotein complex export from nucleus	21/1423	116/17913	2.93E-04	3.46E-02	3.24E-02
BP	GO:0022406	membrane docking	25/1423	150/17913	3.13E-04	3.46E-02	3.24E-02
BP	GO:0051168	nuclear export	28/1423	176/17913	3.13E-04	3.46E-02	3.24E-02
BP	GO:0051169	nuclear transport	42/1423	305/17913	3.27E-04	3.46E-02	3.24E-02
BP	GO:0051208	sequestering of calcium ion	21/1423	117/17913	3.31E-04	3.46E-02	3.24E-02
BP	GO:0071166	ribonucleoprotein complex localization	21/1423	117/17913	3.31E-04	3.46E-02	3.24E-02
BP	GO:0034248	regulation of cellular amide metabolic process	57/1423	452/17913	3.38E-04	3.46E-02	3.24E-02
BP	GO:0097553	calcium ion transmembrane import into cytosol	22/1423	126/17913	3.61E-04	3.46E-02	3.24E-02
BP	GO:0015682	ferric iron transport	10/1423	36/17913	3.62E-04	3.46E-02	3.24E-02
BP	GO:0033572	transferrin transport	10/1423	36/17913	3.62E-04	3.46E-02	3.24E-02
BP	GO:0072512	trivalent inorganic cation transport	10/1423	36/17913	3.62E-04	3.46E-02	3.24E-02
BP	GO:1903514	release of sequestered calcium ion into cytosol by endoplasmic reticulum	10/1423	36/17913	3.62E-04	3.46E-02	3.24E-02
BP	GO:1901293	nucleoside phosphate biosynthetic process	48/1423	365/17913	3.78E-04	3.52E-02	3.30E-02
BP	GO:0016579	protein deubiquitination	37/1423	260/17913	3.82E-04	3.52E-02	3.30E-02
BP	GO:0097479	synaptic vesicle localization	23/1423	137/17913	4.80E-04	4.15E-02	3.88E-02
BP	GO:0006611	protein export from nucleus	26/1423	163/17913	4.84E-04	4.15E-02	3.88E-02
BP	GO:0051209	release of sequestered calcium ion into cytosol	20/1423	112/17913	4.86E-04	4.15E-02	3.88E-02
BP	GO:0051283	negative regulation of sequestering of calcium ion	20/1423	112/17913	4.86E-04	4.15E-02	3.88E-02
BP	GO:0015991	ATP hydrolysis coupled proton transport	8/1423	25/17913	4.93E-04	4.15E-02	3.88E-02
BP	GO:0010880	regulation of release of sequestered calcium ion into cytosol by sarcoplasmic reticulum	9/1423	31/17913	4.97E-04	4.15E-02	3.88E-02
BP	GO:0051028	mRNA transport	24/1423	146/17913	5.03E-04	4.15E-02	3.88E-02
BP	GO:0006913	nucleocytoplasmic transport	41/1423	302/17913	5.14E-04	4.17E-02	3.91E-02
BP	GO:0009201	ribonucleoside triphosphate biosynthetic process	26/1423	164/17913	5.33E-04	4.27E-02	3.99E-02
BP	GO:0055117	regulation of cardiac muscle contraction	16/1423	81/17913	5.54E-04	4.29E-02	4.01E-02
BP	GO:0021548	pons development	5/1423	10/17913	5.64E-04	4.29E-02	4.01E-02
BP	GO:0060456	positive regulation of digestive system process	5/1423	10/17913	5.64E-04	4.29E-02	4.01E-02
BP	GO:0031124	mRNA 3'-end processing	17/1423	89/17913	5.67E-04	4.29E-02	4.01E-02
BP	GO:0006405	RNA export from nucleus	21/1423	122/17913	5.91E-04	4.41E-02	4.12E-02
BP	GO:0031503	protein-containing complex localization	34/1423	238/17913	6.01E-04	4.41E-02	4.12E-02
BP	GO:1903902	positive regulation of viral life cycle	13/1423	59/17913	6.07E-04	4.41E-02	4.12E-02

BP	GO:0051282	regulation of sequestering of calcium ion	20/1423	114/17913	6.15E-04	4.41E-02	4.13E-02
BP	GO:0023061	signal release	54/1423	433/17913	6.25E-04	4.42E-02	4.14E-02
BP	GO:0009142	nucleoside triphosphate biosynthetic process	27/1423	175/17913	6.50E-04	4.48E-02	4.19E-02
BP	GO:1901292	nucleoside phosphate catabolic process	27/1423	175/17913	6.50E-04	4.48E-02	4.19E-02
BP	GO:0015988	energy coupled proton transmembrane transport, against electrochemical gradient	8/1423	26/17913	6.63E-04	4.51E-02	4.22E-02
BP	GO:0050657	nucleic acid transport	27/1423	176/17913	7.10E-04	4.63E-02	4.34E-02
BP	GO:0050658	RNA transport	27/1423	176/17913	7.10E-04	4.63E-02	4.34E-02
BP	GO:0006406	mRNA export from nucleus	19/1423	107/17913	7.15E-04	4.63E-02	4.34E-02
BP	GO:0071427	mRNA-containing ribonucleoprotein complex export from nucleus	19/1423	107/17913	7.15E-04	4.63E-02	4.34E-02
BP	GO:0009206	purine ribonucleoside triphosphate biosynthetic process	25/1423	159/17913	7.64E-04	4.85E-02	4.54E-02
BP	GO:0009135	purine nucleoside diphosphate metabolic process	22/1423	133/17913	7.75E-04	4.85E-02	4.54E-02
BP	GO:0009179	purine ribonucleoside diphosphate metabolic process	22/1423	133/17913	7.75E-04	4.85E-02	4.54E-02
CC	GO:0033267	axon part	54/1472	354/18678	2.03E-06	1.31E-03	1.08E-03
CC	GO:0098978	glutamatergic synapse	51/1472	352/18678	1.67E-05	4.36E-03	3.59E-03
CC	GO:0097060	synaptic membrane	55/1472	395/18678	2.52E-05	4.36E-03	3.59E-03
CC	GO:0043025	neuronal cell body	61/1472	454/18678	2.79E-05	4.36E-03	3.59E-03
CC	GO:0098793	presynapse	59/1472	438/18678	3.49E-05	4.36E-03	3.59E-03
CC	GO:0150034	distal axon	40/1472	262/18678	4.05E-05	4.36E-03	3.59E-03
CC	GO:0045211	postsynaptic membrane	43/1472	299/18678	8.88E-05	8.19E-03	6.75E-03
CC	GO:0016469	proton-transporting two-sector ATPase complex	12/1472	44/18678	1.07E-04	8.68E-03	7.16E-03
CC	GO:0043204	perikaryon	23/1472	127/18678	1.37E-04	9.71E-03	8.01E-03
CC	GO:0044306	neuron projection terminus	22/1472	120/18678	1.57E-04	9.71E-03	8.01E-03
CC	GO:0098798	mitochondrial protein complex	35/1472	233/18678	1.65E-04	9.71E-03	8.01E-03
CC	GO:0048786	presynaptic active zone	14/1472	60/18678	1.85E-04	9.94E-03	8.20E-03
CC	GO:0036464	cytoplasmic ribonucleoprotein granule	28/1472	176/18678	2.76E-04	1.37E-02	1.13E-02
CC	GO:0043679	axon terminus	19/1472	101/18678	3.06E-04	1.38E-02	1.14E-02
CC	GO:0034399	nuclear periphery	22/1472	126/18678	3.24E-04	1.38E-02	1.14E-02
CC	GO:0000922	spindle pole	25/1472	152/18678	3.42E-04	1.38E-02	1.14E-02

CC	GO:0035770	ribonucleoprotein granule	29/1472	188/18678	3.69E-04	1.40E-02	1.16E-02
CC	GO:0016363	nuclear matrix	19/1472	106/18678	5.76E-04	2.07E-02	1.71E-02
CC	GO:0098800	inner mitochondrial membrane protein complex	20/1472	115/18678	6.26E-04	2.13E-02	1.75E-02
CC	GO:0005681	spliceosomal complex	25/1472	160/18678	7.50E-04	2.42E-02	2.00E-02
CC	GO:0010494	cytoplasmic stress granule	12/1472	55/18678	9.96E-04	3.02E-02	2.49E-02
CC	GO:0098984	neuron to neuron synapse	41/1472	315/18678	1.03E-03	3.02E-02	2.49E-02
CC	GO:0099240	intrinsic component of synaptic membrane	25/1472	164/18678	1.08E-03	3.03E-02	2.50E-02
CC	GO:0001725	stress fiber	12/1472	56/18678	1.18E-03	3.05E-02	2.51E-02
CC	GO:0097517	contractile actin filament bundle	12/1472	56/18678	1.18E-03	3.05E-02	2.51E-02
CC	GO:0070469	respiratory chain	16/1472	88/18678	1.30E-03	3.21E-02	2.65E-02
CC	GO:0005747	mitochondrial respiratory chain complex I	11/1472	50/18678	1.49E-03	3.21E-02	2.65E-02
CC	GO:0030964	NADH dehydrogenase complex	11/1472	50/18678	1.49E-03	3.21E-02	2.65E-02
CC	GO:0045271	respiratory chain complex I	11/1472	50/18678	1.49E-03	3.21E-02	2.65E-02
CC	GO:0060198	clathrin-sculpted vesicle	5/1472	12/18678	1.49E-03	3.21E-02	2.65E-02
CC	GO:0042734	presynaptic membrane	22/1472	141/18678	1.54E-03	3.21E-02	2.65E-02
CC	GO:0043197	dendritic spine	22/1472	142/18678	1.69E-03	3.41E-02	2.81E-02
CC	GO:0032432	actin filament bundle	12/1472	59/18678	1.90E-03	3.62E-02	2.98E-02
CC	GO:0005759	mitochondrial matrix	53/1472	448/18678	1.93E-03	3.62E-02	2.98E-02
CC	GO:0042641	actomyosin	13/1472	67/18678	1.96E-03	3.62E-02	2.98E-02
CC	GO:0044309	neuron spine	22/1472	145/18678	2.22E-03	3.91E-02	3.23E-02
CC	GO:0005635	nuclear envelope	50/1472	420/18678	2.24E-03	3.91E-02	3.23E-02
CC	GO:0098936	intrinsic component of postsynaptic membrane	19/1472	120/18678	2.63E-03	4.37E-02	3.60E-02
CC	GO:0031965	nuclear membrane	36/1472	281/18678	2.64E-03	4.37E-02	3.60E-02
CC	GO:0005743	mitochondrial inner membrane	52/1472	445/18678	2.75E-03	4.37E-02	3.60E-02
CC	GO:0099634	postsynaptic specialization membrane	15/1472	86/18678	2.77E-03	4.37E-02	3.60E-02
CC	GO:0032279	asymmetric synapse	37/1472	294/18678	3.15E-03	4.64E-02	3.83E-02
CC	GO:0016607	nuclear speck	46/1472	386/18678	3.21E-03	4.64E-02	3.83E-02
CC	GO:0005746	mitochondrial respiratory chain	14/1472	79/18678	3.25E-03	4.64E-02	3.83E-02
CC	GO:0000159	protein phosphatase type 2A complex	5/1472	14/18678	3.31E-03	4.64E-02	3.83E-02
CC	GO:0033178	proton-transporting two-sector ATPase complex, catalytic domain	5/1472	14/18678	3.31E-03	4.64E-02	3.83E-02
CC	GO:0099572	postsynaptic specialization	39/1472	316/18678	3.49E-03	4.76E-02	3.92E-02

CC	GO:0005874	microtubule	43/1472	357/18678	3.53E-03	4.76E-02	3.92E-02

C. GO terms for the candidate genes of the coffee intake.

ONTOLOGY	ID	Description	GeneRatio	BgRatio	pvalue	p.adjust	qvalue
BP	GO:0071248	cellular response to metal ion	9/161	167/17913	2.04E-05	2.70E-02	2.61E-02
BP	GO:0071294	cellular response to zinc ion	4/161	19/17913	2.19E-05	2.70E-02	2.61E-02
BP	GO:0071241	cellular response to inorganic substance	9/161	194/17913	6.60E-05	4.23E-02	4.08E-02
BP	GO:0006706	steroid catabolic process	4/161	25/17913	6.86E-05	4.23E-02	4.08E-02
MF	GO:0033038	bitter taste receptor activity	4/161	18/16969	2.15E-05	7.41E-03	7.28E-03
MF	GO:0008527	taste receptor activity	4/161	24/16969	7.15E-05	1.23E-02	1.21E-02

Notes: The table reports the GO terms for genes, determined by over-represented analysis via ClusterProfile [1] R package. Panel A shows GO terms enriched in genes that overlap in transcription-related genes and coffee candidate genes. Panel B displays GO terms enriched in genes which are transcription-related but not coffee candidate genes. Panel C displays GO terms enriched in genes which are coffee candidate genes but not transcription-related.

References

[1] G. Yu, L.-G. Wang, Y. Han, Q.-Y. He, clusterProfiler: an R package for comparing biological themes among gene clusters. Omics: a journal of integrative biology 16, 284-287 (2012).

Supplementary Table 10. Associations between the share lead variants and lifestyle phenotypes.

Panel A. Association between the C allele of rs2504706 and lifestyle phenotypes.

Variable	beta	95%CI_L	95%CI_H	se	t	r	p	df.e
Time spent watching television (TV)	-0.012	-0.021	-0.003	0.005	-2.65	-0.005	8.03E-03	332902
Usual walking pace	0.004	0.001	0.008	0.002	2.43	0.004	1.51E-02	333424
Summed MET minutes per week for all activity	-12.803	-29.777	4.171	8.660	-1.48	-0.003	1.39E-01	271649
MET minutes per week for moderate activity	-5.049	-12.741	2.644	3.925	-1.29	-0.002	1.98E-01	271649
MET minutes per week for vigorous activity	-0.026	-7.492	7.440	3.809	-0.01	0.000	9.95E-01	271649
MET minutes per week for walking	-7.728	-14.566	-0.890	3.489	-2.22	-0.004	2.68E-02	271649
Sleep duration	0.007	0.000	0.013	0.003	2.09	0.004	3.62E-02	333487
Getting up in morning	-0.010	-0.014	-0.006	0.002	-4.64	-0.008	3.56E-06	334568
Morning/evening person (chronotype)	0.012	0.007	0.018	0.003	4.39	0.008	1.12E-05	299433
Nap during day	0.003	0.000	0.006	0.002	1.73	0.003	8.33E-02	335137
Sleeplessness / insomnia	-0.001	-0.005	0.003	0.002	-0.64	-0.001	5.19E-01	335030
Daytime dozing / sleeping (narcolepsy)	0.001	-0.002	0.003	0.001	0.36	0.001	7.22E-01	334152
Ever smoked	0.001	-0.002	0.003	0.001	0.44	0.001	6.59E-01	334140
Number of cigarettes currently smoked daily (current cigarette smokers)	-0.041	-0.219	0.138	0.091	-0.44	-0.003	6.57E-01	23024
Alcohol intake frequency	-0.007	-0.015	0.002	0.004	-1.56	-0.003	1.19E-01	335029
Average intake of alcoholic units per week	-0.057	-0.163	0.049	0.054	-1.06	-0.002	2.90E-01	331227

Panel B. Association between the C allele of rs4410790 and lifestyle phenotypes.

Variable	beta	95%CI_L	95%CI_H	se	t	r	p	df.e
Time spent watching television (TV)	0.012	0.004	0.020	0.004	3.02	0.005	2.54E-03	332793
Usual walking pace	-0.001	-0.003	0.002	0.002	-0.33	-0.001	7.41E-01	333312
Summed MET minutes per week for all activity	0.548	-14.417	15.514	7.635	0.07	0.000	9.43E-01	271577
MET minutes per week for moderate activity	-0.914	-7.695	5.866	3.460	-0.26	-0.001	7.92E-01	271577
MET minutes per week for vigorous activity	-1.891	-8.475	4.694	3.360	-0.56	-0.001	5.74E-01	271577
MET minutes per week for walking	3.353	-2.675	9.381	3.076	1.09	0.002	2.76E-01	271577
Sleep duration	-0.001	-0.007	0.004	0.003	-0.51	-0.001	6.11E-01	333372
Getting up in morning	-0.008	-0.012	-0.004	0.002	-4.11	-0.007	3.89E-05	334451
Morning/evening person (chronotype)	0.001	-0.003	0.006	0.002	0.59	0.001	5.55E-01	299344
Nap during day	-0.003	-0.006	-0.001	0.001	-2.31	-0.004	2.10E-02	335022
Sleeplessness / insomnia	0.001	-0.003	0.004	0.002	0.43	0.001	6.65E-01	334913
Daytime dozing / sleeping (narcolepsy)	-0.004	-0.007	-0.002	0.001	-3.60	-0.006	3.17E-04	334034
Ever smoked	-0.005	-0.007	-0.002	0.001	-3.87	-0.007	1.11E-04	334030
Number of cigarettes currently smoked daily (current cigarette smokers)	0.008	-0.148	0.163	0.079	0.09	0.001	9.25E-01	23040
Alcohol intake frequency	0.001	-0.007	0.008	0.004	0.19	0.000	8.50E-01	334915
Average intake of alcoholic units per week	0.085	-0.008	0.179	0.048	1.79	0.003	7.27E-02	331114

Panel C. Association between the T allele of rs2472297 and lifestyle phenotypes.

Variable	beta	95%CI_L	95%CI_H	se	t	r	p	df.e
Time spent watching television (TV)	0.003	-0.005	0.012	0.004	0.78	0.001	4.32E-01	333404
Usual walking pace	-0.002	-0.005	0.001	0.002	-1.17	-0.002	2.43E-01	333933
Summed MET minutes per week for all activity	-9.714	-25.945	6.517	8.281	-1.17	-0.002	2.41E-01	272093

MET minutes per week for moderate activity	-3.288	-10.643	4.067	3.753	-0.88	-0.002	3.81E-01	272093
MET minutes per week for vigorous activity	-4.525	-11.666	2.616	3.643	-1.24	-0.002	2.14E-01	272093
MET minutes per week for walking	-1.901	-8.438	4.636	3.335	-0.57	-0.001	5.69E-01	272093
Sleep duration	0.000	-0.005	0.006	0.003	0.14	0.000	8.86E-01	333993
Getting up in morning	-0.003	-0.007	0.001	0.002	-1.24	-0.002	2.15E-01	335072
Morning/evening person (chronotype)	0.002	-0.004	0.007	0.003	0.56	0.001	5.76E-01	299893
Nap during day	-0.005	-0.008	-0.002	0.002	-3.23	-0.006	1.22E-03	335644
Sleeplessness / insomnia	0.001	-0.003	0.005	0.002	0.42	0.001	6.72E-01	335536
Daytime dozing / sleeping (narcolepsy)	-0.006	-0.008	-0.003	0.001	-4.28	-0.007	1.91E-05	334656
Ever smoked	-0.002	-0.005	0.001	0.001	-1.56	-0.003	1.18E-01	334645
Number of cigarettes currently smoked daily (current cigarette smokers)	0.036	-0.133	0.205	0.086	0.42	0.003	6.77E-01	23068
Alcohol intake frequency	0.013	0.005	0.021	0.004	3.28	0.006	1.02E-03	335536
Average intake of alcoholic units per week	0.161	0.060	0.262	0.052	3.12	0.005	1.82E-03	331734

Notes: The table reports the associations between the shared lead variants and lifestyle phenotypes. The shared lead variants are the overlap of the lead variants of the cereal and coffee intake which are displayed in the Supplementary Table 5 and 6. The covariates included in the linear regression model are baseline age, gender, and the top 40 genetic components obtained from UK Biobank. Beta refers to the coefficient of the regression model. t, p, se, 95%CI_L and 95%CI_H repsepctively represent the t statistic, two-side p-value, standard error, 95% coffidence interval for coefficients. df.e refers to the degree freedom of the residuals of the regression model. r refers to the correlation coefficient.

Supplementary Table 11. Associations between the cereal/coffee intake and lifestyle phenotypes.

Panel A. Associations between the cereal intake and lifestyle phenotypes.								
Variable	beta	95%CI_l	95%CI_h	se	t	r	p	df.e
Time spent watching television (TV)	-0.034	-0.036	-0.032	0.001	-33.21	-0.057	2.11E-241	333481
Usual walking pace	0.015	0.014	0.015	0.000	38.53	0.067	0.00E+00	333879
Summed MET minutes per week for all activity	16.055	12.339	19.772	1.896	8.47	0.016	2.52E-17	272389
MET minutes per week for moderate activity	3.943	2.259	5.628	0.859	4.59	0.009	4.46E-06	272389
MET minutes per week for vigorous activity	13.333	11.700	14.966	0.833	16.00	0.031	1.36E-57	272389
MET minutes per week for walking	-1.221	-2.718	0.277	0.764	-1.60	-0.003	1.10E-01	272389
Sleep duration	0.012	0.011	0.013	0.001	17.54	0.030	7.62E-69	334015
Getting up in morning	0.004	0.003	0.005	0.000	8.22	0.014	2.10E-16	335031
Morning/evening person (chronotype)	-0.013	-0.015	-0.012	0.001	-21.70	-0.040	2.57E-104	299974
Nap during day	-0.001	-0.002	0.000	0.000	-3.30	-0.006	9.75E-04	335581
Sleeplessness / insomnia	-0.007	-0.008	-0.006	0.000	-15.23	-0.026	2.24E-52	335475
Daytime dozing / sleeping (narcolepsy)	0.002	0.001	0.002	0.000	5.93	0.010	3.05E-09	334626
Ever smoked	-0.016	-0.016	-0.015	0.000	-50.78	-0.087	0.00E+00	334593
Number of cigarettes currently smoked daily (current cigarette smokers)	-0.380	-0.418	-0.342	0.019	-19.77	-0.129	2.65E-86	23037
Alcohol intake frequency	-0.054	-0.056	-0.052	0.001	-58.81	-0.101	0.00E+00	335482
Average intake of alcoholic units per week	-1.245	-1.268	-1.222	0.012	-106.90	-0.182	0.00E+00	331798

Panel B. Associations between the coffee intake and lifestyle phenotypes.								
Variable	beta	95%CI_l	95%CI_h	se	t	r	p	df.e
Time spent watching television (TV)	0.003	0.001	0.006	0.001	2.35	0.004	1.89E-02	332848
Usual walking pace	-0.003	-0.004	-0.002	0.001	-6.24	-0.011	4.30E-10	333261
Summed MET minutes per week for all activity	-15.916	-21.186	-10.647	2.689	-5.92	-0.011	3.23E-09	271875
MET minutes per week for moderate activity	-8.399	-10.789	-6.010	1.219	-6.89	-0.013	5.63E-12	271875
MET minutes per week for vigorous activity	-5.675	-7.991	-3.359	1.182	-4.80	-0.009	1.56E-06	271875
MET minutes per week for walking	-1.842	-3.967	0.282	1.084	-1.70	-0.003	8.92E-02	271875
Sleep duration	-0.016	-0.018	-0.014	0.001	-16.93	-0.029	2.67E-64	333400
Getting up in morning	0.000	-0.002	0.001	0.001	-0.48	-0.001	6.30E-01	334401
Morning/evening person (chronotype)	0.015	0.014	0.017	0.001	17.59	0.032	3.05E-69	299387
Nap during day	-0.001	-0.002	0.000	0.001	-1.66	-0.003	9.62E-02	334951
Sleeplessness / insomnia	-0.003	-0.004	-0.001	0.001	-3.96	-0.007	7.47E-05	334845
Daytime dozing / sleeping (narcolepsy)	0.000	-0.001	0.001	0.000	-0.70	-0.001	4.86E-01	333999
Ever smoked	0.018	0.017	0.019	0.000	40.80	0.070	0.00E+00	333973
Number of cigarettes currently smoked daily (current cigarette smokers)	0.282	0.241	0.322	0.021	13.59	0.090	6.69E-42	22727
Alcohol intake frequency	0.038	0.035	0.040	0.001	28.94	0.050	5.77E-184	334856
Average intake of alcoholic units per week	0.326	0.293	0.359	0.017	19.48	0.034	1.68E-84	331168

Notes: The table reports the associations Associations between the cereal/coffee intake and lifestyle phenotypes. The covariates included in the linear regression model are baseline age, gender. Beta refers to the coefficient of the regression model. t, p, se, 95%CI_L and 95%CI_H repsepctively represent the t statistic, two-side p-value, standard error, 95% coffidence interval for coefficients. df.e refers to the degree freedom of the residuals of the regression model. r refers to the correlation coefficient.

Supplementary Table 12. Significant mediation models of SNPs, lifestyle phenotypes, and the intake of cereal and coffee.

Panel A. Description of significant mediation models.

No.	X	Y	Z
1	rs4410790_C	Cereal intake	Getting up in morning
2	rs4410790_C	Cereal intake	Daytime dozing / sleeping
3	rs2472297_T	Cereal intake	Daytime dozing / sleeping
4	rs2472297_T	Cereal intake	Alcohol intake frequency
5	rs2472297_T	Coffee intake	Alcohol intake frequency

Panel B. Statistics of significant mediation models.

	Beta	95%CI_l	95%CI_h	se	T	P	R
No.				X~Z			
1	-0.008	-0.012	-0.004	0.002	-4.188	2.82E-05	-0.007
2	-0.004	-0.007	-0.002	0.001	-3.615	3.00E-04	-0.006
3	-0.006	-0.008	-0.003	0.001	-4.240	2.23E-05	-0.007
4	0.013	0.005	0.021	0.004	3.243	1.18E-03	0.006
5	0.014	0.006	0.022	0.004	3.418	6.32E-04	0.006
No.				X~Y			
1	-0.038	-0.052	-0.024	0.007	-5.447	5.13E-08	-0.009
2	-0.038	-0.051	-0.024	0.007	-5.395	6.87E-08	-0.009
3	-0.059	-0.073	-0.044	0.008	-7.761	8.48E-15	-0.013
4	-0.060	-0.074	-0.045	0.008	-7.891	3.01E-15	-0.014
5	0.142	0.131	0.152	0.005	26.544	4.36E-155	0.046
No.				Y~Z/X			
1	0.004	0.003	0.005	0.000	7.588	3.25E-14	0.013
2	0.002	0.001	0.003	0.000	6.288	3.23E-10	0.011
3	0.002	0.001	0.003	0.000	6.241	4.36E-10	0.011
4	-0.054	-0.056	-0.052	0.001	-59.152	0.00E+00	-0.102
5	0.037	0.034	0.040	0.001	28.421	1.79E-177	0.049
No.				X~Z/Y			
1	-0.008	-0.012	-0.004	0.002	-4.116	3.85E-05	-0.007
2	-0.004	-0.007	-0.002	0.001	-3.556	3.76E-04	-0.006
3	-0.006	-0.008	-0.003	0.001	-4.156	3.24E-05	-0.007
4	0.010	0.002	0.018	0.004	2.453	1.42E-02	0.004
5	0.009	0.001	0.016	0.004	2.114	3.45E-02	0.004
No.				Z~Y/X			
1	0.048	0.036	0.060	0.006	7.588	3.25E-14	0.013
2	0.061	0.042	0.080	0.010	6.288	3.23E-10	0.011
3	0.060	0.041	0.079	0.010	6.241	4.36E-10	0.011
4	-0.191	-0.197	-0.185	0.003	-59.152	0.00E+00	-0.102
5	0.065	0.061	0.070	0.002	28.421	1.79E-177	0.049
No.				X~Y/Z			
1	-0.038	-0.051	-0.024	0.007	-5.392	6.96E-08	-0.009
2	-0.037	-0.051	-0.024	0.007	-5.355	8.54E-08	-0.009
3	-0.058	-0.073	-0.043	0.008	-7.715	1.21E-14	-0.013
4	-0.057	-0.072	-0.042	0.008	-7.600	2.97E-14	-0.013
5	0.141	0.131	0.151	0.005	26.408	1.61E-153	0.046

Panle C. Model selection.								
	X~Y~Z			X~Z~Y			Model comparation	
No.	Bootsrap P value	Theoretic P value	Proportion of Mediation (PM)	Bootsrap P value	Theoretic P value	Proportion of Mediation (PM)	Excess PM of X~Y~Z than X~Z~Y	P value of Excess PM
1	0	1.55E-05	1.71%	0	2.28E-04	1.01%	0.70%	0.38
2	0	1.07E-04	1.63%	0	1.89E-03	0.73%	0.90%	0.214
3	0	2.82E-06	1.98%	0	4.55E-04	0.59%	1.39%	0.018
4	0	5.47E-15	24.75%	0	1.54E-03	4.18%	20.57%	0.002
5	0	1.37E-82	38.14%	0	8.85E-04	0.63%	37.51%	0

Notes: The table reports significant mediation models of SNPs, lifestyle phenotypes and the intake of cereal and coffee. Panel A displays significant mediation models which were tested using Baron and Kenny's (1986) [1] causal steps approach and corrected for mutiple testing using Bonferroni procedure. In the analysis, all possible mediation models between SNPs, lifestyle phenotypes and cereal/coffee intake were assessed. The covariates included were baseline age, gender and the top 40 genetic components obtained from UK Biobank. SNPs refers to the shared lead variants of the cereal and coffee intake. Panel B shows the statistics of the significant mediation models. A~B means that A is the independent variable and B is the dependent variable in the regression model. A~B/C means that A is the independent variable and B is the dependent variable in the regression model while C is added to the model as covariates. For example, in the first mediation model, X is the C-allele of rs2504706, Y is the cereal intake, and Z is the 'Getting up in morning'. Here, in the X~Z model, we tested the association between the C-allele of rs2504706 and 'Getting up in morning'. Besides, in the X~Z/Y model, we tested the association between the C-allele of rs2504706 and 'Getting up in morning' with the cereal intake adjusted. Beta refers to the coefficient of the regression model. T, P, se, 95%CI_L and 95%CI_H repsepctively represent the t statistic, two-side p-value, standard error, 95% coffidence interval for coefficients. R refers to the correlation coefficient. Panel C shows the comparation between possible directions of the mediation models. X~Y~Z means that X is the independent variable, Y is the mediation variable and Z is the dependent variable. A complete mediation model X~Y~Z consists of four parts: X~Z, X~Y, Y~Z/X and X~Z/Y. The bootstrap P value of the mediation model was obtained from 1000 boostrap procedure. Theoretic P value was computed using normal distribution. Proportion of Mediation (PM) was calculated by the regression coeffecients of the X~Z and X~Z/Y model. Excess PM of X~Y~Z than X~Z~Y is the difference bwteen the PM of X~Y~Z and the X~Z~Y. The P value of Excess PM is obtained from the 1000 bootstrap procedure.

References

[1] R. M. Baron, D. A. Kenny, The moderator–mediator variable distinction in social psychological research: Conceptual, strategic, and statistical considerations. Journal of personality and social psychology 51, 1173 (1986).

Supplementary Table 13. Associations between the shared lead variants and body/blood fat levels.

Panel A. Association between the C allele of rs2504706 and body/blood fat levels.

Variable	beta	95%CI_L	95%CI_H	se	t	r	p	df.e
Body mass index (BMI)	-0.080	-0.107	-0.053	0.014	-5.88	-0.010	4.21E-09	334176
Cholesterol	-0.004	-0.011	0.002	0.003	-1.22	-0.002	2.21E-01	319620
HDL cholesterol	-0.001	-0.003	0.001	0.001	-1.08	-0.002	2.81E-01	292598
LDL direct	-0.001	-0.006	0.004	0.003	-0.47	-0.001	6.38E-01	319014
Triglycerides	-0.002	-0.008	0.003	0.003	-0.78	-0.001	4.36E-01	319357

Panel B. Association between the C allele of rs4410790 and body/blood fat levels.

Variable	beta	95%CI_L	95%CI_H	se	t	r	p	df.e
Body mass index (BMI)	0.046	0.022	0.070	0.012	3.83	0.007	1.30E-04	334063
Cholesterol	0.005	-0.001	0.011	0.003	1.64	0.003	1.02E-01	319519
HDL cholesterol	-0.005	-0.007	-0.003	0.001	-4.93	-0.009	8.07E-07	292504
LDL direct	0.005	0.000	0.009	0.002	2.15	0.004	3.17E-02	318915
Triglycerides	0.015	0.010	0.021	0.003	5.94	0.011	2.84E-09	319256

Panel C. Association between the T allele of rs2472297 and body/blood fat levels.

Variable	beta	95%CI_L	95%CI_H	se	t	r	p	df.e
Body mass index (BMI)	0.070	0.044	0.095	0.013	5.34	0.009	9.43E-08	334680
Cholesterol	0.014	0.008	0.020	0.003	4.38	0.008	1.19E-05	320107
HDL cholesterol	0.003	0.001	0.005	0.001	2.64	0.005	8.32E-03	293041
LDL direct	0.010	0.006	0.015	0.002	4.23	0.007	2.39E-05	319502
Triglycerides	-0.005	-0.011	0.000	0.003	-1.88	-0.003	5.98E-02	319843

Notes: The table reports the associations between the shared lead variants and body/blood fat levels. The shared lead variants are the overlap of the lead variants of the cereal and coffee intake which are displayed in the Supplementary Table 5 and 6. The covariates included in the linear regression model are baseline age, gender, and the top 40 genetic components obtained from UK Biobank. Beta refers to the coefficient of the regression model. t, p, se, 95%CI_L and 95%CI_H repsepctively represent the t statistic, two-side p-value, standard error, 95% coffidence interval for coefficients. df.e refers to the degree freedom of the residuals of the regression model. r refers to the correlation coefficient.

Supplementary Table 14. Associations between the cereal/coffee intake and body/blood fat levels.

Panel A. Associations between the cereal intake and body/blood fat levels.

Variable	beta	95%CI_l	95%CI_h	se	t	r	p	df.e
Body mass index (BMI)	-0.131	-0.137	-0.125	0.003	-43.92	-0.076	0.00E+00	334613
Cholesterol	-0.027	-0.029	-0.026	0.001	-37.66	-0.066	9.99E-310	320045
HDL cholesterol	-0.007	-0.007	-0.006	0.000	-29.61	-0.055	2.15E-192	292978
LDL direct	-0.015	-0.016	-0.014	0.001	-27.10	-0.048	1.57E-161	319441
Triglycerides	-0.010	-0.011	-0.009	0.001	-15.67	-0.028	2.60E-55	319782

Panel B. Associations between the coffee intake and body/blood fat levels.

Variable	beta	95%CI_l	95%CI_h	se	t	r	p	df.e
Body mass index (BMI)	0.129	0.121	0.138	0.004	30.66	0.053	3.84E-206	333987
Cholesterol	0.026	0.024	0.028	0.001	25.15	0.044	1.89E-139	319454
HDL cholesterol	-0.001	-0.002	-0.001	0.000	-3.49	-0.006	4.81E-04	292451
LDL direct	0.022	0.020	0.023	0.001	27.37	0.048	9.02E-165	318853
Triglycerides	0.004	0.003	0.006	0.001	4.83	0.009	1.39E-06	319191

Notes: The table reports the associations Associations between the cereal/coffee intake and body/blood fat levels. The covariates included in the linear regression model are baseline age, gender. Beta refers to the coefficient of the regression model. t, p, se, 95%CI_L and 95%CI_H repsepctively represent the t statistic, two-side p-value, standard error, 95% coffidence interval for coefficients. df.e refers to the degree freedom of the residuals of the regression model. r refers to the correlation coefficient.

Supplementary Table 15. Significant mediation models of SNPs, body/blood fat levels and the intake of cereal and coffee.

Panel A. Description of significant mediation models.

No.	X	Y	Z
1	rs2504706_C	Cereal intake	Body mass index (BMI)
2	rs4410790_C	Cereal intake	Body mass index (BMI)
3	rs2472297_T	Cereal intake	Body mass index (BMI)
4	rs2504706_C	Coffee intake	Body mass index (BMI)
5	rs4410790_C	Coffee intake	Body mass index (BMI)
6	rs2472297_T	Coffee intake	Body mass index (BMI)
7	rs2472297_T	Cereal intake	Cholesterol
8	rs2472297_T	Coffee intake	Cholesterol
9	rs4410790_C	Coffee intake	HDL cholesterol
10	rs2472297_T	Cereal intake	LDL direct
11	rs2472297_T	Coffee intake	LDL direct
12	rs4410790_C	Cereal intake	Triglycerides
13	rs4410790_C	Coffee intake	Triglycerides

Panel B. Statistics of significant mediation models.

X~Z							
No.	Beta	95%CI_l	95%CI_h	se	T	P	R
1	-0.080	-0.107	-0.054	0.014	-5.890	3.86E-09	-0.010
2	0.047	0.023	0.070	0.012	3.896	9.77E-05	0.007
3	0.069	0.044	0.095	0.013	5.317	1.06E-07	0.009
4	-0.081	-0.108	-0.054	0.014	-5.930	3.03E-09	-0.010
5	0.045	0.021	0.068	0.012	3.721	1.98E-04	0.006
6	0.067	0.042	0.093	0.013	5.161	2.46E-07	0.009
7	0.014	0.008	0.020	0.003	4.444	8.85E-06	0.008
8	0.014	0.008	0.020	0.003	4.481	7.44E-06	0.008
9	-0.005	-0.006	-0.003	0.001	-4.851	1.23E-06	-0.009
10	0.011	0.006	0.015	0.002	4.293	1.77E-05	0.008
11	0.010	0.006	0.015	0.002	4.262	2.02E-05	0.008
12	0.015	0.010	0.021	0.003	5.929	3.05E-09	0.011
13	0.015	0.010	0.020	0.003	5.807	6.36E-09	0.010
X~Y							
No.	Beta	95%CI_l	95%CI_h	se	T	P	R

1	0.058	0.042	0.073	0.008	7.304	2.81E-13	0.013
2	-0.038	-0.052	-0.025	0.007	-5.529	3.23E-08	-0.010
3	-0.059	-0.074	-0.045	0.008	-7.871	3.52E-15	-0.014
4	-0.033	-0.044	-0.022	0.006	-5.885	3.99E-09	-0.010
5	0.120	0.110	0.130	0.005	24.338	1.00E-130	0.042
6	0.142	0.132	0.153	0.005	26.577	1.83E-155	0.046
7	-0.059	-0.074	-0.044	0.008	-7.602	2.93E-14	-0.013
8	0.142	0.131	0.152	0.005	25.863	2.50E-147	0.046
9	0.120	0.110	0.130	0.005	22.754	1.67E-114	0.042
10	-0.059	-0.074	-0.044	0.008	-7.610	2.75E-14	-0.013
11	0.142	0.131	0.153	0.005	25.864	2.44E-147	0.046
12	-0.037	-0.051	-0.023	0.007	-5.193	2.07E-07	-0.009
13	0.119	0.109	0.129	0.005	23.596	5.39E-123	0.042

Y~Z/X

No.	Beta	95%CI_l	95%CI_h	se	T	P	R
1	-0.128	-0.134	-0.122	0.003	-42.887	0.00E+00	-0.074
2	-0.128	-0.134	-0.122	0.003	-42.964	0.00E+00	-0.074
3	-0.128	-0.134	-0.122	0.003	-42.934	0.00E+00	-0.074
4	0.132	0.124	0.140	0.004	31.268	2.64E-214	0.054
5	0.132	0.123	0.140	0.004	31.181	3.96E-213	0.054
6	0.131	0.123	0.140	0.004	31.105	4.15E-212	0.054
7	-0.028	-0.029	-0.026	0.001	-37.942	2.63E-314	-0.067
8	0.025	0.023	0.027	0.001	24.610	1.32E-133	0.044
9	-0.001	-0.002	-0.001	0.000	-3.550	3.85E-04	-0.007
10	-0.015	-0.016	-0.014	0.001	-27.281	1.10E-163	-0.048
11	0.021	0.020	0.023	0.001	26.912	2.43E-159	0.048
12	-0.010	-0.011	-0.009	0.001	-15.700	1.60E-55	-0.028
13	0.004	0.002	0.006	0.001	4.583	4.58E-06	0.008

X~Z/Y

No.	Beta	95%CI_l	95%CI_h	se	T	P	R
1	-0.073	-0.100	-0.046	0.014	-5.363	8.18E-08	-0.009
2	0.042	0.018	0.065	0.012	3.496	4.73E-04	0.006
3	0.062	0.036	0.087	0.013	4.746	2.08E-06	0.008
4	-0.077	-0.103	-0.050	0.014	-5.619	1.92E-08	-0.010
5	0.029	0.005	0.053	0.012	2.409	1.60E-02	0.004
6	0.049	0.023	0.074	0.013	3.732	1.90E-04	0.006
7	0.013	0.006	0.019	0.003	3.943	8.05E-05	0.007

8	0.011	0.004	0.017	0.003	3.354	7.97E-04	0.006
9	-0.004	-0.006	-0.003	0.001	-4.697	2.64E-06	-0.009
10	0.010	0.005	0.014	0.002	3.930	8.51E-05	0.007
11	0.007	0.003	0.012	0.002	3.030	2.45E-03	0.005
12	0.015	0.010	0.020	0.003	5.787	7.18E-09	0.010
13	0.015	0.010	0.020	0.003	5.611	2.02E-08	0.010

Z~Y/X							
No.	Beta	95%CI_l	95%CI_h	se	T	P	R
1	-0.043	-0.045	-0.041	0.001	-42.887	0.00E+00	-0.074
2	-0.043	-0.045	-0.041	0.001	-42.964	0.00E+00	-0.074
3	-0.043	-0.045	-0.041	0.001	-42.934	0.00E+00	-0.074
4	0.022	0.021	0.024	0.001	31.268	2.64E-214	0.054
5	0.022	0.021	0.024	0.001	31.181	3.96E-213	0.054
6	0.022	0.021	0.023	0.001	31.105	4.15E-212	0.054
7	-0.163	-0.171	-0.155	0.004	-37.942	2.63E-314	-0.067
8	0.075	0.069	0.081	0.003	24.610	1.32E-133	0.044
9	-0.037	-0.057	-0.016	0.010	-3.550	3.85E-04	-0.007
10	-0.152	-0.163	-0.141	0.006	-27.281	1.10E-163	-0.048
11	0.107	0.099	0.114	0.004	26.912	2.43E-159	0.048
12	-0.076	-0.086	-0.067	0.005	-15.700	1.60E-55	-0.028
13	0.016	0.009	0.023	0.003	4.583	4.58E-06	0.008

X~Y/Z							
No.	Beta	95%CI_l	95%CI_h	se	T	P	R
1	0.054	0.039	0.070	0.008	6.886	5.76E-12	0.012
2	-0.036	-0.050	-0.023	0.007	-5.254	1.49E-07	-0.009
3	-0.056	-0.071	-0.042	0.008	-7.498	6.51E-14	-0.013
4	-0.031	-0.042	-0.020	0.006	-5.572	2.52E-08	-0.010
5	0.119	0.109	0.129	0.005	24.172	5.64E-129	0.042
6	0.141	0.130	0.151	0.005	26.336	1.06E-152	0.046
7	-0.056	-0.072	-0.041	0.008	-7.320	2.48E-13	-0.013
8	0.141	0.130	0.151	0.005	25.691	2.08E-145	0.045
9	0.120	0.110	0.130	0.005	22.721	3.48E-114	0.042
10	-0.057	-0.072	-0.042	0.008	-7.411	1.26E-13	-0.013
11	0.141	0.130	0.151	0.005	25.689	2.19E-145	0.045
12	-0.036	-0.050	-0.022	0.007	-5.029	4.92E-07	-0.009
13	0.119	0.109	0.129	0.005	23.548	1.66E-122	0.042

Panel C. Model selection.

No.	X~Y~Z Bootsrap P value	Theoretic P value	Proportion of Mediation (PM)	X~Z~Y Bootsrap P value	Theoretic P value	Proportion of Mediation (PM)	Model comparation Excess PM of X~Y~Z than X~Z~Y	P value of Excess PM
1	0	1.68E-12	9.19%	0	4.64E-09	5.97%	3.21%	0.3
2	0	8.95E-08	10.53%	0	8.61E-05	5.23%	5.30%	0.234
3	0	1.60E-14	10.97%	0	2.39E-07	5.00%	5.97%	0.07
4	0	2.66E-09	5.37%	0	6.45E-09	5.45%	-0.08%	0.996
5	0	1.41E-81	35.31%	0	1.74E-04	0.83%	34.48%	0
6	0	6.72E-83	27.71%	0	6.47E-07	1.04%	26.66%	0
7	0	6.51E-14	11.46%	0	1.17E-05	3.92%	7.54%	0.024
8	0	6.82E-68	25.14%	0	1.43E-05	0.75%	24.38%	0
9	0	2.28E-04	3.09%	0	4.61E-03	0.14%	2.95%	0
10	0	1.80E-13	8.56%	0	3.04E-05	2.72%	5.83%	0.012
11	0	4.65E-75	28.92%	0	4.11E-05	0.79%	28.13%	0
12	0	1.33E-06	2.44%	0	3.79E-08	3.18%	-0.74%	0.588
13	0	1.04E-05	3.30%	0	4.52E-04	0.20%	3.10%	0

Notes: The table reports significant mediation models of SNPs, body/blood fat levels and the intake of cereal and coffee. Panel A displays significant mediation models which were tested using Baron and Kenny's (1986) [1] causal steps approach and corrected for mutiple testing using Bonferroni procedure. In the analysis, all possible mediation models between SNPs, body/blood fat levels and cereal/coffee intake were assessed. The covariates included were baseline age, gender and the top 40 genetic components obtained from UK Biobank. SNPs refers to the shared lead variants of the cereal and coffee intake. Panel B shows the statistics of the significant mediation models. A~B means that A is the independent variable and B is the dependent variable in the regression model. A~B/C means that A is the independent variable and B is the dependent variable in the regression model while C is added to the model as covariates. For example, in the first mediation model, X is the C-allele of rs2504706, Y is the cereal intake, and Z is the body mass index(BMI). Here, in the X~Z model, we tested the association between the C-allele of rs2504706 and BMI. Besides, in the X~Z/Y model, we tested the association between the C-allele of rs2504706 and BMI with the cereal intake adjusted. Beta refers to the coefficient of the regression model. T, P, se, 95%CI_L and 95%CI_H repsepctively represent the t statistic, two-side p-value, standard error, 95% coffidence interval for coefficients. R refers to the correlation coefficient. Panel C shows the comparation between possible directions of the mediation models. X~Y~Z means that X is the independent variable, Y is the mediation variable and Z is the dependent variable. A complete mediation model X~Y~Z consists of four parts: X~Z, X~Y, Y~Z/X and X~Z/Y. The bootstrap P value of the mediation model was obtained from 1000 boostrap procedure. Theoretic P value was computed using normal distribution. Proportion of Mediation (PM) was calculated by the regression coeffecients of the X~Z and X~Z/Y model. Excess PM of X~Y~Z than X~Z~Y is the difference bwteen the PM of X~Y~Z and the X~Z~Y. The P value of Excess PM is obtained from the 1000 bootstrap procedure.

References

[1] R. M. Baron, D. A. Kenny, The moderator–mediator variable distinction in social psychological research: Conceptual, strategic, and statistical considerations. Journal of personality and social psychology 51, 1173 (1986).

Supplementary Table 16. Associations between the GMV-association patterns of cognitive functions and cereal/coffee intake.

Panel A. Associations between the GMV-association patterns of cognitive functions and cereal/coffee intake (follow-up).

Variable	Cereal intake		Coffee intake	
	R	P_perm	R	P_perm
Fluid intelligence	0.6292	2.00E-04	-0.4230	1.52E-02
Numeric memory	0.6566	0.00E+00	-0.4553	8.20E-03
Prospective memory	0.4812	4.80E-03	-0.4259	1.36E-02
Reaction time	-0.4430	9.80E-03	0.3069	8.47E-02
Pairs matching	-0.4396	8.40E-03	0.5763	2.00E-04
Matrix pattern completion	0.7104	0.00E+00	-0.6213	1.00E-04
Symbol digit substitution	0.6875	0.00E+00	-0.6187	0.00E+00
Tower rearranging	0.4178	1.66E-02	-0.3914	2.71E-02
Numeric trail making	0.6251	0.00E+00	-0.6043	0.00E+00
Alphanumeric trail making	0.6873	0.00E+00	-0.7018	0.00E+00

Panel B. Associations between the GMV-association patterns of cognitive functions and cereal/coffee intake (baseline).

Variable	Cereal intake		Coffee intake	
	R	P_perm	R	P_perm
Fluid intelligence	0.6177	0.00E+00	-0.4353	1.24E-02
Numeric memory	0.0717	6.91E-01	0.1005	5.79E-01
Prospective memory	0.4122	2.00E-02	-0.3661	3.50E-02
Reaction time	-0.3931	2.06E-02	0.2239	2.14E-01
Pairs matching	-0.4448	9.30E-03	0.3957	2.13E-02
Matrix pattern completion	0.7693	0.00E+00	-0.6601	0.00E+00
Symbol digit substitution	0.7832	0.00E+00	-0.6864	0.00E+00
Tower rearranging	0.4482	8.10E-03	-0.4022	2.21E-02
Numeric trail making	0.6506	0.00E+00	-0.5945	2.00E-04
Alphanumeric trail making	0.7320	0.00E+00	-0.6902	1.00E-04

Notes: The table reports the associations between the GMV-association patterns of cognitive functions and cereal/coffee intake in the discovery sample. Panel A and B show associations between the GMV-association patterns of the follow-up and baseline phenotypes respectively. R refers to the spatial correlation coefficient and P_perm represents the P value of the correlation coefficients based on 10000 permutation procedure.

Supplementary Table 17. Validation of the associations between the GMV-association patterns of cognitive functions and cereal/coffee intake.

Panel A. Associations between the GMV-association patterns of cognitive functions and cereal/coffee intake (follow-up).

	Cereal intake		Coffee intake	
Variable	R	P_one-tailed	R	P_one-tailed
Fluid intelligence	0.724	0.0E+00	NA	
Matrix pattern completion	0.782	0.0E+00	-0.403	1.0E-02
Symbol digit substitution	0.769	0.0E+00	-0.561	3.0E-04
Numeric trail making	0.578	4.5E-04	-0.536	1.2E-03
Alphanumeric trail making	0.770	0.0E+00	-0.579	2.5E-04

Panel B. Associations between the GMV-association patterns of cognitive functions and cereal/coffee intake (baseline).

	Cereal intake		Coffee intake	
Variable	R	P_one-tailed	R	P_one-tailed
Fluid intelligence	0.538	6.0E-04	NA	
Matrix pattern completion	0.776	0.0E+00	-0.417	7.7E-03
Symbol digit substitution	0.853	0.0E+00	-0.554	7.5E-04
Numeric trail making	0.687	0.0E+00	-0.547	4.5E-04
Alphanumeric trail making	0.741	0.0E+00	-0.576	2.0E-04

Notes: The table reports the associations between the GMV-association patterns of cognitive functions and cereal/coffee intake in the replication sample. Panel A and B show associations between the GMV-association patterns of the follow-up and baseline phenotypes respectively. R refers to the spatial correlation coefficient and P_one-tailed represents the one-tailed P value of the correlation coefficients based on 10000 permutation procedure.

Supplementary Table 18: Associations between the GMV-association patterns of the cereal/coffee intake and the spatial expression of genes.

Panel A. Genes mapped by the shared lead variants based on eQTL mapping

ensg	symbol	chr	start	end	IndSigSNPs	No.
ENSG00000170915	PAQR8	6	52226219	52272575	rs2504706	1
ENSG00000106546	AHR	7	17338246	17385776	rs4410790	2
ENSG00000048052	HDAC9	7	18126572	19042039	rs4410790	3
ENSG00000071189	SNX13	7	17830385	17980124	rs4410790	4
ENSG00000171243	SOSTDC1	7	16501106	16570205	rs4410790	5
ENSG00000106537	TSPAN13	7	16793160	16824161	rs4410790	6
ENSG00000103653	CSK	15	75074398	75095539	rs2472297	7
ENSG00000129009	ISLR	15	74466012	74469213	rs2472297	8
ENSG00000129038	LOXL1	15	74218330	74244478	rs2472297	9
ENSG00000140400	MAN2C1	15	75648133	75660971	rs2472297	10
ENSG00000178802	MPI	15	75182346	75191798	rs2472297	11
ENSG00000140464	PML	15	74287014	74340153	rs2472297	12
ENSG00000169410	PTPN9	15	75759462	75871630	rs2472297	13
ENSG00000138623	SEMA7A	15	74701630	74726808	rs2472297	14
ENSG00000067221	STOML1	15	74275547	74286963	rs2472297	15
ENSG00000140497	SCAMP2	15	75136071	75165706	rs2472297	16
ENSG00000169371	SNUPN	15	75890424	75918810	rs2472297	17
ENSG00000169375	SIN3A	15	75661720	75748183	rs2472297	18
ENSG00000140365	COMMD4	15	75628232	75634268	rs2472297	19
ENSG00000177971	IMP3	15	75931426	75941047	rs2472297	20
ENSG00000167173	C15orf39	15	75487984	75504510	rs2472297	21
ENSG00000178761	FAM219B	15	75192328	75199462	rs2472297	22
ENSG00000167178	ISLR2	15	74392652	74430881	rs2472297	23
ENSG00000138621	PPCDC	15	75315896	75409803	rs2472297	24
ENSG00000140398	NEIL1	15	75639296	75647592	rs2472297	25
ENSG00000140506	LMAN1L	15	75105057	75118099	rs2472297	26
ENSG00000179151	EDC3	15	74922899	74988633	rs2472297	27
ENSG00000138629	UBL7	15	74738318	74753523	rs2472297	28
ENSG00000182950	ODF3L1	15	76016318	76020029	rs2472297	29
ENSG00000198794	SCAMP5	15	75249560	75313837	rs2472297	30
ENSG00000213578	CPLX3	15	75118888	75124141	rs2472297	31

Panel B. Association between the GMV-association patterns of cereal/coffee intake and spatial expression patterns of mapped genes.

	Cereal intake			Coffee intake		
symbol	R	P_perm	FDR	R	P_perm	FDR
PAQR8	-0.1236	0.5728	0.7731	0.0121	0.9553	0.9818
AHR	0.2110	0.4152	0.7107	-0.5764	0.0030	0.0333
HDAC9	0.3952	0.0703	0.2724	-0.5828	0.0016	0.0333
SNX13	-0.0009	0.9953	0.9953	-0.1543	0.2854	0.6452
SOSTDC1	0.1868	0.4992	0.7255	-0.4163	0.0946	0.3087
TSPAN13	0.2506	0.3784	0.7107	-0.6340	0.0043	0.0333
CSK	-0.1701	0.4239	0.7107	0.5100	0.0020	0.0333

ISLR	0.1493	0.5736	0.7731	-0.4290	0.0655	0.2707
LOXL1	0.1000	0.6380	0.8070	-0.2116	0.2914	0.6452
MAN2C1	-0.0210	0.9234	0.9818	0.1608	0.4241	0.7107
MPI	0.2229	0.2847	0.6452	-0.4723	0.0035	0.0333
PML	0.2057	0.2529	0.6272	-0.4198	0.0043	0.0333
PTPN9	0.1916	0.4476	0.7116	-0.3645	0.1209	0.3569
SEMA7A	-0.1685	0.3427	0.7107	-0.0681	0.7029	0.8070
STOML1	0.3875	0.1639	0.4619	-0.6437	0.0033	0.0333
SCAMP2	-0.0420	0.7987	0.8843	-0.1404	0.3915	0.7107
SNUPN	0.3137	0.2141	0.5531	-0.4634	0.0420	0.1864
SIN3A	-0.1270	0.5032	0.7255	0.4013	0.0164	0.0924
COMMD4	0.1329	0.3881	0.7107	-0.3447	0.0074	0.0459
IMP3	0.1356	0.5161	0.7272	-0.2717	0.1733	0.4672
C15orf39	0.0865	0.6984	0.8070	-0.1532	0.4910	0.7255
FAM219B	-0.0090	0.9660	0.9818	-0.0842	0.6825	0.8070
ISLR2	0.2473	0.3861	0.7107	-0.4618	0.0782	0.2852
PPCDC	-0.1075	0.5919	0.7808	0.0802	0.6957	0.8070
NEIL1	-0.1162	0.6314	0.8070	0.1878	0.4364	0.7116
LMAN1L	-0.0849	0.6943	0.8070	0.3244	0.0937	0.3087
EDC3	0.0631	0.7842	0.8840	0.0137	0.9533	0.9818
UBL7	0.1920	0.4862	0.7255	-0.5072	0.0294	0.1519
ODF3L1	-0.0162	0.9413	0.9818	0.3239	0.1104	0.3422
SCAMP5	0.2328	0.3772	0.7107	-0.4699	0.0421	0.1864
CPLX3	0.4721	0.0029	0.0333	-0.4429	0.0072	0.0459

Notes: The table reports the associations between the GMV-association patterns of the cereal/coffee intake and the spatial expression of genes. The analyses were conducted using the follow-up phenotypes in the discovary sample. Panel A shows the genes mapped by shared lead variants of the cereal and coffee intake based on eQTL mapping using FUMA [1] software. Panel B show associations between the GMV-association patterns of the follow-up phenotypes respectively. R refers to the spatial correlation coefficient and P_perm represents the P value of the correlation coefficients based on 10000 permutation procedure. FDR was calculated based on the P_perm using Benjamini-Hochberg method.

References

[1] K. Watanabe, E. Taskesen, A. Van Bochoven, D. Posthuma, Functional mapping and annotation of genetic associations with FUMA. *Nature communications* **8**, 1826 (2017).

Supplementary Table 19: Association between the GMV-association patterns of the cognitive functions and the expression pattern of the CPLX3 gene.

Variable	R	P_perm	FDR
Fluid intelligence	0.4227	0.0098	0.0163
Numeric memory	0.4907	0.0013	0.0065
Prospective memory	0.4429	0.0074	0.0148
Reaction time	-0.2280	0.2351	0.2351
Pairs matching	-0.3275	0.0704	0.0782
Matrix pattern completion	0.4564	0.0042	0.0140
Symbol digit substitution	0.3948	0.0192	0.0274
Tower rearranging	0.3385	0.0613	0.0766
Numeric trail making	0.4416	0.0064	0.0148
Alphanumeric trail making	0.5456	0	0.0000

Notes: The table reports theAssociations between the GMV-association patterns of cognitive functions and the spatial expression of the CPLX3 gene. The analyses were conducted using the follow-up phenotypes in the discovary sample. R refers to the spatial correlation coefficient and P_perm represents the P value of the correlation coefficients based on 10000 permutation procedure. FDR was calculated based on the P_perm using Benjamini-Hochberg method.

Supplementary Table 20. Validation of associations between the gene-expression pattern of CPLX3 gene and the GMV-association patterns of phenotypes.

Panel A. Associations between the gene-expression pattern of CPLX3 gene and the GMV-association patterns of cereal/coffee intake.

Phenotypes	R	P_one-tailed
Cereal intake	0.396	1.1E-02
Coffee intake	-0.360	2.0E-02

Panel B. Association between the GMV-association patterns of the cognitive functions and the gene-expression patterns of CPLX3 gene.

Phenotypes	R	P_one-tailed
Fluid intelligence	0.476	1.4E-03
Numeric memory	0.466	1.8E-03
Prospective memory	0.388	1.1E-02
Matrix pattern completion	0.474	1.1E-03
Symbol digit substitution	0.468	1.8E-03
Numeric trail making	0.463	2.1E-03
Alphanumeric trail making	0.500	7.0E-04

Notes: The table reports theAssociations between the GMV-association patterns of phenotypes and the spatial expression of the CPLX3 gene. The analyses were conducted using the follow-up phenotypes in the replication sample. R refers to the spatial correlation coefficient and P_one-tailed represents the one-tailed P value of the correlation coefficients based on 10000 permutation procedure.

Supplementary Table 21: The GMV-association patterns of the follow-up cereal/coffee intake and cognitive functions in the discovery sample.

No.	AAL3 region	Region name	Lobe	Cereal intake	Coffee intake	Matrix pattern completion	Symbol digit substitution	Numeric trail making	Alphanumeric trail making
1	Precentral_L	PreCG.L	Sensorimotor	0.035(1.8E-06)	-0.044(2.2E-09)	0.026(2.6E-02)	0.026(2.5E-02)	-0.036(1.4E-03)	-0.028(1.4E-02)
2	Precentral_R	PreCG.R	Sensorimotor	0.051(2.7E-12)	-0.044(2.3E-09)	0.033(4.1E-03)	0.027(2.0E-02)	-0.030(8.3E-03)	-0.019(1.0E-01)
3	Frontal_Sup_2_L	SFGdor2.L	Frontal	0.056(1.6E-14)	-0.048(4.7E-11)	0.042(2.3E-04)	0.032(5.1E-03)	-0.028(1.4E-02)	-0.044(1.3E-04)
4	Frontal_Sup_2_R	SFGdor2.R	Frontal	0.059(7.6E-16)	-0.055(6.4E-14)	0.045(9.4E-05)	0.041(3.3E-04)	-0.023(4.8E-02)	-0.037(1.2E-03)
5	Frontal_Mid_2_L	MFG2.L	Frontal	0.053(5.2E-13)	-0.046(3.0E-10)	0.035(2.5E-03)	0.037(1.5E-03)	-0.038(8.6E-04)	-0.037(1.1E-03)
6	Frontal_Mid_2_R	MFG2.R	Frontal	0.062(1.2E-17)	-0.054(1.5E-13)	0.033(4.6E-03)	0.026(2.6E-02)	-0.029(1.3E-02)	-0.044(1.4E-04)
7	Frontal_Inf_Oper_L	IFGoperc.L	Frontal	0.043(3.8E-09)	-0.042(1.0E-08)	0.024(3.9E-02)	0.032(5.6E-03)	-0.035(2.1E-03)	-0.035(2.2E-03)
8	Frontal_Inf_Oper_R	IFGoperc.R	Frontal	0.051(4.3E-12)	-0.041(2.6E-08)	0.035(2.4E-03)	0.031(7.0E-03)	-0.036(1.9E-03)	-0.031(7.3E-03)
9	Frontal_Inf_Tri_L	IFGtriang.L	Frontal	0.053(2.7E-13)	-0.044(2.4E-09)	0.037(1.4E-03)	0.051(9.8E-06)	-0.049(1.7E-05)	-0.055(1.2E-06)
10	Frontal_Inf_Tri_R	IFGtriang.R	Frontal	0.054(1.5E-13)	-0.049(3.0E-11)	0.045(8.7E-05)	0.036(1.5E-03)	-0.037(1.1E-03)	-0.055(1.6E-06)
11	Frontal_Inf_Orb_2_L	ORBinf.L	Frontal	0.063(3.7E-18)	-0.035(2.3E-06)	0.060(1.7E-07)	0.050(1.5E-05)	-0.034(2.6E-03)	-0.062(5.3E-08)
12	Frontal_Inf_Orb_2_R	ORBinf.R	Frontal	0.064(2.3E-18)	-0.037(4.5E-07)	0.073(1.7E-10)	0.061(1.0E-07)	-0.044(1.1E-04)	-0.068(2.6E-09)
13	Rolandic_Oper_L	ROL.L	Frontal	0.042(6.7E-09)	-0.037(3.2E-07)	0.040(5.9E-04)	0.033(4.0E-03)	-0.022(5.4E-02)	-0.045(9.0E-05)
14	Rolandic_Oper_R	ROL.R	Frontal	0.053(2.5E-13)	-0.041(3.0E-08)	0.025(2.8E-02)	0.029(1.3E-02)	-0.007(5.6E-01)	-0.034(2.8E-03)
15	Supp_Motor_Area_L	SMA.L	Sensorimotor	0.038(2.4E-07)	-0.040(3.3E-08)	0.011(3.3E-01)	0.020(8.6E-02)	-0.015(2.0E-01)	-0.014(2.2E-01)
16	Supp_Motor_Area_R	SMA.R	Sensorimotor	0.039(1.1E-07)	-0.040(4.1E-08)	0.010(3.7E-01)	0.025(3.0E-02)	-0.025(2.9E-02)	-0.020(7.6E-02)
17	Olfactory_L	OLF.L	Frontal	0.046(2.6E-10)	-0.026(3.7E-04)	0.029(1.2E-02)	0.032(5.1E-03)	-0.011(3.3E-01)	-0.020(8.3E-02)
18	Olfactory_R	OLF.R	Frontal	0.039(1.0E-07)	-0.027(1.9E-04)	0.021(7.4E-02)	0.014(2.4E-01)	0.000(9.8E-01)	-0.018(1.1E-01)
19	Frontal_Sup_Medial_L	SFGmed.L	Frontal	0.070(1.0E-21)	-0.045(5.1E-10)	0.024(3.8E-02)	0.032(4.9E-03)	-0.022(5.6E-02)	-0.035(2.5E-03)
20	Frontal_Sup_Medial_R	SFGmed.R	Frontal	0.061(7.5E-17)	-0.047(8.7E-11)	0.035(2.4E-03)	0.033(3.7E-03)	-0.008(4.6E-01)	-0.025(2.7E-02)
21	Frontal_Med_Orb_L	ORBsupmed.L	Frontal	0.067(6.3E-20)	-0.044(2.6E-09)	0.046(5.6E-05)	0.046(6.7E-05)	-0.037(1.2E-03)	-0.041(3.6E-04)
22	Frontal_Med_Orb_R	ORBsupmed.R	Frontal	0.065(4.0E-19)	-0.045(5.6E-10)	0.046(7.2E-05)	0.043(1.9E-04)	-0.033(3.8E-03)	-0.049(1.9E-05)
23	Rectus_L	REC.L	Frontal	0.066(1.9E-19)	-0.035(2.2E-06)	0.062(7.8E-08)	0.053(3.2E-06)	-0.027(1.7E-02)	-0.036(1.5E-03)
24	Rectus_R	REC.R	Frontal	0.060(1.5E-16)	-0.036(9.2E-07)	0.064(2.6E-08)	0.041(3.8E-04)	-0.020(8.3E-02)	-0.037(1.1E-03)
25	OFCmed_L	OFCmed.L	Frontal	0.052(1.3E-12)	-0.038(1.9E-07)	0.045(7.7E-05)	0.039(7.5E-04)	-0.024(3.8E-02)	-0.036(1.7E-03)
26	OFCmed_R	OFCmed.R	Frontal	0.041(1.5E-08)	-0.037(3.3E-07)	0.032(4.8E-03)	0.013(2.6E-01)	-0.018(1.2E-01)	-0.032(5.1E-03)
27	OFCant_L	OFCant.L	Frontal	0.036(7.9E-07)	-0.037(3.6E-07)	0.027(1.9E-02)	0.028(1.3E-02)	-0.028(1.5E-02)	-0.041(3.8E-04)
28	OFCant_R	OFCant.R	Frontal	0.048(5.0E-11)	-0.033(5.2E-06)	0.037(1.4E-03)	0.037(1.4E-03)	-0.034(2.7E-03)	-0.051(7.9E-06)

29	OFCpost_L	OFCpost.L	Frontal	0.037(5.3E-07)	-0.036(6.9E-07)	0.029(1.1E-02)	0.033(4.4E-03)	-0.024(3.5E-02)	-0.044(1.4E-04)
30	OFCpost_R	OFCpost.R	Frontal	0.053(4.9E-13)	-0.033(5.2E-06)	0.025(3.0E-02)	0.029(1.1E-02)	-0.026(2.4E-02)	-0.035(2.3E-03)
31	OFClat_L	OFClat.L	Frontal	0.049(1.6E-11)	-0.032(1.0E-05)	0.047(4.0E-05)	0.023(4.2E-02)	-0.024(3.8E-02)	-0.042(2.3E-04)
32	OFClat_R	OFClat.R	Frontal	0.061(4.1E-17)	-0.032(1.4E-05)	0.063(3.6E-08)	0.035(2.1E-03)	-0.035(2.4E-03)	-0.048(2.6E-05)
33	Insula_L	INS.L	Subcortical	0.052(9.1E-13)	-0.054(2.3E-13)	0.038(8.8E-04)	0.048(2.6E-05)	-0.025(2.7E-02)	-0.052(4.5E-06)
34	Insula_R	INS.R	Subcortical	0.063(4.8E-18)	-0.054(1.3E-13)	0.055(1.8E-06)	0.052(6.7E-06)	-0.024(3.7E-02)	-0.058(3.2E-07)
35	Cingulate_Mid_L	DCG.L	Frontal	0.072(7.1E-23)	-0.050(6.3E-12)	0.037(1.3E-03)	0.041(3.6E-04)	-0.028(1.6E-02)	-0.047(3.6E-05)
36	Cingulate_Mid_R	DCG.R	Frontal	0.075(1.3E-24)	-0.048(4.0E-11)	0.032(5.3E-03)	0.053(3.4E-06)	-0.036(1.5E-03)	-0.049(1.8E-05)
37	Cingulate_Post_L	PCG.L	Parietal	0.026(3.4E-04)	-0.023(2.0E-03)	0.008(5.1E-01)	0.013(2.6E-01)	-0.001(9.4E-01)	-0.012(3.0E-01)
38	Cingulate_Post_R	PCG.R	Parietal	0.017(2.3E-02)	-0.010(1.9E-01)	-0.007(5.3E-01)	-0.004(7.2E-01)	0.013(2.6E-01)	0.001(9.6E-01)
39	Hippocampus_L	HIPPOL	Temporal	0.040(4.0E-08)	-0.008(2.5E-01)	0.016(1.5E-01)	0.042(2.6E-04)	-0.015(1.8E-01)	-0.013(2.6E-01)
40	Hippocampus_R	HIPPOR	Temporal	0.033(5.9E-06)	-0.010(1.8E-01)	0.008(4.9E-01)	0.012(3.1E-01)	0.008(4.7E-01)	0.013(2.7E-01)
41	ParaHippocampal_L	PARA_HIPPOL	Temporal	0.049(2.6E-11)	-0.015(4.1E-02)	0.033(3.9E-03)	0.050(1.1E-05)	-0.024(3.6E-02)	-0.031(6.7E-03)
42	ParaHippocampal_R	PARA_HIPPOR	Temporal	0.054(1.4E-13)	-0.017(1.7E-02)	0.025(2.9E-02)	0.044(1.2E-04)	-0.018(1.1E-01)	-0.031(6.6E-03)
43	Amygdala_L	AMYGDL	Subcortical	0.039(9.7E-08)	-0.033(7.2E-06)	0.046(6.4E-05)	0.071(6.2E-10)	-0.036(1.9E-03)	-0.045(8.6E-05)
44	Amygdala_R	AMYGDR	Subcortical	0.047(1.3E-10)	-0.042(1.4E-08)	0.058(5.4E-07)	0.076(3.7E-11)	-0.033(4.5E-03)	-0.048(2.7E-05)
45	Calcarine_L	CAL.L	Occipital	0.006(3.9E-01)	-0.037(3.7E-07)	-0.002(8.6E-01)	0.014(2.3E-01)	-0.023(4.3E-02)	-0.011(3.5E-01)
46	Calcarine_R	CAL.R	Occipital	0.008(2.6E-01)	-0.036(1.0E-06)	0.003(7.8E-01)	0.012(3.0E-01)	-0.012(3.0E-01)	-0.012(2.8E-01)
47	Cuneus_L	CUN.L	Occipital	0.015(3.9E-02)	-0.032(1.4E-05)	-0.002(8.9E-01)	0.014(2.2E-01)	-0.023(4.1E-02)	-0.019(9.0E-02)
48	Cuneus_R	CUN.R	Occipital	0.025(5.3E-04)	-0.037(5.4E-07)	-0.001(9.4E-01)	0.021(7.4E-02)	-0.021(7.0E-02)	-0.018(1.1E-01)
49	Lingual_L	LING.L	Occipital	0.019(8.0E-03)	-0.034(2.5E-06)	-0.012(2.8E-01)	0.011(3.3E-01)	-0.015(1.9E-01)	-0.015(1.9E-01)
50	Lingual_R	LING.R	Occipital	0.024(1.2E-03)	-0.027(2.1E-04)	0.005(6.7E-01)	0.003(7.7E-01)	-0.018(1.1E-01)	-0.015(1.8E-01)
51	Occipital_Sup_L	SOG.L	Occipital	0.030(4.1E-05)	-0.033(6.0E-06)	0.003(7.7E-01)	0.019(1.1E-01)	-0.012(2.9E-01)	-0.024(3.4E-02)
52	Occipital_Sup_R	SOG.R	Occipital	0.040(4.5E-08)	-0.034(4.6E-06)	-0.005(6.4E-01)	0.015(1.9E-01)	-0.032(4.6E-03)	-0.015(1.9E-01)
53	Occipital_Mid_L	MOG.L	Occipital	0.017(2.2E-02)	-0.033(7.2E-06)	0.007(5.2E-01)	0.000(9.9E-01)	-0.006(6.3E-01)	-0.010(3.8E-01)
54	Occipital_Mid_R	MOG.R	Occipital	0.026(4.7E-04)	-0.032(1.6E-05)	0.012(3.0E-01)	-0.003(7.9E-01)	-0.019(9.0E-02)	-0.022(5.5E-02)
55	Occipital_Inf_L	IOG.L	Occipital	0.006(3.8E-01)	-0.037(5.6E-07)	0.002(8.8E-01)	-0.015(2.1E-01)	0.000(1.0E+00)	-0.028(1.4E-02)
56	Occipital_Inf_R	IOG.R	Occipital	0.006(4.3E-01)	-0.023(2.0E-03)	-0.005(7.0E-01)	-0.023(4.8E-02)	-0.016(1.5E-01)	-0.015(1.8E-01)
57	Fusiform_L	FFG.L	Temporal	0.039(1.0E-07)	-0.019(8.6E-03)	0.051(7.7E-06)	0.049(2.0E-05)	-0.036(1.9E-03)	-0.044(1.1E-04)
58	Fusiform_R	FFG.R	Temporal	0.058(1.2E-15)	-0.023(1.9E-03)	0.056(1.3E-06)	0.039(6.6E-04)	-0.039(5.8E-04)	-0.044(1.2E-04)
59	Postcentral_L	PoCG.L	Sensorimotor	0.059(6.6E-16)	-0.040(4.1E-08)	0.035(2.5E-03)	0.041(3.9E-04)	-0.028(1.3E-02)	-0.034(2.7E-03)
60	Postcentral_R	PoCG.R	Sensorimotor	0.058(2.1E-15)	-0.029(9.8E-05)	0.023(5.0E-02)	0.022(5.6E-02)	-0.010(4.0E-01)	-0.029(1.3E-02)

61	Parietal_Sup_L	SPG.L	Parietal	0.026(4.1E-04)	-0.024(1.2E-03)	-0.005(6.4E-01)	0.010(4.0E-01)	-0.024(4.0E-02)	-0.018(1.2E-01)
62	Parietal_Sup_R	SPG.R	Parietal	0.020(7.4E-03)	-0.031(2.1E-05)	-0.022(6.0E-02)	-0.004(7.4E-01)	-0.013(2.6E-01)	-0.008(4.6E-01)
63	Parietal_Inf_L	IPL.L	Parietal	0.054(1.8E-13)	-0.047(1.2E-10)	0.009(4.2E-01)	0.037(1.5E-03)	-0.032(4.8E-03)	-0.026(2.3E-02)
64	Parietal_Inf_R	IPL.R	Parietal	0.044(2.2E-09)	-0.031(1.7E-05)	-0.003(7.7E-01)	0.027(2.1E-02)	-0.010(4.0E-01)	-0.023(4.1E-02)
65	SupraMarginal_L	SMG.L	Parietal	0.052(1.3E-12)	-0.036(8.7E-07)	0.022(5.9E-02)	0.055(1.6E-06)	-0.039(6.9E-04)	-0.038(8.9E-04)
66	SupraMarginal_R	SMG.R	Parietal	0.060(2.4E-16)	-0.028(1.3E-04)	0.028(1.7E-02)	0.049(1.8E-05)	-0.020(8.8E-02)	-0.030(9.5E-03)
67	Angular_L	ANG.L	Parietal	0.030(4.1E-05)	-0.039(8.4E-08)	0.017(1.4E-01)	0.020(8.1E-02)	-0.012(2.9E-01)	-0.013(2.5E-01)
68	Angular_R	ANG.R	Parietal	0.034(2.5E-06)	-0.035(2.4E-06)	0.017(1.4E-01)	0.026(2.4E-02)	-0.009(4.3E-01)	-0.017(1.3E-01)
69	Precuneus_L	PCUN.L	Parietal	0.032(1.4E-05)	-0.027(1.9E-04)	0.014(2.4E-01)	0.007(5.6E-01)	-0.011(3.4E-01)	-0.021(6.2E-02)
70	Precuneus_R	PCUN.R	Parietal	0.036(8.1E-07)	-0.031(2.4E-05)	0.008(4.9E-01)	0.016(1.6E-01)	-0.017(1.3E-01)	-0.023(4.7E-02)
71	Paracentral_Lobule_L	PCL.L	Parietal	0.020(7.4E-03)	-0.028(1.4E-04)	0.003(8.2E-01)	0.016(1.7E-01)	-0.025(2.8E-02)	-0.010(3.7E-01)
72	Paracentral_Lobule_R	PCL.R	Parietal	0.030(3.3E-05)	-0.020(5.5E-03)	0.003(8.0E-01)	0.023(4.5E-02)	-0.016(1.5E-01)	-0.026(2.3E-02)
73	Caudate_L	CAU.L	Subcortical	0.001(9.3E-01)	-0.010(1.5E-01)	-0.020(7.6E-02)	-0.030(8.7E-03)	0.024(3.7E-02)	0.032(4.9E-03)
74	Caudate_R	CAU.R	Subcortical	0.004(5.8E-01)	-0.019(8.3E-03)	-0.026(2.6E-02)	-0.032(5.4E-03)	0.024(3.5E-02)	0.024(3.9E-02)
75	Putamen_L	PUT.L	Subcortical	0.043(2.7E-09)	-0.033(6.7E-06)	0.051(8.0E-06)	0.084(2.0E-13)	-0.031(7.6E-03)	-0.050(1.1E-05)
76	Putamen_R	PUT.R	Subcortical	0.046(3.5E-10)	-0.039(8.8E-08)	0.051(9.5E-06)	0.086(8.2E-14)	-0.032(5.6E-03)	-0.055(1.5E-06)
77	Pallidum_L	PAL.L	Subcortical	0.018(1.5E-02)	-0.013(7.1E-02)	0.030(9.1E-03)	0.046(6.3E-05)	-0.012(3.1E-01)	-0.017(1.5E-01)
78	Pallidum_R	PAL.R	Subcortical	0.021(5.0E-03)	-0.019(1.1E-02)	0.035(2.3E-03)	0.058(4.7E-07)	-0.021(6.5E-02)	-0.025(3.0E-02)
79	Heschl_L	HES.L	Temporal	0.042(1.3E-08)	-0.049(1.5E-11)	0.036(1.6E-03)	0.035(2.5E-03)	-0.025(3.1E-02)	-0.050(1.5E-05)
80	Heschl_R	HES.R	Temporal	0.043(2.8E-09)	-0.040(4.7E-08)	0.046(5.8E-05)	0.047(3.9E-05)	-0.027(2.0E-02)	-0.043(1.9E-04)
81	Temporal_Sup_L	STG.L	Temporal	0.056(2.2E-14)	-0.052(1.3E-12)	0.051(8.0E-06)	0.055(1.6E-06)	-0.034(3.1E-03)	-0.056(1.0E-06)
82	Temporal_Sup_R	STG.R	Temporal	0.053(2.4E-13)	-0.046(3.0E-10)	0.067(5.1E-09)	0.050(1.2E-05)	-0.014(2.1E-01)	-0.048(2.8E-05)
83	Temporal_Pole_Sup_L	TPOsup.L	Temporal	0.048(6.8E-11)	-0.037(3.6E-07)	0.029(1.2E-02)	0.047(4.1E-05)	-0.026(2.3E-02)	-0.045(7.8E-05)
84	Temporal_Pole_Sup_R	TPOsup.R	Temporal	0.054(1.7E-13)	-0.045(8.9E-10)	0.034(2.8E-03)	0.030(9.3E-03)	-0.009(4.6E-01)	-0.037(1.2E-03)
85	Temporal_Mid_L	MTG.L	Temporal	0.049(2.6E-11)	-0.038(1.9E-07)	0.057(5.8E-07)	0.056(8.9E-07)	-0.046(5.4E-05)	-0.055(1.7E-06)
86	Temporal_Mid_R	MTG.R	Temporal	0.044(1.9E-09)	-0.045(5.2E-10)	0.051(9.1E-06)	0.035(2.4E-03)	-0.018(1.2E-01)	-0.036(1.8E-03)
87	Temporal_Pole_Mid_L	TPOmid.L	Temporal	0.034(4.0E-06)	-0.023(1.5E-03)	0.017(1.4E-01)	0.035(2.4E-03)	-0.029(1.2E-02)	-0.030(8.5E-03)
88	Temporal_Pole_Mid_R	TPOmid.R	Temporal	0.047(1.4E-10)	-0.030(4.8E-05)	0.030(9.6E-03)	0.036(1.6E-03)	-0.013(2.4E-01)	-0.037(1.2E-03)
89	Temporal_Inf_L	ITG.L	Temporal	0.024(9.9E-04)	-0.025(5.1E-04)	0.038(9.3E-04)	0.031(6.7E-03)	-0.027(1.7E-02)	-0.034(3.0E-03)
90	Temporal_Inf_R	ITG.R	Temporal	0.017(2.3E-02)	-0.028(1.2E-04)	0.031(6.6E-03)	0.016(1.6E-01)	-0.025(3.0E-02)	-0.024(3.4E-02)
91	Cerebellum_Crus1_L	CRBLCrus1.L	Cerebellum	0.024(9.0E-04)	-0.007(3.1E-01)	0.013(2.6E-01)	0.021(6.4E-02)	-0.015(1.9E-01)	-0.014(2.1E-01)
92	Cerebellum_Crus1_R	CRBLCrus1.R	Cerebellum	0.028(1.6E-04)	-0.006(4.2E-01)	0.020(8.8E-02)	0.015(2.0E-01)	-0.008(4.7E-01)	-0.008(5.0E-01)

93	Cerebellum_Crus2_L	CRBLCrus2.L	Cerebellum	0.047(1.4E-10)	-0.004(6.1E-01)	0.028(1.4E-02)	0.045(8.1E-05)	-0.032(5.5E-03)	-0.042(2.5E-04)
94	Cerebellum_Crus2_R	CRBLCrus2.R	Cerebellum	0.043(3.2E-09)	-0.002(7.8E-01)	0.032(5.8E-03)	0.035(2.3E-03)	-0.020(7.5E-02)	-0.032(4.5E-03)
95	Cerebellum_3_L	CRBL3.L	Cerebellum	0.036(1.1E-06)	0.013(7.0E-02)	-0.016(1.5E-01)	-0.001(9.6E-01)	-0.005(6.9E-01)	0.001(9.6E-01)
96	Cerebellum_3_R	CRBL3.R	Cerebellum	0.032(9.9E-06)	-0.002(7.7E-01)	-0.010(3.9E-01)	-0.007(5.3E-01)	0.015(1.8E-01)	0.009(4.3E-01)
97	Cerebellum_4_5_L	CRBL45.L	Cerebellum	0.025(7.4E-04)	0.005(5.2E-01)	-0.016(1.6E-01)	-0.002(8.4E-01)	0.007(5.2E-01)	0.010(4.0E-01)
98	Cerebellum_4_5_R	CRBL45.R	Cerebellum	0.037(4.3E-07)	-0.003(7.2E-01)	0.001(9.5E-01)	0.008(4.8E-01)	0.008(4.6E-01)	0.007(5.6E-01)
99	Cerebellum_6_L	CRBL6.L	Cerebellum	0.025(5.9E-04)	-0.004(5.9E-01)	0.016(1.7E-01)	0.031(6.2E-03)	-0.021(6.1E-02)	-0.011(3.5E-01)
100	Cerebellum_6_R	CRBL6.R	Cerebellum	0.040(3.7E-08)	-0.006(4.0E-01)	0.020(8.4E-02)	0.022(5.0E-02)	-0.013(2.5E-01)	-0.002(8.4E-01)
101	Cerebellum_7b_L	CRBL7b.L	Cerebellum	0.049(2.5E-11)	0.001(8.4E-01)	0.031(6.9E-03)	0.039(7.9E-04)	-0.038(9.2E-04)	-0.042(2.5E-04)
102	Cerebellum_7b_R	CRBL7b.R	Cerebellum	0.058(1.9E-15)	-0.002(7.7E-01)	0.037(1.4E-03)	0.038(1.0E-03)	-0.036(1.5E-03)	-0.027(1.7E-02)
103	Cerebellum_8_L	CRBL8.L	Cerebellum	0.032(9.4E-06)	0.011(1.5E-01)	0.015(2.1E-01)	0.023(4.4E-02)	-0.026(2.2E-02)	-0.021(6.7E-02)
104	Cerebellum_8_R	CRBL8.R	Cerebellum	0.049(1.6E-11)	0.010(1.8E-01)	0.024(4.0E-02)	0.023(4.7E-02)	-0.030(8.7E-03)	-0.019(9.0E-02)
105	Cerebellum_9_L	CRBL9.L	Cerebellum	0.008(2.6E-01)	0.024(1.3E-03)	0.003(8.1E-01)	-0.004(7.1E-01)	-0.002(8.7E-01)	-0.011(3.5E-01)
106	Cerebellum_9_R	CRBL9.R	Cerebellum	0.011(1.2E-01)	0.025(8.2E-04)	0.002(8.7E-01)	-0.007(5.5E-01)	0.003(8.1E-01)	-0.001(9.1E-01)
107	Cerebellum_10_L	CRBL10.L	Cerebellum	-0.004(5.8E-01)	0.010(1.6E-01)	0.005(6.5E-01)	-0.005(6.6E-01)	0.001(9.2E-01)	-0.013(2.7E-01)
108	Cerebellum_10_R	CRBL10.R	Cerebellum	0.009(2.2E-01)	0.010(1.9E-01)	0.010(4.0E-01)	0.005(6.8E-01)	0.009(4.2E-01)	-0.012(3.1E-01)
109	Vermis_1_2	Vermis12	Cerebellum	0.042(1.3E-08)	0.008(3.0E-01)	-0.005(6.4E-01)	0.004(7.5E-01)	0.002(8.6E-01)	-0.005(6.9E-01)
110	Vermis_3	Vermis3	Cerebellum	0.041(1.8E-08)	0.009(2.0E-01)	-0.002(8.6E-01)	0.004(7.4E-01)	0.003(8.1E-01)	-0.004(7.0E-01)
111	Vermis_4_5	Vermis45	Cerebellum	0.018(1.3E-02)	0.016(2.8E-02)	-0.020(8.0E-02)	-0.011(3.2E-01)	0.007(5.5E-01)	0.006(5.9E-01)
112	Vermis_6	Vermis6	Cerebellum	0.006(3.9E-01)	0.015(4.1E-02)	-0.027(2.0E-02)	-0.006(6.2E-01)	-0.008(4.7E-01)	0.001(9.5E-01)
113	Vermis_7	Vermis7	Cerebellum	0.011(1.5E-01)	0.018(1.2E-02)	0.006(5.8E-01)	0.024(3.8E-02)	-0.012(2.9E-01)	-0.005(6.4E-01)
114	Vermis_8	Vermis8	Cerebellum	0.003(7.1E-01)	0.023(1.7E-03)	0.013(2.7E-01)	0.030(8.8E-03)	-0.010(3.7E-01)	-0.016(1.7E-01)
115	Vermis_9	Vermis9	Cerebellum	0.010(1.6E-01)	0.025(7.2E-04)	0.006(6.2E-01)	0.011(3.3E-01)	-0.004(7.3E-01)	0.004(7.0E-01)
116	Vermis_10	Vermis10	Cerebellum	-0.001(8.6E-01)	0.015(3.9E-02)	-0.008(4.9E-01)	-0.024(3.8E-02)	0.007(5.2E-01)	0.009(4.4E-01)
117	Thal_AV_L	Thal_AV_L	Subcortical	0.029(7.4E-05)	-0.016(3.1E-02)	0.020(7.9E-02)	0.008(4.7E-01)	-0.003(8.1E-01)	-0.003(7.9E-01)
118	Thal_AV_R	Thal_AV_R	Subcortical	0.031(2.2E-05)	-0.018(1.2E-02)	0.024(3.9E-02)	0.013(2.7E-01)	-0.005(6.8E-01)	0.012(3.0E-01)
119	Thal_LP_L	Thal_LP_L	Subcortical	0.044(2.2E-09)	-0.027(2.2E-04)	0.052(5.2E-06)	0.042(2.2E-04)	-0.024(3.8E-02)	-0.045(8.5E-05)
120	Thal_LP_R	Thal_LP_R	Subcortical	0.050(6.4E-12)	-0.040(3.3E-08)	0.048(3.0E-05)	0.053(3.9E-06)	-0.027(1.7E-02)	-0.030(9.3E-03)
121	Thal_VA_L	Thal_VA_L	Subcortical	-0.006(4.5E-01)	0.004(5.6E-01)	-0.028(1.5E-02)	-0.034(2.8E-03)	0.025(3.1E-02)	0.038(8.1E-04)
122	Thal_VA_R	Thal_VA_R	Subcortical	0.006(4.0E-01)	0.004(5.9E-01)	-0.014(2.3E-01)	-0.022(5.2E-02)	0.007(5.4E-01)	0.033(4.5E-03)
123	Thal_VL_L	Thal_VL_L	Subcortical	0.020(6.9E-03)	-0.007(3.2E-01)	0.027(2.0E-02)	0.012(3.1E-01)	-0.007(5.4E-01)	-0.018(1.2E-01)
124	Thal_VL_R	Thal_VL_R	Subcortical	0.040(5.6E-08)	-0.023(1.4E-03)	0.052(6.7E-06)	0.041(3.1E-04)	-0.030(9.0E-03)	-0.023(4.2E-02)

125	Thal_VPL_L	Thal_VPL_L	Subcortical	0.033(4.6E-06)	-0.036(8.4E-07)	0.061(9.6E-08)	0.075(4.8E-11)	-0.034(2.9E-03)	-0.058(4.8E-07)
126	Thal_VPL_R	Thal_VPL_R	Subcortical	0.040(4.8E-08)	-0.044(2.2E-09)	0.053(3.6E-06)	0.072(3.2E-10)	-0.027(2.0E-02)	-0.041(3.1E-04)
127	Thal_IL_L	Thal_IL_L	Subcortical	0.056(1.2E-14)	-0.052(7.6E-13)	0.070(1.2E-09)	0.091(1.9E-15)	-0.051(9.7E-06)	-0.075(6.5E-11)
128	Thal_IL_R	Thal_IL_R	Subcortical	0.057(6.9E-15)	-0.058(1.8E-15)	0.060(1.7E-07)	0.086(4.8E-14)	-0.038(8.7E-04)	-0.056(1.1E-06)
129	Thal_Re_L	Thal_Re_L	Subcortical	0.036(9.3E-07)	0.002(7.7E-01)	-0.004(7.5E-01)	0.014(2.2E-01)	0.012(3.1E-01)	0.009(4.4E-01)
130	Thal_Re_R	Thal_Re_R	Subcortical	0.043(4.8E-09)	-0.018(1.3E-02)	0.033(4.5E-03)	0.048(2.9E-05)	-0.013(2.7E-01)	-0.011(3.3E-01)
131	Thal_MDm_L	Thal_MDm_L	Subcortical	0.063(4.2E-18)	-0.062(2.2E-17)	0.072(4.1E-10)	0.087(2.4E-14)	-0.045(9.5E-05)	-0.070(1.1E-09)
132	Thal_MDm_R	Thal_MDm_R	Subcortical	0.059(8.3E-16)	-0.064(2.1E-18)	0.061(1.0E-07)	0.085(9.4E-14)	-0.035(2.5E-03)	-0.052(6.1E-06)
133	Thal_MDl_L	Thal_MDl_L	Subcortical	0.063(1.0E-17)	-0.054(1.1E-13)	0.082(1.0E-12)	0.098(1.1E-17)	-0.056(7.8E-07)	-0.084(2.3E-13)
134	Thal_MDl_R	Thal_MDl_R	Subcortical	0.059(6.2E-16)	-0.061(4.6E-17)	0.068(4.1E-09)	0.093(3.5E-16)	-0.044(1.4E-04)	-0.062(4.7E-08)
135	Thal_LGN_L	Thal_LGN_L	Subcortical	0.049(1.5E-11)	-0.017(2.1E-02)	0.015(1.9E-01)	0.018(1.1E-01)	-0.027(1.7E-02)	-0.011(3.4E-01)
136	Thal_LGN_R	Thal_LGN_R	Subcortical	0.058(2.4E-15)	-0.025(6.6E-04)	0.014(2.1E-01)	0.038(9.7E-04)	-0.020(8.0E-02)	-0.013(2.6E-01)
137	Thal_MGN_L	Thal_MGN_L	Subcortical	0.061(3.9E-17)	-0.040(5.7E-08)	0.037(1.3E-03)	0.055(1.6E-06)	-0.035(2.0E-03)	-0.036(1.5E-03)
138	Thal_MGN_R	Thal_MGN_R	Subcortical	0.058(2.9E-15)	-0.042(1.1E-08)	0.031(7.0E-03)	0.054(2.1E-06)	-0.021(6.0E-02)	-0.027(1.8E-02)
139	Thal_PuA_L	Thal_PuA_L	Subcortical	0.062(3.0E-17)	-0.043(3.9E-09)	0.039(7.1E-04)	0.057(6.1E-07)	-0.033(3.5E-03)	-0.039(7.5E-04)
140	Thal_PuA_R	Thal_PuA_R	Subcortical	0.061(8.7E-17)	-0.046(3.7E-10)	0.035(2.4E-03)	0.063(3.3E-08)	-0.024(3.6E-02)	-0.035(2.3E-03)
141	Thal_PuM_L	Thal_PuM_L	Subcortical	0.059(1.1E-15)	-0.055(5.2E-14)	0.058(5.0E-07)	0.076(2.8E-11)	-0.039(6.6E-04)	-0.062(5.6E-08)
142	Thal_PuM_R	Thal_PuM_R	Subcortical	0.056(1.4E-14)	-0.055(4.2E-14)	0.042(2.4E-04)	0.071(4.7E-10)	-0.025(3.0E-02)	-0.045(7.5E-05)
143	Thal_PuL_L	Thal_PuL_L	Subcortical	0.056(2.5E-14)	-0.058(1.7E-15)	0.073(2.4E-10)	0.096(6.6E-17)	-0.048(2.8E-05)	-0.080(2.8E-12)
144	Thal_PuL_R	Thal_PuL_R	Subcortical	0.053(2.7E-13)	-0.059(6.7E-16)	0.054(2.3E-06)	0.086(8.5E-14)	-0.034(2.9E-03)	-0.057(5.6E-07)
145	Thal_PuI_L	Thal_PuI_L	Subcortical	0.024(1.0E-03)	-0.028(1.1E-04)	0.044(1.1E-04)	0.041(4.1E-04)	-0.014(2.1E-01)	-0.038(9.6E-04)
146	Thal_PuI_R	Thal_PuI_R	Subcortical	0.029(6.4E-05)	-0.031(2.4E-05)	0.024(4.0E-02)	0.034(3.5E-03)	-0.003(7.9E-01)	-0.011(3.5E-01)
147	ACC_sub_L	ACC_sub_L	Frontal	0.039(1.2E-07)	-0.030(4.1E-05)	-0.006(6.0E-01)	-0.001(9.1E-01)	0.006(5.7E-01)	0.001(9.4E-01)
148	ACC_sub_R	ACC_sub_R	Frontal	0.030(3.4E-05)	-0.029(7.6E-05)	0.001(9.6E-01)	0.007(5.5E-01)	0.004(7.2E-01)	-0.007(5.5E-01)
149	ACC_pre_L	ACC_pre_L	Frontal	0.052(1.1E-12)	-0.044(2.2E-09)	0.029(1.2E-02)	0.028(1.4E-02)	-0.022(5.3E-02)	-0.041(3.7E-04)
150	ACC_pre_R	ACC_pre_R	Frontal	0.046(2.6E-10)	-0.038(3.0E-07)	0.036(1.9E-03)	0.042(2.8E-04)	-0.025(2.9E-02)	-0.042(2.4E-04)
151	ACC_sup_L	ACC_sup_L	Frontal	0.053(3.4E-13)	-0.048(6.5E-11)	0.025(2.7E-02)	0.035(2.4E-03)	-0.016(1.7E-01)	-0.036(1.8E-03)
152	ACC_sup_R	ACC_sup_R	Frontal	0.030(4.1E-05)	-0.031(2.8E-05)	0.024(4.0E-02)	0.029(1.3E-02)	-0.022(5.6E-02)	-0.040(4.1E-04)
153	N_Acc_L	N_Acc_L	Brainstem	0.039(1.0E-07)	-0.021(4.5E-03)	0.021(6.3E-02)	0.022(5.3E-02)	-0.002(8.9E-01)	-0.004(7.2E-01)
154	N_Acc_R	N_Acc_R	Brainstem	0.031(2.5E-05)	-0.026(3.9E-04)	0.013(2.4E-01)	0.013(2.6E-01)	0.003(8.2E-01)	-0.006(6.2E-01)
155	VTA_L	VTA_L	Brainstem	0.043(3.1E-09)	-0.023(1.4E-03)	0.023(4.1E-02)	0.009(4.3E-01)	-0.011(3.4E-01)	-0.013(2.7E-01)
156	VTA_R	VTA_R	Brainstem	0.052(1.8E-12)	-0.020(7.0E-03)	0.028(1.4E-02)	0.016(1.7E-01)	-0.014(2.3E-01)	-0.008(4.8E-01)

157	SN_pc_L	SN_pc_L	Brainstem	0.033(5.3E-06)	-0.018(1.3E-02)	0.000(9.8E-01)	0.029(1.0E-02)	0.007(5.4E-01)	-0.010(4.0E-01)
158	SN_pc_R	SN_pc_R	Brainstem	0.036(6.5E-07)	-0.026(3.3E-04)	0.007(5.3E-01)	0.025(2.7E-02)	0.012(2.8E-01)	0.008(4.9E-01)
159	SN_pr_L	SN_pr_L	Brainstem	0.020(5.5E-03)	-0.005(5.1E-01)	-0.010(3.7E-01)	0.024(3.5E-02)	0.011(3.3E-01)	0.007(5.5E-01)
160	SN_pr_R	SN_pr_R	Brainstem	0.019(8.9E-03)	-0.020(5.2E-03)	-0.003(8.3E-01)	0.016(1.6E-01)	0.021(7.3E-02)	0.019(1.0E-01)
161	Red_N_L	Red_N_L	Brainstem	0.048(7.0E-11)	-0.015(4.2E-02)	0.010(4.0E-01)	0.023(4.3E-02)	-0.003(8.2E-01)	-0.012(2.9E-01)
162	Red_N_R	Red_N_R	Brainstem	0.046(2.5E-10)	-0.010(1.6E-01)	0.019(9.2E-02)	0.020(8.2E-02)	0.000(9.8E-01)	0.000(9.7E-01)
163	LC_L	LC_L	Brainstem	0.020(5.3E-03)	0.008(3.0E-01)	-0.011(3.5E-01)	-0.018(1.1E-01)	0.004(7.3E-01)	0.014(2.3E-01)
164	LC_R	LC_R	Brainstem	0.024(8.1E-04)	0.003(7.2E-01)	-0.011(3.3E-01)	-0.018(1.2E-01)	0.017(1.3E-01)	0.014(2.1E-01)
165	Raphe_D	Raphe_D	Brainstem	0.068(9.7E-21)	-0.025(7.3E-04)	0.051(9.8E-06)	0.064(2.1E-08)	-0.033(3.9E-03)	-0.056(8.1E-07)
166	Raphe_M	Raphe_M	Brainstem	0.050(9.0E-12)	-0.010(1.9E-01)	0.010(4.1E-01)	-0.011(3.5E-01)	0.007(5.5E-01)	0.001(9.2E-01)

Notes: The table reports the GMV-association patterns of the follow-up cognitive functions and cereal/coffee intake in the discovery sample. Each cell refers to the correlation coefficient and P value.
